# Screening for immune-related biomarkers associated with myasthenia gravis and dilated cardiomyopathy based on bioinformatics analysis and machine learning

**DOI:** 10.1016/j.heliyon.2024.e28446

**Published:** 2024-03-20

**Authors:** Guiting Zhou, Shushu Wang, Liwen Lin, Kachun Lu, Zhichao Lin, Ziyan Zhang, Yuling Zhang, Danling Cheng, KaMan Szeto, Rui Peng, Chuanjin Luo

**Affiliations:** a The First Clinical Medical College, Guangzhou University of Chinese Medicine, Guangzhou, China; b Zhongshan Traditional Chinese Medicine Hospital, Zhongshan, China; c Shunde Hospital of Guangzhou University of Chinese Medicine, Foshan, China; d Cardiology Center, The First Affiliated Hospital of Guangzhou University of Chinese Medicine, Guangzhou, China; e Guangdong Clinical Research Academy of Chinese Medicine, Guangzhou, China

**Keywords:** Dilated cardiomyopathy, Myasthenia gravis, Immune cell infiltration, Diagnostic value, Machine learning algorithms

## Abstract

**Background:**

We aim to investigate genes associated with myasthenia gravis (MG), specifically those potentially implicated in the pathogenesis of dilated cardiomyopathy (DCM). Additionally, we seek to identify potential biomarkers for diagnosing myasthenia gravis co-occurring with DCM.

**Methods:**

We obtained two expression profiling datasets related to DCM and MG from the Gene Expression Omnibus (GEO). Subsequently, we conducted differential gene expression analysis and weighted gene co-expression network analysis (WGCNA) on these datasets. The genes exhibiting differential expression common to both DCM and MG were employed for protein-protein interaction (PPI), Gene Ontology (GO) enrichment analysis, and Kyoto Encyclopedia of Genes and Genomes (KEGG) pathway analysis. Additionally, machine learning techniques were employed to identify potential biomarkers and develop a diagnostic nomogram for predicting MG-associated DCM. Subsequently, the machine learning results underwent validation using an external dataset. Finally, gene set enrichment analysis (GSEA) and machine algorithm analysis were conducted on pivotal model genes to further elucidate their potential mechanisms in MG-associated DCM.

**Results:**

In our analysis of both DCM and MG datasets, we identified 2641 critical module genes and 11 differentially expressed genes shared between the two conditions. Enrichment analysis disclosed that these 11 genes primarily pertain to inflammation and immune regulation. Connectivity map (CMAP) analysis pinpointed SB-216763 as a potential drug for DCM treatment. The results from machine learning indicated the substantial diagnostic value of midline 1 interacting protein1 (MID1IP1) and PI3K-interacting protein 1 (PIK3IP1) in MG-associated DCM. These two hub genes were chosen as candidate biomarkers and employed to formulate a diagnostic nomogram with optimal diagnostic performance through machine learning. Simultaneously, single-gene GSEA results and immune cell infiltration analysis unveiled immune dysregulation in both DCM and MG, with MID1IP1 and PIK3IP1 showing significant associations with invasive immune cells.

**Conclusion:**

We have elucidated the inflammatory and immune pathways associated with MG-related DCM and formulated a diagnostic nomogram for DCM utilizing MID1IP1/PIK3IP1. This contribution offers novel insights for prospective diagnostic approaches and therapeutic interventions in the context of MG coexisting with DCM.

## Highlights

• Our investigation unveils immune dysfunction as the focal point in the pathogenesis of MG and DCM.
• We identified MID1IP1 and PIK3IP1 as pivotal crosstalk genes in MG-associated DCM, exhibiting robust diagnostic effectiveness.
• Immune infiltration analysis has elucidated the correlation between MID1IP1 and PIK3IP1 and infiltrating immune cells.

## Graphical abstract

**Figure:**
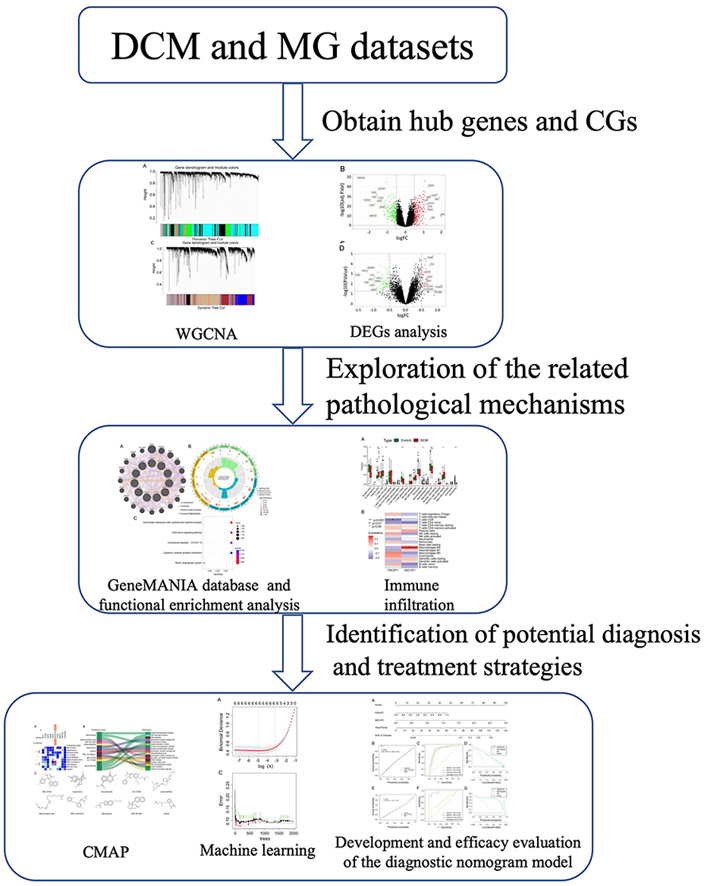

## 1 Introduction

Myasthenia gravis (MG) is a rare autoimmune disease marked by an autoimmune assault on the neuromuscular junction. This assault disrupts the transmission of nerve impulses to the muscles, resulting in muscle fatigue and weakness [1]. Dilated cardiomyopathy (DCM) is a cardiac disorder characterized by ventricular dilation, thinning of the myocardial walls, and impaired cardiac contractile function, leading to compromised pumping of the heart [2]. DCM is often accompanied by cardiac enlargement, thinning of the ventricular walls, and increased cavity volume, ultimately causing a decrease in cardiac contractility. These pathological changes can result in a spectrum of clinical symptoms such as heart failure and arrhythmias, potentially culminating in heart failure. Despite the seemingly distinct clinical manifestations and pathophysiological mechanisms of MG and DCM, recent research indicates a potential association between the two conditions [3].

Early clinical cases and specific epidemiological studies suggest abnormal cardiac function in a subset of MG patients [4,5]. In thymoma-associated and late-onset MG, numerous case reports highlight severe myocarditis leading to heart failure and cardiac conduction abnormalities, indicating potential autoimmune involvement in the myocardium [6]. Owing to the pronounced skeletal muscle weakness symptoms, cardiac damage in MG patients is frequently overlooked. By the time severe cardiac complications emerge, reversing the disease becomes challenging. Timely intervention is crucial for improving the prognosis of MG-associated DCM; however, challenges persist in early detection. Despite utilizing endomyocardial biopsy, accurately detecting MG-related myocardial damage remains challenging due to its limited sensitivity [7]. Proposals for monitoring cardiac function during acute MG attacks, particularly in patients with positive anti-muscle antibodies, have been made [8]. Nevertheless, once cardiac function declines, reversing myocardial damage becomes difficult. From a pathogenesis standpoint, numerous enigmas persist regarding MG-associated dilated cardiomyopathy. This comorbid condition involves intricate interactions, including genetics, immunology, and cell biology, contributing to the complexity of early diagnosis. Furthermore, understanding the comorbidity mechanisms between MG and DCM is crucial for enhancing diagnostic and therapeutic efficacy, as it may result in variations in disease progression and adjustments in treatment strategies.

This study utilized integrated bioinformatics tools on two DCM datasets and one MG dataset from GEO to uncover hub genes and potential mechanisms in MG-related DCM. Identified potential therapeutic compounds for DCM and validated gene expression patterns using machine learning. Constructed a diagnostic nomogram (MID1IP1 and PIK3IP1) for DCM, evaluating its efficiency with an external dataset. Additionally, explored DCM's immune cell characteristics to understand the link between hub genes and the immune landscape.

## 2 Method

### 2.1 Microarray data collection and processing

The two original expression profile datasets for DCM and the control group, including GSE57338 and GSE29819, were downloaded from the GEO database (https://www.ncbi.nlm.nih.gov/geo/). Additionally, the raw expression profile dataset for MG and control groups, GSE85452, was also downloaded from the GEO database. The GSE57338 dataset (platform: GPL11532) consists of 132 control samples and 82 DCM samples. The GSE29819 dataset (platform: GPL570) includes 6 control samples and 7 DCM samples. Lastly, the GSE85452 dataset (platform: GPL10558) comprises 13 MG samples and 12 healthy samples. These datasets will serve as the basis for our analysis.

### 2.2 Weighted gene co-expression network analysis and identification of key module genes

We employed the "WGCNA" package to construct scale-free co-expression gene networks for GSE57338 and GSE85452 datasets. The median absolute deviation (MAD) of each gene was calculated for both datasets, and the top 5000 genes based on MAD were selected. The "goodSamplesGenes" function was utilized to examine missing entries, items with low weights, and zero-variance genes in the data. It returned lists of samples and genes with the highest standards of missing values or low-weight values. Furthermore, for this experiment, the soft-power values for both datasets were set at 6 and 14 as the weights. The module-feature matrix and sample information matrix were computed, and the correlation between them was visualized using the "labeledHeatmap" function. This visualization helped identify the most significant modules in terms of positive and negative correlations with features. Finally, the key module genes from both datasets were merged.

### 2.3 Differentially expressed genes (DEG) analysis

The differential expression analysis of the DCM dataset and MG dataset (GSE57338 and GSE85452) was performed using the "Limma" package in R software, with a cutoff criteria of *P* < 0.05 and |log (FC)| > 0.5. Subsequently, the expression patterns of the DEGs were visualized using the "pheatmap" and "ggplot2" packages in R software, generating heatmaps and volcano plots, respectively.

### 2.4 Analysis of the GeneMANIA database

To explore the interactions of differentially expressed genes between MG and DCM, we constructed networks using the GeneMANIA database (http://genemania.org/). The networks were based on co-expression, physical interactions, shared protein domains, and predicted interactions among the common genes (CGs). We employed the "assigned based on query GeneMANIA" strategy to maximize the connectivity among all input genes. This strategy utilizes linear regression to automatically select weights, aiming to maximize interactions among the genes in the list while minimizing interactions with genes not in the list.

### 2.5 Functional enrichment analysis

To investigate the biological functions and specific mechanisms of pathogenic genes related to MG and DCM, we GO and KEGG pathway enrichment analysis on the candidate genes using packages such as "org.Hs.eg.db", "GOplot", "enrichplot", and "clusterProfiler". A threshold of *p* < 0.05 was considered significant for enrichment. Furthermore, we visualized the results of the enrichment analysis using packages such as "ggplot2", "circlize", "RColorBrewer", and "ComplexHeatmap". The visualization included circular plots and bubble plots to display the results of functional enrichment analysis.

### 2.6 Connectivity map analysis

CMAP (https://clue.io) is a gene expression profile database based on the perturbation of gene expression features. It has high predictive value in uncovering the relationship between genes and small molecules. In this study, DEGs co-expressed in DCM and MG were included in the CMAP online database to identify potential small-molecule drugs for DCM treatment. Finally, the top 10 compounds with the highest enrichment scores were identified. Using the "ggalluvial" package, a Sankey diagram was created based on the descriptions of these compounds.

### 2.7 Machine learning

To identify candidate biomarkers and establish a diagnostic model for DCM, we took the intersection of DEGs co-expressed in DCM and MG, as well as the key module genes obtained from WGCNA. The resulting common genes were subjected to the least absolute shrinkage and selection operator (LASSO) regression analysis [9] using the "glmnet" package to further screen for candidate biomarkers. Simultaneously, the "randomForest" package was employed to analyze the common genes using the random forest (RF) algorithm [10,11], aiming to further narrow down the range of candidate biomarkers. Genes that overlapped in the LASSO model and had MeanDecreaseGini values greater than 6 in the RF model were defined as hub genes for developing a diagnostic model for MG-related DCM.

### 2.8 Validation of the expression levels of key genes in DCM and MG

Following the LASSO regression analysis and RF algorithm analysis, key genes with diagnostic values were selected. The expression levels of these genes in the DCM dataset and MG dataset were compared and visualized using the "rstatix", "ggsignif", "ggplot2", and "ggpubr" packages. This analysis aimed to identify genes that were commonly upregulated or downregulated in both DCM and MG patients. By comparing and visualizing the gene expression levels, we were able to identify genes that showed consistent upregulation or downregulation in both DCM and MG patients.

### 2.9 Construction of nomograms and evaluation of predictive models for diagnostic markers

Further selection was carried out by conducting logistic regression analysis on the genes C3AR1, MID1IP1, and PIK3IP1, which showed the same expression trend. These genes served as common hub genes for both diseases. The "rms" package was utilized to construct column plots to visualize the results. Receiver operating characteristic (ROC) curves were generated to evaluate the performance of each hub gene and the nomograms in diagnosing DCM. Additionally, ROC curves were used to determine whether the decision based on the nomograms was beneficial for DCM diagnosis. Calibration curves and decision curve analysis (DCA) were employed to assess the predictive efficiency of the nomograms for MG-related DCM. Finally, the predictive efficiency of the nomograms was validated using an external DCM dataset GSE29819.

### 2.10 Single-gene GSEA

In the GSE57338 dataset, the model genes obtained from the aforementioned methods were subjected to GSEA [12] and visualization using packages such as "org.Hs.eg.db", "ggsci", "patchwork", and "ggplot2". The genes were grouped based on their expression levels to evaluate the significant pathways that impact the disease.

### 2.11 Analysis of immune infiltration

The evaluation of immune cell infiltration in the gene expression profiles of DCM and MG was performed using the "CIBERSORT" package. The abundance and proportions of immune infiltration for each sample were visualized as bar plots using the "ggplot2" package. The Wilcoxon test was employed to compare the differences in the proportions of 22 immune cell types between DCM and normal samples. The results were displayed using stacked histograms generated by the "ggplot2" package. Furthermore, the correlations among the 22 infiltrating immune cells were depicted using the "corrplot" package, with a significance level of *p* < 0.05 considered statistically significant.

### 2.12 Statistical analysis

GraphPad Prism version 9.0.2 (GraphPad Software Inc., San Diego, CA, USA) was used for statistical analysis. Results were displayed as mean ± SD. Differences between the two groups were compared by unpaired Student's t-test. *P* < 0.05 was regarded as statistical significance.

## 3 Results

### 3.1 Weighted gene co-expression network analysis and identification of key module genes

**Fig. 1:**
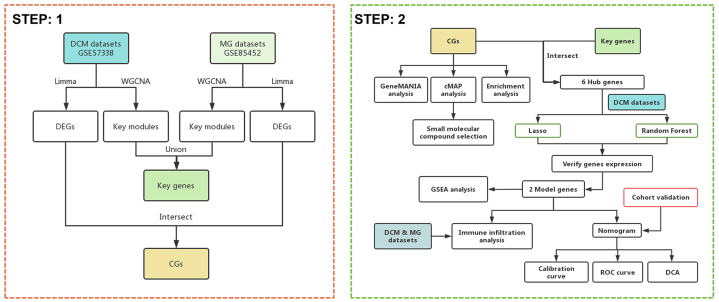
Flow chart of this study design. DCM: Dilated cardiomyopathy, MG: Myasthenia gravis, WGCNA: Weighted gene co-expression network analysis, DEGs: Differentially expressed genes, CGs: Common genes, ROC: Receiver operating characteristic, DCA: Decision curve analysis.

**Fig. 2:**
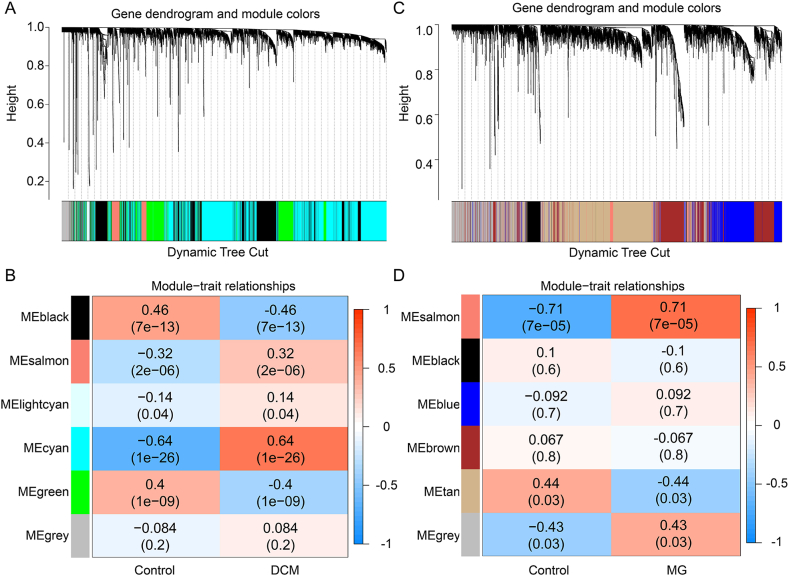
Identification of key module genes in the integrated DCM and MG dataset through WGCNA. A: Dendrogram of gene clustering in DCM, with different colors representing different modules. B: Correlation between module eigengenes and DCM, where blue indicates negative correlation and red indicates positive correlation. C: Dendrogram of gene clustering in MG, with different colors representing different modules. D: Correlation between module eigengenes and MG, where blue indicates a negative correlation and red indicates a positive correlation. DCM: Dilated cardiomyopathy, MG: Myasthenia gravis, WGCNA: Weighted gene co-expression network analysis. (For interpretation of the references to color in this figure legend, the reader is referred to the Web version of this article.)

The strategy of bioinformatics analysis is performed as shown in Fig. 1. To explore the key genes in DCM and MG, we performed WGCNA to identify the most relevant gene modules in both DCM and MG samples. In the DCM-WGCNA analysis, a soft threshold of 6 was chosen based on scale independence and average connectivity, generating a total of 6 modules. The dendrogram of module clustering is presented in Fig. 2A. Furthermore, the correlation between DCM and gene modules was examined (Fig. 2B). The data revealed that the cyan module showed the highest positive correlation with DCM (2541 genes, r = 0.64, *p* = 1e−26). Based on this, the cyan module was considered the key module for subsequent analysis. In the MG-WGCNA analysis, a soft threshold of 14 was selected, resulting in 6 modules (Fig. 2C). Similarly, the correlation between MG and gene modules was explored (Fig. 2D). The data demonstrated that the salmon module exhibited the highest positive correlation with MG (114 genes, r = 0.71, *p* = 7e−05). Therefore, the salmon module was considered the key module for further analysis. In addition, the module genes obtained from WGCNA analysis in the DCM and MG datasets were deduplicated and merged, resulting in 2641 key genes (referred to as Key genes) for subsequent analysis.

### 3.2 Differential expression gene analysis

**Fig. 3:**
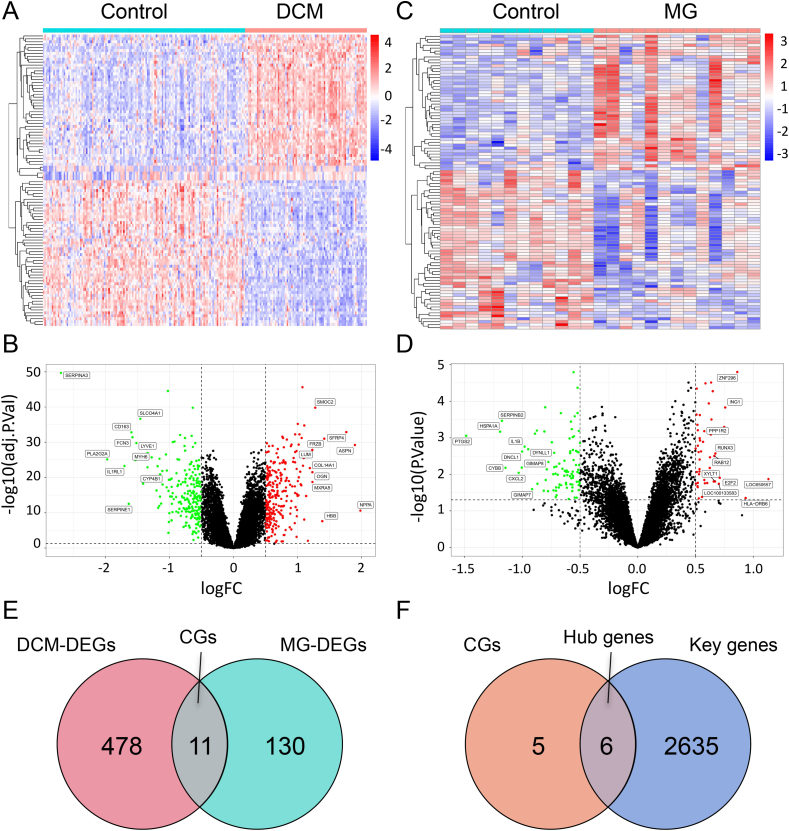
Differential expression analysis of the DCM and MG datasets. A: Heatmap of the top 25 upregulated and 25 downregulated DEGs in the DCM dataset. B: Volcano plot of DEGs in the DCM dataset, with green indicating downregulation and red indicating upregulation. C: Heatmap of the top 25 upregulated and 25 downregulated DEGs in the MG dataset. D: Volcano plot of DEGs in the MG dataset, with green indicating downregulation and red indicating upregulation. E: Venn diagram showing the intersection of DEGs in DCM and MG, named CGs. F: Key genes obtained by aggregating and de-duplicating WGCNA key module genes in DCM and MG, intersected with CGs using a Venn diagram, named Hub genes. DCM: Dilated cardiomyopathy, MG: Myasthenia gravis, DEGs: Differentially expressed genes, CGs: Common genes. (For interpretation of the references to color in this figure legend, the reader is referred to the Web version of this article.)

The differential analysis between DCM and normal samples revealed a total of 489 DEGs, including 269 upregulated genes and 220 downregulated genes. For the MG dataset, there are 140 DEGs, consisting of 46 upregulated genes and 94 downregulated genes. The expression patterns of DEGs in the DCM and MG datasets were depicted using volcano plots and heatmaps (Fig. 3A, B, C, D). By taking the intersection of DEGs expressed in both DCM and MG samples using the "VennDiagram" package, 11 CGs were obtained (Fig. 3E). Furthermore, we obtained 6 Hub genes by intersecting the CGs with the previously identified Key genes (Fig. 3F).

### 3.3 GeneMANIA database analysis

**Fig. 4:**
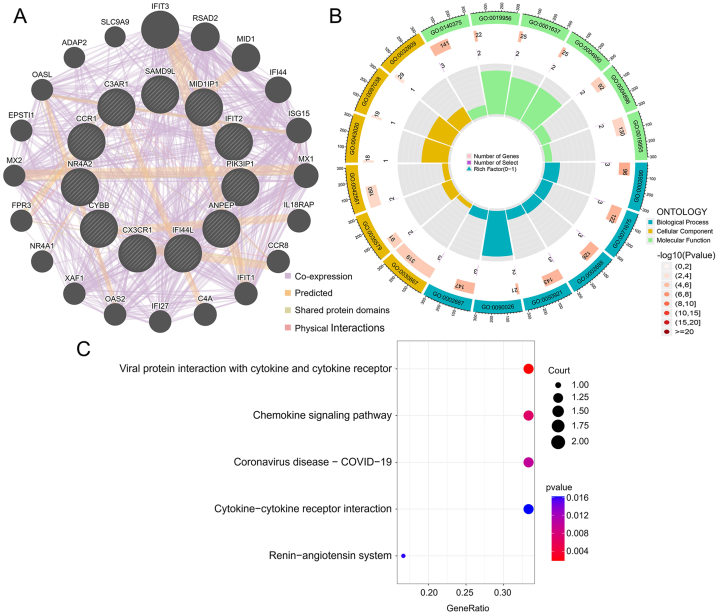
GeneMANIA database analysis and functional enrichment analysis based on CGs. A: GeneMANIA database analysis of CGs, showing a network with a total of 31 genes and 721 connections, including Co-expression, Physical Interaction, Shared Protein Domains, and Predicted networks. B: Circular plot of the results of GO enrichment analysis based on CGs. C: Bubble plot of the results of KEGG enrichment analysis based on CGs. CGs: Common genes, GO Gene ontology, KEGG: Kyoto Encyclopedia of Genes and Genomes.

To uncover potential disease-causing genes and mechanisms in MG-associated DCM, we utilized the GeneMANIA database to analyze the interactions among the DEGs shared between MG and DCM. As shown in Fig. 4A, the nodes represent the genes we uploaded as well as the genes related to our uploaded genes identified through the GeneMANIA search. The lines represent the network categories between these genes. In our network, there are a total of 31 genes, including the 11 uploaded genes and 20 associated genes. There are 721 connections in total, including Co-expression, Physical Interaction, Shared protein domains, and Predicted networks.

### 3.4 Functional enrichment analysis

To gain a better understanding of the functions and specific mechanisms of these disease-causing genes, we conducted functional enrichment and KEGG pathway analysis on the 11 common differentially expressed genes. The GO analysis of biological processes (BP) revealed that the disease-causing genes in MG-associated DCM are mainly enriched in terms such as "positive regulation of vasculature development", "regulation of mononuclear cell migration", and "macrophage activation involved in immune response". In the GO analysis of cellular components (CC), these disease-causing genes were found to be primarily located in the "secretory granule membrane" and "specific granule membrane". In terms of molecular function (MF) analysis, the results indicated that "immune receptor activity" and "chemokine binding" were the most relevant functions among the disease-causing genes (Fig. 4B). The KEGG pathway analysis demonstrated a close association between the disease-causing genes in MG-associated DCM and pathways such as "Viral protein interaction with cytokine and cytokine receptor", "Chemokine signaling pathway", "Renin-angiotensin system", and "Cytokine-cytokine receptor interaction" (Fig. 4C).

### 3.5 Identification of potential small-molecule compounds for DCM treatment

**Fig. 5:**
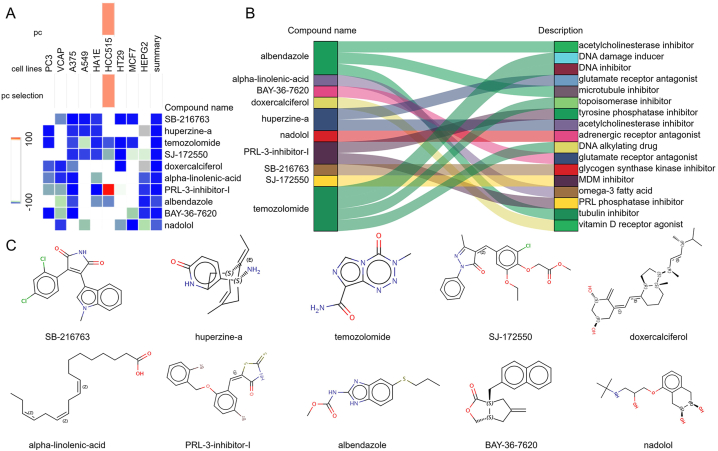
Screening of potential small molecule compounds for DCM treatment using connectivity map analysis. A: Heatmap displaying the top 10 compounds with the highest enrichment scores across 9 cell lines based on connectivity map analysis. B: Descriptions of the top 10 compounds. C: Chemical structures of these 10 compounds.

To further investigate potential small-molecule drugs that may have therapeutic effects on MG-associated DCM patients, we input the DEGs shared between MG and DCM into the CMAP database to predict small-molecule compounds that can reverse the gene expression associated with DCM. Using the CMAP website (https://clue.io/query) and the CGs for prediction, a total of 8,559 results were obtained, including 2,429 compounds. After removing drugs without targets, the top ten compounds with the most negative scores were selected based on the median tau score. These compounds include SB-216763, huperzine-a, temozolomide, SJ-172550, doxercalciferol, alpha-linolenic-acid, PRL-3-inhibitor-I, albendazole, BAY-36-7620, and nadolol. They have the highest negative scores and are considered potential therapeutic agents for MG-associated DCM treatment (Fig. 5A). The targeted pathways and chemical structures of these ten compounds are described in Fig. 5B and C, respectively.

### 3.6 Identification of key genes with diagnostic value through machine learning

**Fig. 6:**
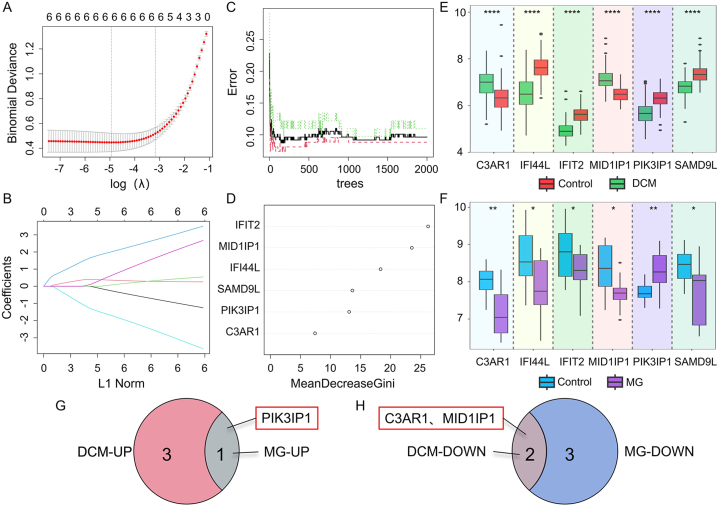
Screening of potential diagnostic biomarkers for MG-associated DCM using machine learning methods. A–B: Lasso regression analysis of the 6 hub genes to calculate the minimum value (A) and λ value (B) for diagnostic biomarkers. C–D: Random forest algorithm analysis of the 6 hub genes, with a Random forest plot generated; selection of biomarkers with Mean Decrease Gini scores greater than 6. E: Expression patterns of the 6 hub genes in the DCM dataset GSE57338. F: Expression patterns of the 6 hub genes in the MG dataset GSE85452. G: Venn diagram showing the upregulated genes expressed by the 6 Hub genes in the DCM and MG datasets, respectively. H: Venn diagram showing the downregulated genes expressed by the 6 hub genes in the DCM and MG datasets, respectively. DCM: Dilated cardiomyopathy, MG: Myasthenia gravis.

Since the DEGs shared between DCM and MG may play a crucial role in MG-associated DCM patients, the LASSO regression algorithm was applied to the 6 CGs, and the results showed all these genes were potential candidate genes with a significant impact on diagnosing MG-associated DCM patients (Fig. 6A and B). To further narrow the scope of diagnostic biomarkers, we also utilized an RF machine learning algorithm. The six CGs were ranked based on their variable importance, and genes with a MeanDecreaseGini value greater than 6 were selected (Fig. 6C and D). Interestingly, these six genes still retained their importance in diagnosing the disease according to the RF model.

### 3.7 Validation of the expression levels of key genes in DCM and MG

As shown in Fig. 6E, in the DCM dataset (GSE57338), the expression levels of C3AR1 and MID1IP1 in DCM patients were significantly lower compared to the control group (*P* < 0.05). On the other hand, the expression levels of IFIT2, SAMD9L, IFI44L, and PIK3IP1 were significantly higher in DCM patients compared to the control group (*P* < 0.05). Similarly, in the MG dataset (GSE85452), as depicted in Fig. 6F, the expression levels of C3AR1, IFIT2, IFI44L, SAMD9L, and MID1IP1 in MG patients were significantly lower compared to the control group (*P* < 0.05). However, the expression level of PIK3IP1 was significantly higher in MG patients compared to the control group *(P* < 0.05). Therefore, it can be concluded that the expression levels of C3AR1 and MID1IP1 are downregulated in both DCM and MG patients, while PIK3IP1 is upregulated.

### 3.8 Construction of nomograms and evaluation of diagnostic biomarker prediction model

**Fig. 7:**
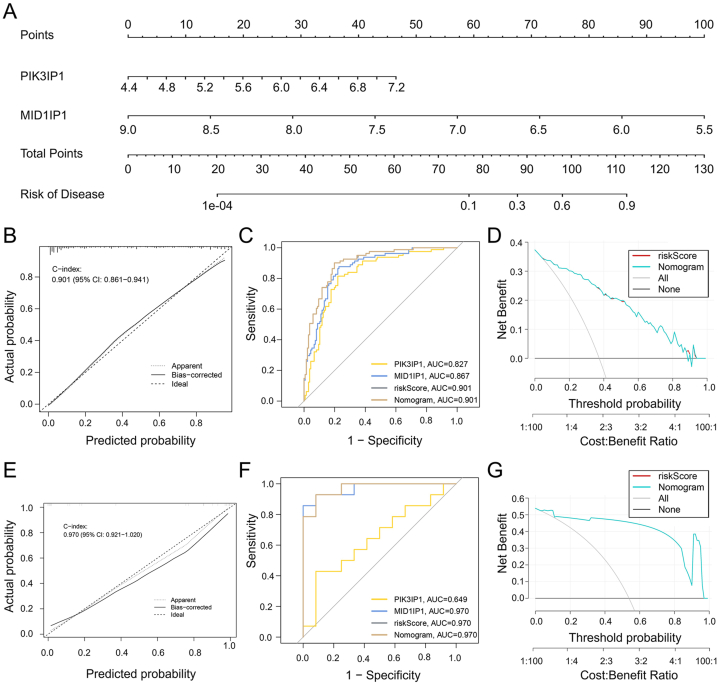
Development and efficacy evaluation of the diagnostic nomogram model. A: Logic regression analysis of 3 genes, including C3AR1, PIK3IP1, and MID1IP1, followed by further screening to obtain the PIK3IP1 and MID1IP1 genes as the two key genes for constructing the diagnostic nomogram. B: Calibration curve of the nomogram model predictions in MG-associated DCM, where the dashed line labeled "Ideal" represents the standard curve, representing perfect predictions of the ideal model. The dotted line labeled "Apparent" represents the uncalibrated predicted curve, while the solid line labeled "Bias-corrected" represents the calibrated predicted curve. C: ROC ROC curve for the diagnostic performance of the two candidate biomarkers (PIK3IP1 and MID1IP1). D: DCA for the nomogram model. The black line is labeled as "None," representing the net benefit of the assumption that no patients have DCM. The grey line is labeled as "All," indicating the net benefit of the assumption that all patients have DCM, and the purple line is labeled as "Nomogram," representing the net benefit of the assumption that MG-related DCM cases are identified based on the diagnostic value of DCM predicted by the nomogram model. E–G: Calibration curve, ROC curve, and DCA decision curve for the nomogram in the external dataset GSE29819 of DCM. DCM: Dilated cardiomyopathy, MG: Myasthenia gravis, ROC: Receiver operating characteristic, DCA: Decision curve analysis. (For interpretation of the references to color in this figure legend, the reader is referred to the Web version of this article.)

To enhance diagnostic and predictive capabilities, a logistic regression analysis was conducted on C3AR1, MID1IP1, and PIK3IP1, which exhibited similar expression trends. After further screening, two key core genes, MID1IP1 and PIK3IP1, were identified. Based on these genes, a nomogram was constructed (Fig. 7A). The calibration curve demonstrated that the predicted probabilities of the constructed nomogram diagnostic model were nearly identical to the ideal model (Fig. 7B). ROC curve analysis was employed to evaluate the area under the curve (AUC) values of each core gene and the nomogram, determining their sensitivity and specificity in diagnosing MG-associated DCM. As expected, both core genes exhibited AUC values above 0.82, while the nomogram displayed a higher AUC value compared to each core gene, indicating its strong diagnostic value for MG-associated DCM (Fig. 7C). Additionally, decision curve analysis (DCA) was performed to further analyze the graphical model, revealing that decision-making based on the nomogram may assist in diagnosing MG-associated DCM (Fig. 7D).

To validate the nomogram, an external validation set from the GEO database, the GSE29819 dataset containing DCM patients, was utilized. ROC curve analysis, DCA, and calibration curve analysis of this nomogram all indicated favorable diagnostic performance for MG-associated DCM patients (Fig. 7E–G).

### 3.9 Single gene set enrichment analysis

**Fig. 8:**
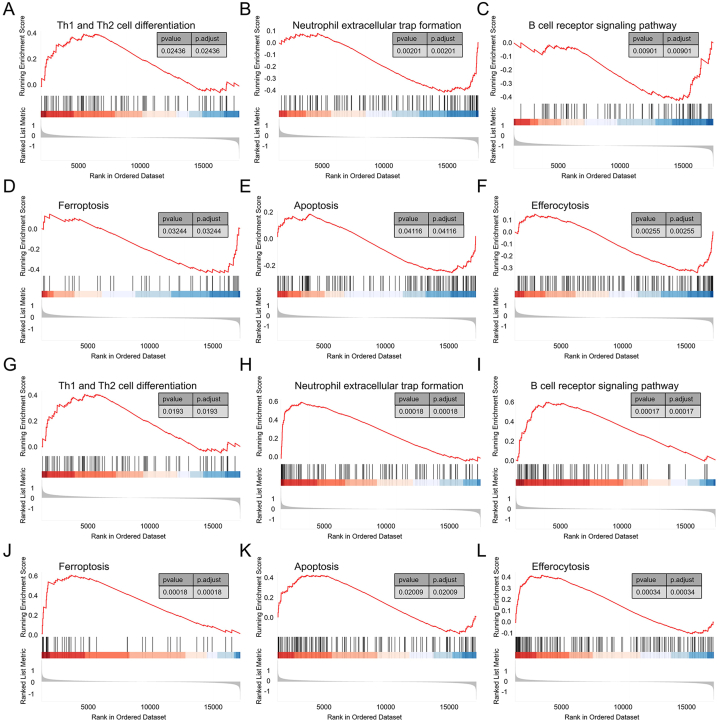
Single gene GSEA. A–F: GSEA results of PIK3IP1 in the DCM dataset GSE57338. G–L: GSEA results of MID1IP1 in the DCM dataset GSE57338. GSEA: Gene set enrichment analysis, DCM: Dilated cardiomyopathy.

GSEA analysis performed on the DCM dataset identified 74 pathways, including "Th1 and Th2 cell differentiation," "Neutrophil extracellular trap formation," "B cell receptor signaling pathway," "Ferroptosis," "Apoptosis," "Efferocytosis," and "Cellular senescence," as regulatory targets of PIK3IP1 (Fig. 8A–F). Similarly, in the single gene GSEA analysis of MID1IP1, 89 pathways were identified as regulatory targets, which also included "Th1 and Th2 cell differentiation," "Neutrophil extracellular trap formation," "B cell receptor signaling pathway," "Ferroptosis," "Apoptosis," "Efferocytosis," and "Cellular senescence" (Fig. 8G-L).

### 3.10 Analysis of immune cell infiltration in DCM and MG and the association with invasive immune cells

**Fig. 9:**
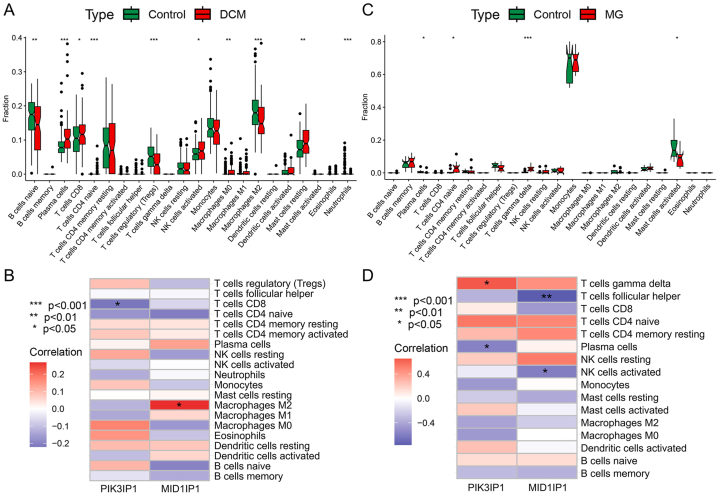
Analysis of immune cell infiltration in DCM and MG. A: Box plot comparing the infiltration of 22 immune cell types between the DCM group and the control group. B: Association between differentially infiltrated immune cells in DCM and the two hub genes, at a threshold of *p* < 0.05. C: Box plot comparing the infiltration of 22 immune cell types between the MG group and the control group. D: Association between differentially infiltrated immune cells in MG and the two hub genes, at a threshold of *p* < 0.05. DCM: Dilated cardiomyopathy, MG: Myasthenia gravis.

Functional and pathway analyses of DCM and MG-related pathogenic genes revealed a close association with inflammation and immune processes. To investigate immune cell infiltration and its correlation with immune regulation and diagnostic biomarkers in DCM and MG, we employed the CIBERSORT algorithm to infer the characteristics of immune cells. Fig. 9A displays the proportions of 22 immune cell types in DCM and normal samples from the GSE57338 dataset, highlighting significant differences between DCM and normal samples across 10 immune cell subtypes. Compared to the control group, DCM exhibited higher proportions of Plasma cells, T cells CD8, T cells CD4 naive, NK cells activated, Macrophages M0, and Mast cells resting, while the proportions of B cells naive, T cells regulatory (Tregs), Macrophages M2, and Neutrophils were lower. Furthermore, we further explored the association between the expression of two key genes and the proportions of different infiltrating immune cell types. As shown in Fig. 9B, the expression of the key genes MID1IP1 and PIK3IP1 was significantly correlated with immune cell infiltration in DCM. Fig. 9C displays the proportions of 22 immune cell types in MG and normal samples from the GSE85452 dataset, showcasing significant differences between MG and normal samples across 4 immune cell subtypes. Compared to the control group, MG exhibited higher proportions of T cells CD4 naive and T cells gamma delta, while the proportions of Mast cells activated and Plasma cells were lower. In the further exploration of the association between the expression of the two key genes and the proportions of different infiltrating immune cell types, it was found that the key genes MID1IP1 and PIK3IP1 were significantly correlated with immune cell infiltration in MG (Fig. 9D).

## 4 Discussion

The occurrence rate of MG combined with DCM is relatively low. However, once it occurs, the symptoms are often significantly aggravated. In addition to manifesting as progressive muscle weakness and easy fatigue, the involvement of the myocardium also leads to notable cardiovascular symptoms such as palpitations and shortness of breath [13]. Furthermore, the presence of MG-associated DCM cannot be overlooked as it significantly affects patients' quality of life. Currently, the mechanisms underlying the development of MG-associated DCM are not fully understood, and there is significant confusion in its diagnosis. This study provides an in-depth analysis of MG-associated DCM by comprehensively utilizing bioinformatics tools and analytical methods. It explores the potential association and possible co-mechanisms between MG and DCM while investigating candidate biomarkers for diagnosing MG with DCM.

In this study, key gene modules were identified and differential gene expression analysis was conducted using expression profile matrices of MG and DCM. A total of 11 differentially expressed genes that were commonly expressed were selected. GO and KEGG analysis revealed that these differentially expressed genes were mainly enriched in inflammation and immune-related pathways. Immune and inflammatory processes play important regulatory roles in MG-associated DCM.

MG itself is an autoimmune-mediated disease, where the immune system mistakenly recognizes the receptors at the neuromuscular junction as foreign substances. This leads to the production of antibodies and interference with the transmission of nerve impulses, resulting in impaired neuromuscular transmission function [14]. This pathological process is mainly caused by the production of anti-acetylcholine receptor antibodies, which subsequently reduce the sensitivity of muscles to acetylcholine [15]. Malfunction of the autoimmune response often leads to the production of anti-cardiac antibodies, which can bind to antigens on the surface of myocardial cells. This is accompanied by abnormal activation of immune cells such as T cells and B cells, causing myocardial damage [16]. With the involvement of the immune system, the release of inflammatory cells and mediators leads to inflammatory pathological changes in myocardial tissue, including infiltration of inflammatory cells, release of inflammatory mediators, and activation of inflammatory signaling pathways. These inflammatory reactions directly affect the structure and function of myocardial cells, accelerating the degeneration and necrosis of myocardial fibers, ultimately resulting in weakened cardiac contraction [17]. Post-mortem examination reports have revealed a significant infiltration of lymphocytes in MG patients, along with extensive giant cell infiltration in the heart and skeletal muscles. Additionally, there is evidence of muscle fiber necrosis and structural abnormalities [4]. Abnormal activity of immune cells and inflammatory infiltration may be an important mechanism underlying the co-occurrence of MG and DCM.

Further analysis of immune infiltration has revealed that both DCM and MG samples exhibit changes in the proportions of various immune cell subtypes compared to the control group. Both conditions show a higher proportion of CD4 T cells, which may reflect an active state of inflammation and immune cells, suggesting the significant role of the immune system in the pathogenesis of MG-associated DCM. These findings support the importance of the immune system in the pathophysiology of MG-associated DCM.

Furthermore, through machine learning and various algorithm evaluations, we have identified that the genes MID1IP1 and PIK3IP1 may play important roles in the pathogenesis of the disease. MID1IP1, a protein that interacts with the MID1 (Midline 1) protein, is involved in various biological processes [18]. MID1IP1 exhibits significant functionality in cell cycle regulation, apoptosis, lipid metabolism, and other biological processes [19,20]. Studies have indicated that MID1IP1 may be involved in regulating the stability and subcellular localization of important intracellular proteins, thus influencing the biological behavior of cells [21]. Additionally, MID1IP1 is also believed to be closely associated with the occurrence and progression of certain diseases, as its abnormal expression may lead to cellular functional imbalances, thereby affecting the pathogenesis of diseases. On the other hand, PIK3IP1 is a key regulatory factor in the PI3K signaling pathway. By interacting with the p110α subunit of PI3K, PIK3IP1 can inhibit the activity of the PI3K signaling pathway, thereby influencing biological processes such as cell proliferation, survival, and migration [22]. In the field of autoimmune diseases, PIK3IP1 is considered a key regulatory factor. This signaling plays a crucial role in the activation and regulation of immune cells, directly impacting their proliferation, differentiation, and activation state [23]. Research has also shown that by modulating the activity of the PI3K signaling pathway, PIK3IP1 affects biological processes such as proliferation, survival, and apoptosis in myocardial cells. Therefore, PIK3IP1 plays a regulatory role in cardiac protection and the occurrence and progression of cardiovascular diseases [24,25].

MID1IP1 and PIK3IP1, as important regulatory factors, play significant roles in cellular biological processes as well as the occurrence and development of various diseases. Further research and exploration are needed to uncover the specific functions and regulatory mechanisms of these factors in MG-associated DCM. They hold the potential to become crucial targets for the diagnosis and treatment of MG-associated DCM in the future. Based on this, we have constructed a nomogram and validated it using an external dataset. ROC curve analysis, decision curve analysis, and calibration curve analysis all demonstrate that this nomogram exhibits significant diagnostic performance for patients with MG-related DCM.

In the treatment of MG-associated DCM, we have utilized the CMAP database to screen a series of small molecule drugs with potential therapeutic effects. Among them, SB-216763 is a selective inhibitor of Glycogen Synthase Kinase-3 (GSK-3). Its mechanism of action primarily involves competitively binding to the ATP-binding site GSK-3, thereby inhibiting its activity [26]. GSK-3 is an important serine/threonine kinase that plays a crucial role in cellular signal transduction, glycogen metabolism, and cell cycle regulation, among other biological processes [27]. Research has shown that SB-216763 has protective effects in conditions such as cardiac and renal injury and cardiac reperfusion [26,28]. Furthermore, huperzine-A is a natural acetylcholinesterase inhibitor primarily found in certain plants, particularly *huperzia serrata.* The mechanism of action of huperzine A primarily involves its inhibitory effect on acetylcholinesterase (AChE), an enzyme involved in the degradation of acetylcholine. By inhibiting AChE, huperzine-A increases the concentration of acetylcholine in the nervous system, thereby enhancing neurotransmitter transmission efficiency [29]. Additionally, Huperzine A may exert a positive impact on neuronal protection and function maintenance through mechanisms such as modulating the glutamate system and providing neuroprotection to nerve cells. Its potential in protecting the nervous system and improving neural conduction function is evident [30]. These medications hold promising potential in the treatment of MG-related DCM, offering new avenues for future therapeutic strategies.

In conclusion, this study comprehensively explores the potential mechanisms underlying MG-related DCM through multifaceted analysis. It highlights the crucial roles of immunity and inflammation in the development of MG-related DCM and introduces a diagnostic flowchart based on MID1IP1/PIK3IP1. These findings provide important scientific evidence for future clinical practice.

## 5 Limitations of the study

Although these findings provide important scientific evidence for future clinical practice, but further experimental validation and clinical research are still necessary to confirm these discoveries and provide a more reliable scientific basis for clinical practice. Additionally, more in-depth research and exploration are required to develop effective treatment strategies and preventive measures for MG-related DCM.

## Funding

This study was supported by The First Affiliated Hospital of Guangzhou University of Traditional Chinese Medicine 2023 Hospital Young and Middle-aged Key Talent Cultivation Project [grant numbers GZYYYRCXM2023]; Suzhou Industrial Park Oriental Huaxia Cardiovascular Health Research Institute: Li·Xin Traditional Chinese Medicine Research and Innovation Fund [grant numbers: 2023A0755]; Guangzhou Science and Technology Bureau Municipal University Joint Project [grant numbers 2060206]; Guangzhou Science and Technology Plan Project: Service Platform for the Deep Integration of Production, Learning, and Research of Famous and Excellent Chinese Patent Drugs [grant numbers 20212210006].

## Consent for publication

The manuscript has been approved by all authors for publication.

## Data availability statement

RNA-seq data that support the findings of this study have been deposited in the Gene Expression Omnibus (10.13039/100000085GEO) under accession codes GSE57338, GSE29819, and GSE85452. All other data supporting the findings of this study are available from the corresponding authors on reasonable request.

## CRediT authorship contribution statement

**Guiting Zhou:** Writing – original draft, Visualization, Software. **Shushu Wang:** Writing – original draft. **Liwen Lin:** Writing – original draft, Methodology. **Kachun Lu:** Writing – original draft. **Zhichao Lin:** Software. **Ziyan Zhang:** Visualization. **Yuling Zhang:** Writing – original draft. **Danling Cheng:** Software. **KaMan Szeto:** Writing – original draft. **Rui Peng:** Writing – review & editing, Conceptualization. **Chuanjin Luo:** Writing – review & editing, Funding acquisition, Conceptualization.

## Declaration of competing interest

The authors declare that they have no known competing financial interests or personal relationships that could have appeared to influence the work reported in this paper.

## Abbreviations

DCM: Dilated cardiomyopathy
MG: Myasthenia gravis
GEO: Gene Expression Omnibus
WGCNA: Weighted gene co-expression network analysis
PPI: Protein-protein interaction
GO: Gene ontology
KEGG: Kyoto Encyclopedia of Genes and Genomes
GSEA: Gene set enrichment analysis
CMAP: Connectivity map
MID1IP1: Midline 1 interacting protein1
PIK3IP1: PI3K-interacting protein 1
MAD: Median absolute deviation
DEG: Differentially expressed gene
CG: Common gene
LASSO: Least absolute shrinkage and selection operator
RF: Random forest
ROC: Receiver operating characteristic
DCA: Decision curve analysis
BP: Biological processes
CC: Cellular components
MF: Molecular function
AUC: Area under the curve
GSK-3: Glycogen synthase kinase-3
AChE: Acetylcholinesterase

## References

[1] Querol L., Illa I. (2013). Myasthenia gravis and the neuromuscular junction. Curr. Opin. Neurol..

[2] Japp A.G. (2016). The diagnosis and evaluation of dilated cardiomyopathy. J. Am. Coll. Cardiol..

[3] Gayfield S., Busken J., Mansur S. (2022). A case report and 31-case study: Does Takotsubo cardiomyopathy in myasthenia gravis patients have a high Mortality rate?. Cureus.

[4] Namba T., Brunner N.G., Grob D. (1974). Idiopathic giant cell polymyositis. Report of a case and review of the syndrome. Arch. Neurol..

[5] de Jongste M.J., Oosterhuis H.J., Lie K.I. (1986). Intractable ventricular tachycardia in a patient with giant cell myocarditis, thymoma and myasthenia gravis. Int. J. Cardiol..

[6] Suzuki S. (2009). Autoimmune targets of heart and skeletal muscles in myasthenia gravis. Arch. Neurol..

[7] Cooper L.T. (2007). The role of endomyocardial biopsy in the management of cardiovascular disease - a scientific statement from the American Heart Association, the American College of Cardiology, and the European Society of Cardiology. J. Am. Coll. Cardiol..

[8] Suzuki S., Utsugisawa K., Suzuki N. (2013). Overlooked non-motor symptoms in myasthenia gravis. J. Neurol. Neurosurg. Psychiatry.

[9] Antonacci Y. (2019). Annual International Conference of the IEEE Engineering in Medicine and Biology Society. 2019.

[10] Savargiv M., Masoumi B., Keyvanpour M.R. (2021). A new random forest algorithm based on learning Automata. Comput. Intell. Neurosci..

[11] Walker A.M. (2022). Evaluating the performance of random forest and iterative random forest based methods when applied to gene expression data. Comput. Struct. Biotechnol. J..

[12] Subramanian A. (2005). Gene set enrichment analysis: a knowledge-based approach for interpreting genome-wide expression profiles. Proc. Natl. Acad. Sci. U.S.A..

[13] Evoli A. (2002). Thymoma in patients with MG - characteristics and long-term outcome. Neurology.

[14] Gilhus N.E. (2019). Myasthenia gravis. Nat. Rev. Dis. Prim..

[15] Verschuuren J.J.G.M. (2013). Pathophysiology of myasthenia gravis with antibodies to the acetylcholine receptor, muscle-specific kinase and low-density lipoprotein receptor-related protein 4. Autoimmun. Rev..

[16] Schultheiss H.-P. (2019). Dilated cardiomyopathy. Nat. Rev. Dis. Prim..

[17] Hua X. (2020). Single-cell RNA sequencing to dissect the immunological network of autoimmune myocarditis. Circulation.

[18] Aipoalani D.L. (2010). Overlapping roles of the glucose-responsive genes, S14 and S14R, in hepatic lipogenesis. Endocrinology.

[19] Jung J.H. (2020). Colocalization of MID1IP1 and c-Myc is critically involved in liver cancer growth via regulation of ribosomal protein L5 and L11 and CNOT2. Cells.

[20] Chiu Y.-T. (2023). Mid-line 1 interacting protein 1 promotes cancer metastasis via FOS like 1-mediated matrix metalloproteinase 9 signaling in HCC. Hepatology.

[21] Zhou X. (2018). Mid1ip1b modulates apical reorientation of non-centrosomal microtubule organizing center in epithelial cells. Journal of Genetics and Genomics.

[22] Zhu Z. (2007). PI3K is negatively regulated by PIK3IP1, a novel p110 interacting protein. Biochem. Biophys. Res. Commun..

[23] Xie W. (2022). Regulation of autoimmune disease progression by Pik3ip1 through metabolic reprogramming in T cells and therapeutic implications. Sci. Adv..

[24] Liu X. (2022). Knockdown of forkhead box protein P1 alleviates hypoxia reoxygenation injury in H9c2 cells through regulating Pik3ip1/Akt/eNOS and ROS/mPTP pathway. Bioengineered.

[25] Song H.K. (2015). Pik3ip1 modulates cardiac hypertrophy by inhibiting PI3K pathway. PLoS One.

[26] Zhang Y.-D. (2018). SB-216763, a GSK-3 inhibitor, protects against aldosterone-induced cardiac, and renal injury by activating autophagy. J. Cell. Biochem..

[27] Hernandez F. (2009). GSK3 inhibitors and disease. Mini-Rev. Med. Chem..

[28] Gross E.R., Hsu A.K., Gross G.J. (2008). Delayed cardioprotection afforded by the glycogen synthase kinase 3 inhibitor SB-216763 occurs via a KATP- and MPTP-dependent mechanism at reperfusion. Am. J. Physiol. Heart Circ. Physiol..

[29] Li J. (2008). Huperzine a for Alzheimer's disease. Cochrane Database Syst. Rev..

[30] Mao X.-Y. (2016). Huperzine A alleviates oxidative glutamate toxicity in hippocampal HT22 cells via activating BDNF/TrkB-dependent PI3K/Akt/mTOR signaling pathway. Cell. Mol. Neurobiol..

