# Supplementary material for: Screening for immune-related biomarkers associated with myasthenia gravis and dilated cardiomyopathy based on bioinformatics analysis and machine learning
Source: Heliyon. 2024 Mar 20;10(7):e28446. doi: 10.1016/j.heliyon.2024.e28446 (PMC10988011; doi:10.1016/j.heliyon.2024.e28446)
Supplement: Multimedia component 1 [file mmc1.docx]

Table 1 DEGs related to MG in GSE85452

| NO. | id | logFC | AveExpr | t | P.Value | adj.P.Val | B |
| --- | --- | --- | --- | --- | --- | --- | --- |
| 1 | ZNF296 | 0.861437259 | 8.599490251 | 5.261095317 | 1.59E-05 | 0.05134798 | 2.919143599 |
| 2 | ERICH1 | -0.55625107 | 8.933433714 | -5.257249582 | 1.61E-05 | 0.05134798 | 2.91029975 |
| 3 | CLP1 | 0.634848008 | 6.984276254 | 5.012607412 | 3.08E-05 | 0.05134798 | 2.345772958 |
| 4 | SLC30A1 | 0.585913144 | 7.974546568 | 4.991903313 | 3.26E-05 | 0.05134798 | 2.297845961 |
| 5 | CHIC2 | -0.521749612 | 8.780700362 | -4.889353224 | 4.28E-05 | 0.05134798 | 2.060189277 |
| 6 | MAD2L1BP | 0.510389848 | 7.089400541 | 4.868787498 | 4.52E-05 | 0.05134798 | 2.012481731 |
| 7 | YOD1 | 0.649284248 | 7.471997419 | 4.807098723 | 5.33E-05 | 0.05134798 | 1.869301662 |
| 8 | DUSP12 | 0.614677935 | 8.328216286 | 4.51934461 | 0.000114786 | 0.078442895 | 1.200640606 |
| 9 | MX2 | -0.801422059 | 8.943665139 | -4.42860253 | 0.000146121 | 0.078442895 | 0.989835905 |
| 10 | ING1 | 0.757473574 | 8.033388642 | 4.423952654 | 0.000147939 | 0.078442895 | 0.979038236 |
| 11 | PTPRE | -0.52170492 | 10.446069 | -4.291170697 | 0.000210461 | 0.078442895 | 0.670987738 |
| 12 | IRS2 | -0.665842802 | 9.310149606 | -4.289340592 | 0.000211484 | 0.078442895 | 0.666746685 |
| 13 | FEM1B | 0.507918924 | 6.930546027 | 4.255004793 | 0.00023163 | 0.078442895 | 0.587206778 |
| 14 | LOC727762 | 0.537235686 | 6.920051003 | 4.244149683 | 0.000238386 | 0.078442895 | 0.562072746 |
| 15 | TMEM2 | -0.546469762 | 7.744347467 | -4.213318025 | 0.000258657 | 0.078442895 | 0.490719214 |
| 16 | HHEX | 0.637789649 | 8.833191821 | 4.137918182 | 0.000315704 | 0.080006018 | 0.316461358 |
| 17 | SERPINB2 | -1.176359522 | 7.40775361 | -4.10777591 | 0.000341843 | 0.080291472 | 0.246906025 |
| 18 | NCOA7 | -0.579098139 | 8.636826021 | -4.088053559 | 0.000360092 | 0.080643818 | 0.201431859 |
| 19 | TIGA1 | 0.515954326 | 8.756393286 | 4.010526597 | 0.00044162 | 0.089632816 | 0.022983713 |
| 20 | PPP1R2 | 0.748364681 | 8.996300547 | 3.96036895 | 0.000503798 | 0.097031456 | -0.092178648 |
| 21 | YWHAG | -0.572357526 | 7.62980476 | -3.907135529 | 0.00057922 | 0.100564307 | -0.214125646 |
| 22 | C22orf32 | 0.519460476 | 7.181461738 | 3.881351469 | 0.000619639 | 0.100564307 | -0.273081028 |
| 23 | FPR2 | -0.874094958 | 7.797785115 | -3.872974395 | 0.000633358 | 0.100564307 | -0.292218932 |
| 24 | DOCK10 | -0.511179243 | 8.976104677 | -3.864975992 | 0.000646735 | 0.100564307 | -0.310484098 |
| 25 | ZDHHC1 | 0.57543197 | 8.208301974 | 3.858425089 | 0.000657897 | 0.100564307 | -0.325438122 |
| 26 | C3AR1 | -0.808471817 | 7.600022038 | -3.850244613 | 0.000672102 | 0.100869525 | -0.344104838 |
| 27 | HSPA1A | -1.191601933 | 9.396888242 | -3.845294982 | 0.000680843 | 0.100869525 | -0.355395285 |
| 28 | EGR2 | -0.890811443 | 9.05856973 | -3.798144474 | 0.000769901 | 0.10847221 | -0.462795754 |
| 29 | KLF11 | 0.631900097 | 8.026818744 | 3.778259851 | 0.000810797 | 0.110643093 | -0.508003244 |
| 30 | PTGS2 | -1.486527065 | 8.987466813 | -3.748488456 | 0.000876033 | 0.114002705 | -0.575588031 |
| 31 | OBFC2A | -0.552603943 | 7.959630653 | -3.735913786 | 0.000905107 | 0.114969402 | -0.604096932 |
| 32 | LFNG | -0.563725301 | 9.31606708 | -3.714560009 | 0.000956656 | 0.118374034 | -0.65245745 |
| 33 | SON | 0.535421758 | 8.817545094 | 3.687651338 | 0.001025718 | 0.123470783 | -0.713302264 |
| 34 | WASPIP | -0.591182205 | 9.104574354 | -3.68136568 | 0.001042539 | 0.123833198 | -0.72749933 |
| 35 | SERTAD2 | 0.539182142 | 9.372868034 | 3.633183862 | 0.001180745 | 0.132216009 | -0.836118794 |
| 36 | PIK3IP1 | 0.564873064 | 8.005781683 | 3.600062302 | 0.00128596 | 0.132749234 | -0.910567291 |
| 37 | OAS1 | -0.744184697 | 7.399228294 | -3.590035621 | 0.001319576 | 0.132749234 | -0.93306796 |
| 38 | PRIC285 | -0.500190933 | 8.418876522 | -3.574593603 | 0.001373028 | 0.133558211 | -0.967686941 |
| 39 | BCL11A | 0.519645393 | 6.611597411 | 3.504330495 | 0.001644064 | 0.147965742 | -1.124664547 |
| 40 | IL1B | -0.980205494 | 10.92099877 | -3.482259675 | 0.001739461 | 0.148802834 | -1.173782172 |
| 41 | C20orf11 | 0.500734109 | 8.940011074 | 3.436527909 | 0.001954539 | 0.160873565 | -1.275248939 |
| 42 | DYNLL1 | -0.949221326 | 9.048258634 | -3.419425439 | 0.002041423 | 0.163321581 | -1.313084975 |
| 43 | PREP | -0.561272021 | 7.441121866 | -3.402004115 | 0.00213377 | 0.163321581 | -1.351563134 |
| 44 | RNASEL | -0.534229808 | 7.32949484 | -3.377152985 | 0.002272535 | 0.163321581 | -1.406338489 |
| 45 | VPS35 | -0.584399517 | 8.085571614 | -3.367274474 | 0.002330087 | 0.163321581 | -1.428074563 |
| 46 | CIRBP | 0.548170613 | 9.736819066 | 3.364683471 | 0.002345414 | 0.163321581 | -1.433772067 |
| 47 | MOBKL1B | -0.528375033 | 8.348816253 | -3.363542425 | 0.002352195 | 0.163321581 | -1.436280704 |
| 48 | DNCL1 | -1.001670465 | 10.21256985 | -3.361105419 | 0.002366741 | 0.163321581 | -1.441637589 |
| 49 | CDCA4 | 0.5265277 | 7.288565707 | 3.347489649 | 0.002449623 | 0.163321581 | -1.471542446 |
| 50 | RNF149 | -0.514025228 | 10.22618701 | -3.341966763 | 0.002484035 | 0.163321581 | -1.483660629 |
| 51 | NAGK | -0.724053271 | 10.03065938 | -3.328519676 | 0.002569788 | 0.163321581 | -1.51313673 |
| 52 | MYOF | -0.585966517 | 8.117822728 | -3.321538423 | 0.002615428 | 0.163321581 | -1.52842323 |
| 53 | RUNX3 | 0.671712973 | 9.190277216 | 3.311914943 | 0.002679627 | 0.163321581 | -1.549476667 |
| 54 | RAB12 | 0.662923895 | 7.504996315 | 3.251154721 | 0.00312148 | 0.172757786 | -1.681891574 |
| 55 | SNHG9 | 0.624500008 | 8.253804264 | 3.216722875 | 0.003402201 | 0.178060868 | -1.756523678 |
| 56 | C16orf87 | 0.535483668 | 7.749683324 | 3.159498859 | 0.003923217 | 0.188902879 | -1.879878043 |
| 57 | CHMP1B | -0.556873134 | 10.43367303 | -3.147605407 | 0.004040729 | 0.191685836 | -1.905405946 |
| 58 | FRAT2 | -0.635140012 | 9.50438275 | -3.129793793 | 0.004223049 | 0.194643908 | -1.943563756 |
| 59 | EGR3 | -0.774182289 | 6.99983169 | -3.099378384 | 0.004552742 | 0.199737608 | -2.008517384 |
| 60 | CX3CR1 | -0.680181904 | 8.025567757 | -3.056455526 | 0.005060169 | 0.202846781 | -2.099728849 |
| 61 | LEPROTL1 | -0.535552611 | 8.094271466 | -3.021505777 | 0.005512802 | 0.202846781 | -2.173593872 |
| 62 | VNN2 | -0.508220198 | 10.096564 | -3.021123743 | 0.005517956 | 0.202846781 | -2.174399242 |
| 63 | SIK1 | -0.704919394 | 8.565475435 | -2.980587249 | 0.006091804 | 0.205838853 | -2.259597829 |
| 64 | EPSTI1 | -0.723777999 | 8.979793794 | -2.964728769 | 0.006331405 | 0.207836581 | -2.292788181 |
| 65 | GIMAP8 | -1.004443449 | 8.379678923 | -2.954146706 | 0.006496242 | 0.207836581 | -2.314890678 |
| 66 | CYBB | -1.145674834 | 9.679914726 | -2.94819607 | 0.006590719 | 0.208524529 | -2.32730374 |
| 67 | CLEC2B | 0.622776959 | 7.442066269 | 2.945687385 | 0.006630938 | 0.208524529 | -2.332533427 |
| 68 | TAOK1 | -0.652078208 | 7.169725572 | -2.924482115 | 0.00698034 | 0.210450061 | -2.376656356 |
| 69 | SMAD7 | 0.534391473 | 6.971887456 | 2.913389819 | 0.00717 | 0.210450061 | -2.39967753 |
| 70 | OAS2 | -0.78847343 | 9.40294589 | -2.909780055 | 0.007232768 | 0.210450061 | -2.407160461 |
| 71 | COPB2 | -0.515702316 | 8.486101782 | -2.897797468 | 0.007444882 | 0.210530529 | -2.431968639 |
| 72 | ISG15 | -0.615403567 | 8.298872115 | -2.878421603 | 0.007800387 | 0.210530529 | -2.47198059 |
| 73 | CEPT1 | -0.631667378 | 8.13412588 | -2.869121335 | 0.00797668 | 0.210530529 | -2.491140373 |
| 74 | MID1IP1 | -0.625303293 | 7.987276658 | -2.865179925 | 0.008052524 | 0.210530529 | -2.49925119 |
| 75 | TLR5 | -0.610350626 | 7.940593254 | -2.85170893 | 0.008316925 | 0.210530529 | -2.526931666 |
| 76 | ANKDD1A | 0.533499735 | 6.771720992 | 2.849545917 | 0.008360137 | 0.210530529 | -2.531370363 |
| 77 | TBC1D9 | -0.563687938 | 8.168940749 | -2.849448267 | 0.008362093 | 0.210530529 | -2.531570712 |
| 78 | ANPEP | -0.603816738 | 8.090315246 | -2.843202381 | 0.008488092 | 0.210530529 | -2.544378393 |
| 79 | SAMD9 | -0.523367852 | 7.824144634 | -2.839532181 | 0.008562966 | 0.210530529 | -2.551898017 |
| 80 | RNF10 | 0.510930161 | 7.879196754 | 2.839397167 | 0.008565732 | 0.210530529 | -2.552174547 |
| 81 | NLRP12 | -0.61920327 | 8.421987453 | -2.839194688 | 0.008569882 | 0.210530529 | -2.552589243 |
| 82 | CXCL2 | -1.031409187 | 7.689814096 | -2.817423848 | 0.009027349 | 0.213834484 | -2.597093055 |
| 83 | EXOSC3 | -0.506603557 | 7.64921219 | -2.808920732 | 0.009212203 | 0.213834484 | -2.614428957 |
| 84 | MX1 | -0.71276705 | 10.42302163 | -2.793852965 | 0.009548567 | 0.213834484 | -2.645084326 |
| 85 | PCMT1 | -0.573258691 | 9.629525661 | -2.787892633 | 0.00968479 | 0.213834484 | -2.657187747 |
| 86 | PRDM1 | -0.732364265 | 6.933558142 | -2.780364413 | 0.009859462 | 0.213897361 | -2.67245636 |
| 87 | CXXC5 | 0.530067022 | 7.719511325 | 2.7789623 | 0.00989232 | 0.213897361 | -2.675297791 |
| 88 | SAMD9L | -0.693189734 | 8.063131875 | -2.76709306 | 0.010174632 | 0.215901399 | -2.699322034 |
| 89 | RGS18 | -0.610196412 | 9.412785702 | -2.754984279 | 0.010470439 | 0.215910775 | -2.72377697 |
| 90 | DPYD | -0.635992675 | 9.221972222 | -2.740589674 | 0.010832603 | 0.21875797 | -2.752776537 |
| 91 | WDR40A | -0.562926703 | 8.178316208 | -2.740469497 | 0.010835675 | 0.21875797 | -2.753018316 |
| 92 | ST6GAL1 | -0.559033751 | 8.31582873 | -2.72789287 | 0.011161784 | 0.220950336 | -2.778290319 |
| 93 | RHOB | -0.586989085 | 10.1730993 | -2.727439539 | 0.011173709 | 0.220950336 | -2.779200133 |
| 94 | E2F2 | 0.709937031 | 8.013873499 | 2.683210999 | 0.012396351 | 0.222152598 | -2.867581362 |
| 95 | GIMAP1 | -0.56182008 | 8.181400275 | -2.682607208 | 0.012413877 | 0.222152598 | -2.868782603 |
| 96 | MCL1 | -0.575501351 | 8.286017014 | -2.672116165 | 0.012722133 | 0.224334931 | -2.889631412 |
| 97 | LOC650557 | 1.131029573 | 7.322979125 | 2.650740611 | 0.013372481 | 0.225528884 | -2.931974723 |
| 98 | C9orf89 | 0.575928676 | 8.217915282 | 2.625332581 | 0.014185874 | 0.228554054 | -2.98206516 |
| 99 | IFI44L | -0.797395704 | 8.257314667 | -2.624356782 | 0.014218014 | 0.228554054 | -2.983983617 |
| 100 | XYLT1 | 0.668544084 | 7.045710227 | 2.586257208 | 0.015527262 | 0.236535046 | -3.058579301 |
| 101 | CREB1 | 0.657621779 | 7.939345395 | 2.584994796 | 0.015572505 | 0.236535046 | -3.061040573 |
| 102 | FAR1 | -0.508505223 | 8.146127547 | -2.5812837 | 0.015706216 | 0.236818698 | -3.068272033 |
| 103 | GCA | -0.527810585 | 10.13752334 | -2.57781025 | 0.015832331 | 0.237192843 | -3.075035122 |
| 104 | RAB10 | -0.622135556 | 10.21831902 | -2.548572811 | 0.016931788 | 0.241616939 | -3.131758266 |
| 105 | SERTAD3 | 0.520228805 | 7.439631835 | 2.548347449 | 0.016940532 | 0.241616939 | -3.132194056 |
| 106 | ID1 | 0.601183965 | 7.793085402 | 2.544077314 | 0.017106991 | 0.241616939 | -3.140447222 |
| 107 | OAS3 | -0.502675841 | 8.59513273 | -2.538651526 | 0.017320688 | 0.241616939 | -3.150922555 |
| 108 | NACC2 | 0.580711637 | 8.350782451 | 2.536199082 | 0.017418088 | 0.241616939 | -3.155653169 |
| 109 | ELF4 | 0.504211803 | 8.910690058 | 2.528404687 | 0.017731025 | 0.24219826 | -3.17067061 |
| 110 | GAPT | -0.869017957 | 9.253290582 | -2.523556198 | 0.017928306 | 0.243455308 | -3.179998729 |
| 111 | LOC100133583 | 0.701651441 | 8.895617733 | 2.522570973 | 0.017968641 | 0.243455308 | -3.181892962 |
| 112 | LPXN | -0.582658186 | 9.790571206 | -2.51200727 | 0.018406433 | 0.24493755 | -3.202176201 |
| 113 | DUSP2 | -0.621235464 | 8.667899475 | -2.506743831 | 0.01862823 | 0.24602086 | -3.212264003 |
| 114 | PDE4B | -0.677335335 | 7.124906939 | -2.503690724 | 0.018758015 | 0.24602086 | -3.218109875 |
| 115 | STK38 | -0.566532512 | 9.27724435 | -2.498174259 | 0.018994636 | 0.246221012 | -3.228661857 |
| 116 | TMEM176B | -0.51031556 | 6.758144226 | -2.487699097 | 0.019451562 | 0.246221012 | -3.248661376 |
| 117 | MNDA | -0.674666063 | 10.40218106 | -2.482892616 | 0.019664598 | 0.246575625 | -3.257821532 |
| 118 | FAM117B | -0.511979881 | 8.839404392 | -2.47723061 | 0.019918315 | 0.248141497 | -3.26859874 |
| 119 | ACTR3 | -0.662085733 | 9.459588822 | -2.476499262 | 0.019951307 | 0.248231375 | -3.269989747 |
| 120 | GPR1 | 0.516192848 | 7.508079778 | 2.455312225 | 0.020929218 | 0.253201466 | -3.310181159 |
| 121 | C17orf91 | 0.518975581 | 9.206513106 | 2.420856755 | 0.022614216 | 0.258065201 | -3.375100434 |
| 122 | HCP5 | -0.566208847 | 9.30920773 | -2.414814056 | 0.022922267 | 0.258310015 | -3.386428634 |
| 123 | NR4A2 | -0.707004433 | 9.644153605 | -2.414443063 | 0.022941305 | 0.258310015 | -3.387123573 |
| 124 | CD52 | -0.684128267 | 9.713907058 | -2.412209087 | 0.02305625 | 0.258310015 | -3.391306842 |
| 125 | RTN1 | -0.614041605 | 9.174911035 | -2.404354034 | 0.023464628 | 0.259161842 | -3.405997224 |
| 126 | GIMAP7 | -0.914664121 | 8.76111379 | -2.36541328 | 0.025588777 | 0.265607751 | -3.478388477 |
| 127 | XPO1 | -0.512051343 | 8.916715202 | -2.338254914 | 0.02717276 | 0.271445723 | -3.528441212 |
| 128 | DCAF7 | -0.730477475 | 8.779863613 | -2.33157476 | 0.027575841 | 0.272307095 | -3.540697145 |
| 129 | HLA-DQB1 | -0.813796204 | 7.95634643 | -2.321550386 | 0.028190946 | 0.274221017 | -3.559047135 |
| 130 | IFIT1 | -0.708165297 | 7.701270352 | -2.320557243 | 0.028252561 | 0.274313289 | -3.560862399 |
| 131 | CCR1 | -0.567954713 | 8.558640296 | -2.310656306 | 0.028873564 | 0.27615295 | -3.578932363 |
| 132 | GIMAP4 | -0.777466541 | 10.65909567 | -2.309382932 | 0.028954327 | 0.27634308 | -3.581252805 |
| 133 | LOC728059 | -0.70271443 | 8.260667243 | -2.188656937 | 0.037610258 | 0.301320117 | -3.797468414 |
| 134 | DUSP19 | 0.556015894 | 8.313781962 | 2.138995395 | 0.041801918 | 0.309655749 | -3.884167945 |
| 135 | EGR1 | -0.710908864 | 11.23330818 | -2.132305959 | 0.042397402 | 0.310484394 | -3.895743289 |
| 136 | RPPH1 | -0.54666069 | 7.717327134 | -2.119378567 | 0.043569704 | 0.311420964 | -3.918042509 |
| 137 | IFIT2 | -0.529018394 | 8.512129162 | -2.118769759 | 0.04362562 | 0.311420964 | -3.919090389 |
| 138 | HLA-DRB6 | 0.933754655 | 10.58188706 | 2.108039577 | 0.044621698 | 0.312433682 | -3.937525186 |
| 139 | IER5 | 0.528367926 | 9.321658888 | 2.097263245 | 0.045642419 | 0.315079922 | -3.95597434 |
| 140 | MAT2A | -0.597871921 | 9.395744571 | -2.079266375 | 0.047393475 | 0.319000219 | -3.986639029 |
