# Supplementary material for: Screening for immune-related biomarkers associated with myasthenia gravis and dilated cardiomyopathy based on bioinformatics analysis and machine learning
Source: Heliyon. 2024 Mar 20;10(7):e28446. doi: 10.1016/j.heliyon.2024.e28446 (PMC10988011; doi:10.1016/j.heliyon.2024.e28446)
Supplement: Multimedia component 2 [file mmc2.docx]

Table 2 DEGs related to DCM in GSE57338

| NO. | id | logFC | AveExpr | t | P.Value | adj.P.Val | B |
| --- | --- | --- | --- | --- | --- | --- | --- |
| 1 | SERPINA3 | -2.694051077 | 7.533135639 | -21.08101946 | 1.05E-54 | 2.13E-50 | 113.5363901 |
| 2 | FREM1 | 1.076797224 | 5.419434405 | 19.66365413 | 2.38E-50 | 2.41E-46 | 103.6818808 |
| 3 | TUBA3D | -1.023328656 | 7.499334846 | -19.26598532 | 4.11E-49 | 2.77E-45 | 100.8773847 |
| 4 | SMOC2 | 1.276698511 | 6.863183541 | 17.70196015 | 3.50E-44 | 1.57E-40 | 89.69865836 |
| 5 | HMOX2 | -0.635129332 | 8.685876703 | -17.6878182 | 3.88E-44 | 1.57E-40 | 89.59660389 |
| 6 | SLCO4A1 | -1.454134772 | 6.729792586 | -16.68104779 | 6.40E-41 | 2.16E-37 | 82.29402948 |
| 7 | ECM2 | 1.00412955 | 6.007793106 | 16.20435918 | 2.18E-39 | 6.31E-36 | 78.81440844 |
| 8 | PDE5A | 1.105449073 | 6.301202649 | 16.04950787 | 6.88E-39 | 1.74E-35 | 77.68163269 |
| 9 | SFRP4 | 1.761103967 | 4.628917362 | 15.45093245 | 5.88E-37 | 1.32E-33 | 73.29410502 |
| 10 | CD163 | -1.593189055 | 7.678169313 | -15.41582337 | 7.63E-37 | 1.55E-33 | 73.03640433 |
| 11 | SCN2B | 0.96315461 | 5.935006021 | 15.22821574 | 3.08E-36 | 5.67E-33 | 71.65886831 |
| 12 | MNS1 | 1.0295137 | 4.537722508 | 15.21514996 | 3.40E-36 | 5.73E-33 | 71.562903 |
| 13 | GGT5 | -0.832542457 | 7.065218989 | -15.15149469 | 5.46E-36 | 8.50E-33 | 71.09532346 |
| 14 | S1PR3 | -0.653252707 | 6.44677306 | -15.0630915 | 1.05E-35 | 1.52E-32 | 70.44584497 |
| 15 | ZMAT1 | 0.763788572 | 6.625282496 | 14.97763451 | 1.99E-35 | 2.69E-32 | 69.81790521 |
| 16 | FCN3 | -1.576977581 | 8.421833304 | -14.92489251 | 2.95E-35 | 3.73E-32 | 69.43031274 |
| 17 | SDSL | 0.77342228 | 6.350255379 | 14.84692043 | 5.27E-35 | 6.15E-32 | 68.85726013 |
| 18 | NRK | 1.064479813 | 5.130169869 | 14.84182196 | 5.47E-35 | 6.15E-32 | 68.81978744 |
| 19 | CSDC2 | -0.757306428 | 8.827076551 | -14.83482961 | 5.77E-35 | 6.15E-32 | 68.76839489 |
| 20 | FRZB | 1.417102438 | 5.761018991 | 14.76911517 | 9.41E-35 | 9.53E-32 | 68.28539028 |
| 21 | ITIH5 | 0.833237521 | 6.811833174 | 14.68463195 | 1.77E-34 | 1.70E-31 | 67.66440702 |
| 22 | TUBA3E | -0.921457216 | 4.72005007 | -14.50102183 | 6.93E-34 | 6.38E-31 | 66.31479361 |
| 23 | LCN6 | -0.946421114 | 5.795412803 | -14.45141227 | 1.00E-33 | 8.83E-31 | 65.95016135 |
| 24 | LYVE1 | -1.516294486 | 9.335269544 | -14.35784395 | 2.01E-33 | 1.69E-30 | 65.26247914 |
| 25 | FURIN | -0.595973955 | 6.962893989 | -14.35339637 | 2.08E-33 | 1.69E-30 | 65.22979351 |
| 26 | AP3M2 | 0.520764944 | 6.675926209 | 14.32918767 | 2.49E-33 | 1.94E-30 | 65.05188564 |
| 27 | SLC16A9 | 0.906016347 | 4.05735917 | 14.20574584 | 6.25E-33 | 4.59E-30 | 64.1448351 |
| 28 | FNDC1 | 1.018357778 | 5.25985506 | 14.20348252 | 6.35E-33 | 4.59E-30 | 64.12820623 |
| 29 | ASPN | 1.895766158 | 7.244078666 | 14.16362854 | 8.55E-33 | 5.97E-30 | 63.83540912 |
| 30 | C1QTNF7 | 0.610727538 | 3.904290274 | 14.08746481 | 1.51E-32 | 1.02E-29 | 63.27593297 |
| 31 | GPR4 | -0.764350397 | 6.103137143 | -14.06511281 | 1.78E-32 | 1.16E-29 | 63.11176363 |
| 32 | HTRA1 | 0.554101325 | 7.514140059 | 14.04616649 | 2.05E-32 | 1.30E-29 | 62.97261646 |
| 33 | MATN2 | 0.895456208 | 5.73992112 | 14.04142362 | 2.12E-32 | 1.30E-29 | 62.93778467 |
| 34 | LAD1 | -0.634282135 | 5.675434916 | -13.96877597 | 3.64E-32 | 2.17E-29 | 62.40432367 |
| 35 | ISLR | 0.785608451 | 6.082881298 | 13.93888376 | 4.55E-32 | 2.63E-29 | 62.18485915 |
| 36 | KCNN3 | 0.691081593 | 5.899918544 | 13.78196595 | 1.46E-31 | 8.22E-29 | 61.0332023 |
| 37 | LUM | 1.230309238 | 9.42895555 | 13.69835519 | 2.72E-31 | 1.49E-28 | 60.41988338 |
| 38 | BCL6 | -0.755475549 | 8.355519244 | -13.62786182 | 4.59E-31 | 2.39E-28 | 59.90298221 |
| 39 | TLL2 | 0.98483212 | 5.728745235 | 13.5914827 | 6.02E-31 | 2.97E-28 | 59.63630348 |
| 40 | CCND1 | 0.549224898 | 8.58449051 | 13.54370707 | 8.58E-31 | 4.14E-28 | 59.28616458 |
| 41 | PLAGL1 | 0.528418686 | 7.066715969 | 13.48440011 | 1.33E-30 | 6.14E-28 | 58.85165167 |
| 42 | PTN | 0.962566629 | 5.555223485 | 13.4841909 | 1.33E-30 | 6.14E-28 | 58.85011921 |
| 43 | TTC18 | 0.50859016 | 3.953268596 | 13.44525904 | 1.78E-30 | 7.84E-28 | 58.56497148 |
| 44 | ADAMTS4 | -1.342720219 | 6.151949549 | -13.40029226 | 2.49E-30 | 1.05E-27 | 58.23571175 |
| 45 | IFIT2 | 0.67262501 | 5.185625205 | 13.35996114 | 3.35E-30 | 1.33E-27 | 57.9404803 |
| 46 | AQP3 | -1.05424813 | 6.610055944 | -13.24827041 | 7.67E-30 | 2.99E-27 | 57.12332565 |
| 47 | GARNL3 | 0.69467391 | 7.503196142 | 12.98715996 | 5.30E-29 | 1.91E-26 | 55.21582775 |
| 48 | VSIG4 | -1.277712813 | 7.368622738 | -12.98536589 | 5.37E-29 | 1.91E-26 | 55.20273665 |
| 49 | PLCE1 | 0.939247862 | 8.492289301 | 12.91870492 | 8.79E-29 | 2.92E-26 | 54.71647827 |
| 50 | MME | 1.097074925 | 6.279962754 | 12.91266775 | 9.19E-29 | 3.00E-26 | 54.67245562 |
| 51 | ANPEP | -1.155458698 | 5.639592134 | -12.84691393 | 1.49E-28 | 4.66E-26 | 54.19315565 |
| 52 | NPTX2 | -0.873474683 | 5.782772412 | -12.79502744 | 2.19E-28 | 6.72E-26 | 53.81516783 |
| 53 | PLA2G2A | -1.971579038 | 8.306874712 | -12.77727881 | 2.50E-28 | 7.43E-26 | 53.68591857 |
| 54 | FAM46B | -0.849635181 | 6.374160169 | -12.7505994 | 3.04E-28 | 8.67E-26 | 53.49168019 |
| 55 | ALG3 | -0.539240994 | 7.765716324 | -12.70955166 | 4.12E-28 | 1.16E-25 | 53.19294525 |
| 56 | MYH6 | -1.529387486 | 10.43206752 | -12.70773072 | 4.17E-28 | 1.16E-25 | 53.1796961 |
| 57 | CCDC113 | 0.781163495 | 5.48616664 | 12.68167909 | 5.05E-28 | 1.38E-25 | 52.99017446 |
| 58 | TMTC1 | -0.579947367 | 8.571416824 | -12.63659737 | 7.05E-28 | 1.90E-25 | 52.66234611 |
| 59 | LPAR4 | 0.585085927 | 4.612440237 | 12.59623463 | 9.48E-28 | 2.53E-25 | 52.36898125 |
| 60 | PLEKHH2 | 0.931923983 | 5.561076564 | 12.54483868 | 1.38E-27 | 3.60E-25 | 51.99563229 |
| 61 | ST6GALNAC3 | -0.655236318 | 6.331865287 | -12.5266653 | 1.58E-27 | 4.06E-25 | 51.86367458 |
| 62 | MYH10 | 0.64580991 | 7.869406279 | 12.51020108 | 1.79E-27 | 4.51E-25 | 51.7441532 |
| 63 | SCUBE2 | 0.786764756 | 4.579511298 | 12.50330176 | 1.88E-27 | 4.64E-25 | 51.69407527 |
| 64 | SLC6A1 | 0.812965947 | 5.497776251 | 12.49773524 | 1.96E-27 | 4.78E-25 | 51.65367452 |
| 65 | BTN3A1 | 0.628864899 | 6.241678449 | 12.49506459 | 2.00E-27 | 4.81E-25 | 51.6342925 |
| 66 | CRYM | 0.961402813 | 7.363586063 | 12.49169655 | 2.05E-27 | 4.88E-25 | 51.60985016 |
| 67 | MMP16 | 0.762495355 | 4.327870992 | 12.46301474 | 2.53E-27 | 5.88E-25 | 51.40174511 |
| 68 | GPRASP1 | 0.625605194 | 5.761569276 | 12.40294644 | 3.93E-27 | 9.04E-25 | 50.9661649 |
| 69 | C16orf89 | 0.72329653 | 5.540191027 | 12.38390921 | 4.52E-27 | 1.01E-24 | 50.82819128 |
| 70 | CRISPLD1 | 0.812263626 | 3.973117258 | 12.36440711 | 5.21E-27 | 1.15E-24 | 50.68688559 |
| 71 | SEMA4B | -0.668637091 | 6.119723234 | -12.33107463 | 6.66E-27 | 1.45E-24 | 50.4454578 |
| 72 | DUSP13 | -0.680447498 | 5.537422096 | -12.29364452 | 8.76E-27 | 1.85E-24 | 50.17448571 |
| 73 | PHYHD1 | -0.559451161 | 7.273634339 | -12.18752263 | 1.91E-26 | 3.94E-24 | 49.40702521 |
| 74 | IFI44L | 1.085065007 | 6.949745048 | 12.16076036 | 2.32E-26 | 4.75E-24 | 49.21367595 |
| 75 | IL1RL1 | -1.701827581 | 6.187537068 | -12.13384263 | 2.83E-26 | 5.59E-24 | 49.01928379 |
| 76 | KCND3 | -0.533449029 | 6.96126565 | -12.13256598 | 2.85E-26 | 5.59E-24 | 49.01006616 |
| 77 | STK38L | 0.526391717 | 9.874688386 | 12.13178236 | 2.87E-26 | 5.59E-24 | 49.0044084 |
| 78 | STAT3 | -0.621370561 | 10.49582944 | -12.12609927 | 2.99E-26 | 5.77E-24 | 48.96337851 |
| 79 | ADAMTS9 | -1.184924985 | 8.34210753 | -12.11443689 | 3.26E-26 | 6.22E-24 | 48.87919156 |
| 80 | H2AFZ | -0.551693232 | 6.482950662 | -12.03970973 | 5.62E-26 | 1.05E-23 | 48.34013101 |
| 81 | AOX1 | -1.342440959 | 5.50382034 | -12.01397054 | 6.79E-26 | 1.26E-23 | 48.1546069 |
| 82 | DZIP3 | 0.57146204 | 6.715485684 | 12.01243276 | 6.86E-26 | 1.26E-23 | 48.14352534 |
| 83 | APBB3 | 0.528893079 | 7.015589503 | 11.98277409 | 8.52E-26 | 1.53E-23 | 47.92985369 |
| 84 | EXT1 | 0.540229045 | 7.423952998 | 11.97440385 | 9.06E-26 | 1.61E-23 | 47.86957072 |
| 85 | COL14A1 | 1.192838786 | 6.313870387 | 11.93005196 | 1.25E-25 | 2.13E-23 | 47.55028842 |
| 86 | FLJ34690 | 0.986066276 | 5.037803182 | 11.84224612 | 2.37E-25 | 3.79E-23 | 46.91891248 |
| 87 | LCN10 | -0.60293103 | 6.075857715 | -11.83522258 | 2.50E-25 | 3.95E-23 | 46.86845153 |
| 88 | USP31 | -0.5560173 | 8.937649108 | -11.82332449 | 2.72E-25 | 4.28E-23 | 46.78298372 |
| 89 | GLT8D2 | 0.800958528 | 7.24193074 | 11.8152527 | 2.89E-25 | 4.50E-23 | 46.72501202 |
| 90 | C1QTNF2 | 0.535286748 | 5.624908089 | 11.78223054 | 3.67E-25 | 5.59E-23 | 46.48793521 |
| 91 | SLC2A1 | -0.686992829 | 8.65810228 | -11.7763289 | 3.83E-25 | 5.80E-23 | 46.44558054 |
| 92 | LIPH | 0.70317956 | 3.88089959 | 11.74599808 | 4.78E-25 | 7.17E-23 | 46.22797704 |
| 93 | ZCCHC5 | 0.609669646 | 3.490225671 | 11.70364543 | 6.50E-25 | 9.55E-23 | 45.92433258 |
| 94 | EDNRB | -0.757973816 | 8.110537485 | -11.70028226 | 6.66E-25 | 9.71E-23 | 45.90023109 |
| 95 | ALOX5AP | -1.040013468 | 7.425588287 | -11.6853071 | 7.43E-25 | 1.07E-22 | 45.79293333 |
| 96 | ANKRD34C | 0.549036456 | 4.474089577 | 11.64645608 | 9.85E-25 | 1.39E-22 | 45.51470893 |
| 97 | FKBP5 | -1.110530455 | 9.356681272 | -11.64072288 | 1.03E-24 | 1.43E-22 | 45.47366955 |
| 98 | LTBP4 | 0.564397998 | 8.039828432 | 11.63448026 | 1.07E-24 | 1.48E-22 | 45.42898899 |
| 99 | CYP2J2 | 0.643408883 | 8.943288085 | 11.61481996 | 1.24E-24 | 1.68E-22 | 45.28830941 |
| 100 | PHLDA1 | 1.0414937 | 6.378260135 | 11.56747698 | 1.75E-24 | 2.33E-22 | 44.94977303 |
| 101 | ZNF844 | 0.56566626 | 5.81997527 | 11.53466389 | 2.21E-24 | 2.91E-22 | 44.71532667 |
| 102 | OGN | 1.23151926 | 7.395866153 | 11.52235746 | 2.42E-24 | 3.14E-22 | 44.62743926 |
| 103 | SIGLEC9 | -0.635506526 | 4.934750695 | -11.51215339 | 2.61E-24 | 3.36E-22 | 44.55458302 |
| 104 | SNAP47 | 0.669054841 | 6.306819946 | 11.50671747 | 2.71E-24 | 3.48E-22 | 44.51577735 |
| 105 | NT5E | 0.665486025 | 4.730566696 | 11.49294167 | 3.00E-24 | 3.79E-22 | 44.41745501 |
| 106 | CREB5 | 0.733142259 | 7.47788201 | 11.45375874 | 3.98E-24 | 4.97E-22 | 44.13794998 |
| 107 | SLC11A1 | -0.890346947 | 5.295488001 | -11.42287013 | 4.97E-24 | 6.07E-22 | 43.91777581 |
| 108 | SEZ6L | 0.526049766 | 4.577070955 | 11.40640672 | 5.60E-24 | 6.79E-22 | 43.80048464 |
| 109 | ANTXR1 | 0.566103165 | 8.206641439 | 11.34115897 | 8.96E-24 | 1.05E-21 | 43.3360531 |
| 110 | MAP3K6 | -0.529063316 | 7.646208622 | -11.32769057 | 9.88E-24 | 1.15E-21 | 43.24026937 |
| 111 | OSMR | -0.7314881 | 8.289974764 | -11.32576432 | 1.00E-23 | 1.16E-21 | 43.2265728 |
| 112 | RNASE2 | -1.144848436 | 4.455696556 | -11.31176079 | 1.11E-23 | 1.26E-21 | 43.12701854 |
| 113 | AMY2A | 0.555245113 | 7.227508211 | 11.30998409 | 1.12E-23 | 1.27E-21 | 43.11438983 |
| 114 | FGF14 | 0.753290645 | 4.926780878 | 11.2810459 | 1.38E-23 | 1.54E-21 | 42.9087704 |
| 115 | WNK3 | -0.73750227 | 3.863730494 | -11.24927911 | 1.74E-23 | 1.89E-21 | 42.68320982 |
| 116 | TCN2 | -0.592155714 | 6.637432588 | -11.24681557 | 1.77E-23 | 1.92E-21 | 42.66572434 |
| 117 | TRIL | 0.588252266 | 4.486580746 | 11.23929657 | 1.87E-23 | 2.00E-21 | 42.61236299 |
| 118 | TSPAN9 | 0.582831786 | 7.79741897 | 11.2020552 | 2.44E-23 | 2.57E-21 | 42.34820459 |
| 119 | CPE | 0.565268653 | 7.661976738 | 11.19687045 | 2.53E-23 | 2.66E-21 | 42.31144681 |
| 120 | FGF1 | 0.737074219 | 7.845238176 | 11.19029966 | 2.66E-23 | 2.77E-21 | 42.26486916 |
| 121 | IFIT3 | 0.529080611 | 5.675735921 | 11.15993252 | 3.30E-23 | 3.40E-21 | 42.0497037 |
| 122 | DDX60 | 0.525213148 | 5.564350839 | 11.14479578 | 3.68E-23 | 3.75E-21 | 41.94251156 |
| 123 | SLC36A4 | -0.658190811 | 6.015855166 | -11.1179842 | 4.46E-23 | 4.52E-21 | 41.7527399 |
| 124 | ARRDC3 | 0.674670752 | 8.412590386 | 11.07533037 | 6.06E-23 | 5.99E-21 | 41.45109495 |
| 125 | ANKMY2 | 0.527568292 | 7.314263533 | 11.04524971 | 7.52E-23 | 7.25E-21 | 41.23855935 |
| 126 | XAF1 | 0.541864224 | 7.789029711 | 11.01449101 | 9.37E-23 | 8.74E-21 | 41.02139997 |
| 127 | NAP1L3 | 1.020077832 | 5.438300879 | 11.00780051 | 9.83E-23 | 9.13E-21 | 40.97418688 |
| 128 | PCCB | 0.52251978 | 8.827839569 | 10.98327926 | 1.17E-22 | 1.06E-20 | 40.80121649 |
| 129 | OLFML1 | 0.831939216 | 5.639920077 | 10.95316186 | 1.45E-22 | 1.29E-20 | 40.58892168 |
| 130 | GPRASP2 | 0.509942203 | 5.728404596 | 10.92561874 | 1.77E-22 | 1.56E-20 | 40.3949192 |
| 131 | KLHL13 | 0.691630721 | 5.631673181 | 10.91653187 | 1.89E-22 | 1.65E-20 | 40.33094597 |
| 132 | NAMPT | -0.625277926 | 10.39601271 | -10.91369858 | 1.93E-22 | 1.68E-20 | 40.31100227 |
| 133 | KPNA2 | -0.557942984 | 8.139274224 | -10.91031975 | 1.97E-22 | 1.71E-20 | 40.28722035 |
| 134 | C15orf59 | -0.684768857 | 7.511633611 | -10.89982813 | 2.13E-22 | 1.83E-20 | 40.21338885 |
| 135 | MPP3 | -0.676853077 | 7.831146632 | -10.89908855 | 2.14E-22 | 1.83E-20 | 40.20818509 |
| 136 | RANBP17 | 0.529241299 | 5.299705151 | 10.897625 | 2.16E-22 | 1.85E-20 | 40.19788765 |
| 137 | CTSK | 0.744884873 | 6.738058364 | 10.88652515 | 2.34E-22 | 1.99E-20 | 40.11980304 |
| 138 | C1QTNF1 | -0.966164022 | 8.622602197 | -10.88432131 | 2.37E-22 | 2.01E-20 | 40.10430231 |
| 139 | NID1 | -0.564940729 | 8.996585551 | -10.88298691 | 2.40E-22 | 2.02E-20 | 40.09491732 |
| 140 | ALOX5 | -0.748892389 | 6.211744035 | -10.87235238 | 2.59E-22 | 2.16E-20 | 40.02013507 |
| 141 | MEOX2 | 0.533176362 | 7.144694435 | 10.84726862 | 3.09E-22 | 2.54E-20 | 39.84383075 |
| 142 | MT1X | -0.98686536 | 9.257404971 | -10.77648046 | 5.12E-22 | 4.03E-20 | 39.34694105 |
| 143 | FCER1G | -1.008973088 | 6.972735637 | -10.76752753 | 5.45E-22 | 4.28E-20 | 39.28416657 |
| 144 | LAMA4 | 0.529403622 | 8.262334882 | 10.76184207 | 5.68E-22 | 4.44E-20 | 39.24431054 |
| 145 | TEAD4 | -0.621990568 | 5.943346758 | -10.72126862 | 7.58E-22 | 5.81E-20 | 38.96006958 |
| 146 | DPT | 0.824713352 | 8.238037493 | 10.71198518 | 8.09E-22 | 6.16E-20 | 38.89507981 |
| 147 | SNCA | 0.743691983 | 7.135499047 | 10.6708588 | 1.08E-21 | 8.16E-20 | 38.60737868 |
| 148 | KIAA0040 | -0.577802427 | 6.958818301 | -10.66972159 | 1.09E-21 | 8.19E-20 | 38.59942822 |
| 149 | C1orf162 | -0.851902374 | 7.179020816 | -10.64700216 | 1.28E-21 | 9.52E-20 | 38.44064627 |
| 150 | OGDHL | 0.654535001 | 5.349474572 | 10.63679904 | 1.38E-21 | 1.02E-19 | 38.36937298 |
| 151 | SULF1 | 0.811773858 | 6.894260822 | 10.63490416 | 1.40E-21 | 1.03E-19 | 38.35613878 |
| 152 | RGS4 | 1.005310168 | 4.780726396 | 10.63332454 | 1.41E-21 | 1.04E-19 | 38.34510693 |
| 153 | MFAP4 | 0.864332503 | 7.835372612 | 10.63131358 | 1.43E-21 | 1.05E-19 | 38.33106351 |
| 154 | STEAP3 | -0.541775276 | 5.706379039 | -10.6014216 | 1.77E-21 | 1.29E-19 | 38.12241253 |
| 155 | ADORA3 | -0.755854261 | 5.040122597 | -10.5868184 | 1.96E-21 | 1.42E-19 | 38.02054739 |
| 156 | LRRC8A | -0.507172572 | 7.613515156 | -10.55736666 | 2.42E-21 | 1.71E-19 | 37.81524193 |
| 157 | MXRA5 | 1.232469458 | 7.188615616 | 10.54408062 | 2.66E-21 | 1.87E-19 | 37.72268608 |
| 158 | ZNF483 | 0.569341854 | 5.268425121 | 10.52697993 | 3.00E-21 | 2.10E-19 | 37.6036111 |
| 159 | S100A9 | -0.79211914 | 6.401498386 | -10.52694168 | 3.00E-21 | 2.10E-19 | 37.60334483 |
| 160 | BLM | -0.751623138 | 5.787375913 | -10.49823065 | 3.67E-21 | 2.54E-19 | 37.40356609 |
| 161 | LPCAT3 | -0.556023181 | 9.283610651 | -10.48243598 | 4.11E-21 | 2.82E-19 | 37.29373837 |
| 162 | FZD7 | 0.613248223 | 6.582500988 | 10.47248247 | 4.41E-21 | 3.01E-19 | 37.22455476 |
| 163 | C11orf52 | -0.574885394 | 5.153526755 | -10.42528049 | 6.14E-21 | 4.11E-19 | 36.89676389 |
| 164 | EPHX2 | 0.722428794 | 5.803158103 | 10.41895504 | 6.42E-21 | 4.28E-19 | 36.85287448 |
| 165 | CSRNP3 | 0.548846189 | 5.715598311 | 10.39071892 | 7.83E-21 | 5.17E-19 | 36.6570652 |
| 166 | EPHA3 | 0.546567829 | 6.390352636 | 10.38082504 | 8.40E-21 | 5.52E-19 | 36.5884961 |
| 167 | CYP4B1 | -1.411404869 | 7.181931874 | -10.37970736 | 8.46E-21 | 5.55E-19 | 36.58075146 |
| 168 | NT5DC2 | -0.5296346 | 8.455396747 | -10.35789136 | 9.87E-21 | 6.41E-19 | 36.42963969 |
| 169 | C20orf200 | 0.624132716 | 6.730808981 | 10.34283671 | 1.10E-20 | 7.10E-19 | 36.32542401 |
| 170 | ADAMTS15 | -0.658573061 | 6.143400946 | -10.32697343 | 1.23E-20 | 7.86E-19 | 36.21566628 |
| 171 | ECRP | -0.821239303 | 4.578915089 | -10.31354199 | 1.35E-20 | 8.55E-19 | 36.12277923 |
| 172 | C5orf23 | 1.048817504 | 7.591871606 | 10.31218287 | 1.36E-20 | 8.58E-19 | 36.11338235 |
| 173 | METTL7B | -1.107067292 | 7.498873793 | -10.2931433 | 1.55E-20 | 9.66E-19 | 35.98178824 |
| 174 | MID1IP1 | -0.619997993 | 6.897925337 | -10.28759509 | 1.62E-20 | 1.00E-18 | 35.94345692 |
| 175 | LAPTM5 | -0.865251599 | 8.104063979 | -10.2731507 | 1.79E-20 | 1.10E-18 | 35.84369713 |
| 176 | TMEM71 | 0.885807976 | 8.02693754 | 10.26445791 | 1.90E-20 | 1.16E-18 | 35.78368396 |
| 177 | ABCG2 | 0.683146635 | 4.923793579 | 10.25832007 | 1.98E-20 | 1.20E-18 | 35.74132016 |
| 178 | KAL1 | 0.676967684 | 6.072905738 | 10.24599556 | 2.16E-20 | 1.30E-18 | 35.65628211 |
| 179 | PHLDB2 | 0.536895654 | 8.718613952 | 10.23663553 | 2.31E-20 | 1.38E-18 | 35.59172245 |
| 180 | SAMD9L | 0.57651388 | 7.01313351 | 10.2137946 | 2.71E-20 | 1.58E-18 | 35.43426636 |
| 181 | PI16 | 1.058657489 | 6.786259032 | 10.20856984 | 2.81E-20 | 1.64E-18 | 35.39826625 |
| 182 | GNMT | -0.71880455 | 4.323091372 | -10.17207207 | 3.63E-20 | 2.08E-18 | 35.14696694 |
| 183 | NTM | 0.510357278 | 4.893262704 | 10.14959485 | 4.24E-20 | 2.40E-18 | 34.99236227 |
| 184 | CHAC2 | -0.57168249 | 4.731928394 | -10.13673011 | 4.64E-20 | 2.61E-18 | 34.90392969 |
| 185 | PLIN2 | -0.873724369 | 8.094893009 | -10.12143639 | 5.16E-20 | 2.88E-18 | 34.79885226 |
| 186 | ALPL | -0.539778432 | 6.53580279 | -10.1209092 | 5.18E-20 | 2.88E-18 | 34.7952312 |
| 187 | NPR3 | 0.956944269 | 7.727079481 | 10.09681969 | 6.13E-20 | 3.37E-18 | 34.62983979 |
| 188 | BEX1 | 0.917799327 | 5.195803079 | 10.09290023 | 6.30E-20 | 3.46E-18 | 34.60294335 |
| 189 | WASF1 | -0.510889305 | 4.074597707 | -10.06588873 | 7.60E-20 | 4.13E-18 | 34.4176856 |
| 190 | C13orf40 | 0.611311404 | 3.695753744 | 10.0541761 | 8.25E-20 | 4.47E-18 | 34.33741079 |
| 191 | C1orf105 | -0.964886245 | 4.970592654 | -10.04771595 | 8.62E-20 | 4.66E-18 | 34.29314942 |
| 192 | PLP2 | -0.606537524 | 9.181313716 | -10.02681734 | 9.97E-20 | 5.35E-18 | 34.15003495 |
| 193 | FMO4 | 0.681375875 | 6.812417153 | 10.00899651 | 1.13E-19 | 5.91E-18 | 34.02808349 |
| 194 | IL1R2 | -1.042864222 | 5.466763776 | -10.00040304 | 1.20E-19 | 6.25E-18 | 33.96930524 |
| 195 | MOXD1 | 0.819088024 | 5.921734489 | 9.986089719 | 1.32E-19 | 6.84E-18 | 33.87144526 |
| 196 | GNA14 | 0.598327967 | 4.761758225 | 9.706072562 | 9.14E-19 | 4.21E-17 | 31.96768677 |
| 197 | HERC6 | 0.615346598 | 6.730009942 | 9.694323804 | 9.91E-19 | 4.54E-17 | 31.88826936 |
| 198 | MGST1 | -1.201811287 | 7.38558196 | -9.685223611 | 1.05E-18 | 4.82E-17 | 31.82678145 |
| 199 | MAPK10 | 0.620829165 | 5.979537139 | 9.656423782 | 1.28E-18 | 5.83E-17 | 31.63233879 |
| 200 | FPR1 | -0.679347505 | 5.026367927 | -9.655347987 | 1.29E-18 | 5.86E-17 | 31.62508001 |
| 201 | LRRC17 | 0.788161413 | 4.846996873 | 9.647219093 | 1.37E-18 | 6.17E-17 | 31.5702418 |
| 202 | ANKRD2 | -1.01461189 | 9.444927087 | -9.64247734 | 1.41E-18 | 6.33E-17 | 31.53826206 |
| 203 | C20orf26 | 0.688744269 | 7.849965022 | 9.64154886 | 1.42E-18 | 6.36E-17 | 31.53200086 |
| 204 | MTHFD2 | -0.626002804 | 7.716425393 | -9.631168752 | 1.53E-18 | 6.77E-17 | 31.4620191 |
| 205 | ENAM | 0.816951019 | 6.321749882 | 9.630031019 | 1.54E-18 | 6.81E-17 | 31.45435044 |
| 206 | JAK2 | 0.632206275 | 8.054767975 | 9.628875843 | 1.55E-18 | 6.85E-17 | 31.44656459 |
| 207 | CHDH | -0.708669553 | 5.135316985 | -9.606493419 | 1.81E-18 | 7.91E-17 | 31.29578178 |
| 208 | SVEP1 | 0.76518994 | 6.626307414 | 9.578961045 | 2.18E-18 | 9.53E-17 | 31.11049981 |
| 209 | ANKRD29 | 0.554171697 | 5.784709699 | 9.574818813 | 2.25E-18 | 9.76E-17 | 31.08264287 |
| 210 | IFITM2 | -0.504251933 | 8.430308126 | -9.55808965 | 2.52E-18 | 1.09E-16 | 30.97018738 |
| 211 | IL18R1 | -0.618050895 | 4.575278405 | -9.52978565 | 3.05E-18 | 1.29E-16 | 30.78010717 |
| 212 | CEBPB | -0.653433349 | 8.176426893 | -9.447462882 | 5.35E-18 | 2.16E-16 | 30.22857634 |
| 213 | THBS4 | 0.962307356 | 7.987550331 | 9.420270612 | 6.43E-18 | 2.55E-16 | 30.04683616 |
| 214 | ITGBL1 | 0.684724757 | 5.568427052 | 9.409998274 | 6.89E-18 | 2.71E-16 | 29.97823792 |
| 215 | C13orf33 | -0.949332467 | 6.975620286 | -9.406333679 | 7.07E-18 | 2.76E-16 | 29.95377352 |
| 216 | SH3PXD2B | -0.629221032 | 6.745407928 | -9.394359882 | 7.66E-18 | 2.99E-16 | 29.87386587 |
| 217 | ATP1B3 | -0.759941619 | 6.949033482 | -9.392484639 | 7.76E-18 | 3.02E-16 | 29.86135524 |
| 218 | KBTBD10 | -0.628371845 | 9.001042676 | -9.392352539 | 7.77E-18 | 3.02E-16 | 29.86047398 |
| 219 | BBS12 | -0.576707134 | 5.067120224 | -9.369908871 | 9.05E-18 | 3.48E-16 | 29.71082457 |
| 220 | AGPAT9 | 0.576608016 | 7.290046382 | 9.368379873 | 9.14E-18 | 3.51E-16 | 29.70063507 |
| 221 | TIMP4 | -0.712395474 | 8.607848461 | -9.336117986 | 1.14E-17 | 4.31E-16 | 29.48580129 |
| 222 | SLC40A1 | 0.5676649 | 7.986888651 | 9.32445668 | 1.23E-17 | 4.66E-16 | 29.40822579 |
| 223 | FMOD | 0.989903077 | 6.737600775 | 9.321726698 | 1.25E-17 | 4.73E-16 | 29.39007088 |
| 224 | SESN3 | 0.575286748 | 5.790099434 | 9.301277629 | 1.44E-17 | 5.38E-16 | 29.2541532 |
| 225 | PLXDC1 | 0.568088099 | 7.83187578 | 9.214343175 | 2.58E-17 | 9.20E-16 | 28.67777332 |
| 226 | TTLL7 | 0.554142756 | 6.280758757 | 9.214058174 | 2.59E-17 | 9.20E-16 | 28.67588763 |
| 227 | CGNL1 | 0.677758101 | 6.277301058 | 9.212567319 | 2.62E-17 | 9.28E-16 | 28.66602388 |
| 228 | MIR99A | 0.652935819 | 5.380536746 | 9.208904898 | 2.68E-17 | 9.47E-16 | 28.64179566 |
| 229 | GNB3 | 0.542227339 | 6.115598298 | 9.206217612 | 2.73E-17 | 9.61E-16 | 28.624021 |
| 230 | CRISPLD2 | -0.605989419 | 7.458606948 | -9.186022331 | 3.13E-17 | 1.09E-15 | 28.49051512 |
| 231 | SFRP1 | 1.015450702 | 6.789049275 | 9.177238739 | 3.32E-17 | 1.15E-15 | 28.43248928 |
| 232 | CCL5 | 0.690551606 | 5.402036514 | 9.154366055 | 3.87E-17 | 1.32E-15 | 28.28150368 |
| 233 | CYP4Z1 | -0.669362437 | 3.881895288 | -9.153577949 | 3.89E-17 | 1.33E-15 | 28.27630427 |
| 234 | BCL3 | -0.602960242 | 6.829244904 | -9.13621866 | 4.37E-17 | 1.48E-15 | 28.16182915 |
| 235 | GRB14 | -0.530503436 | 5.946757322 | -9.129316321 | 4.57E-17 | 1.55E-15 | 28.1163388 |
| 236 | TNNT1 | 0.705156138 | 7.20927187 | 9.12759978 | 4.62E-17 | 1.56E-15 | 28.1050282 |
| 237 | LTBP2 | 0.999790886 | 8.01841703 | 9.120001692 | 4.87E-17 | 1.64E-15 | 28.05497437 |
| 238 | HAPLN1 | 1.133912211 | 4.078172752 | 9.105342089 | 5.37E-17 | 1.80E-15 | 27.95845409 |
| 239 | JPH1 | -0.548940303 | 7.641235119 | -9.105335975 | 5.37E-17 | 1.80E-15 | 27.95841385 |
| 240 | HTR4 | 0.65377228 | 4.634991833 | 9.101016865 | 5.53E-17 | 1.85E-15 | 27.92998965 |
| 241 | RASL11B | 0.836189906 | 5.854483816 | 9.099900313 | 5.57E-17 | 1.86E-15 | 27.92264258 |
| 242 | ITGA5 | -0.803361328 | 8.507793554 | -9.080729777 | 6.33E-17 | 2.09E-15 | 27.79656063 |
| 243 | MT1A | -0.98908453 | 7.970784977 | -9.059439739 | 7.29E-17 | 2.38E-15 | 27.65667923 |
| 244 | PPP1R3C | 0.612700989 | 9.540730527 | 9.059318243 | 7.30E-17 | 2.38E-15 | 27.65588139 |
| 245 | PAPSS2 | -0.517689338 | 7.586863382 | -9.046584474 | 7.95E-17 | 2.57E-15 | 27.57228838 |
| 246 | MYOT | -0.963056796 | 7.398739454 | -9.041281796 | 8.23E-17 | 2.65E-15 | 27.53749373 |
| 247 | SGPP2 | -1.146915835 | 6.40735105 | -9.038902026 | 8.37E-17 | 2.69E-15 | 27.52188137 |
| 248 | FLJ30064 | -0.567336119 | 4.772672677 | -9.009652235 | 1.02E-16 | 3.23E-15 | 27.33014212 |
| 249 | CNN1 | -0.895589948 | 9.244087141 | -9.00044796 | 1.08E-16 | 3.43E-15 | 27.26986449 |
| 250 | PIK3IP1 | 0.606945854 | 5.908612689 | 8.996474437 | 1.11E-16 | 3.51E-15 | 27.24385109 |
| 251 | FCGBP | -0.60729094 | 4.464130747 | -8.996139951 | 1.11E-16 | 3.51E-15 | 27.24166156 |
| 252 | CX3CR1 | 0.562512643 | 4.237481068 | 8.984676353 | 1.20E-16 | 3.77E-15 | 27.16664371 |
| 253 | SLCO2A1 | -0.798060512 | 7.771538835 | -8.972968115 | 1.30E-16 | 4.06E-15 | 27.0900702 |
| 254 | RSAD2 | 0.600129716 | 5.037544114 | 8.966004207 | 1.36E-16 | 4.24E-15 | 27.04454701 |
| 255 | MAP2K1 | -0.507281033 | 7.837628095 | -8.952362016 | 1.49E-16 | 4.62E-15 | 26.9554149 |
| 256 | ZDHHC9 | -0.60640523 | 8.093323956 | -8.949436413 | 1.52E-16 | 4.71E-15 | 26.93630843 |
| 257 | ZNF536 | 0.5716992 | 5.025933018 | 8.935189283 | 1.67E-16 | 5.12E-15 | 26.84330478 |
| 258 | PVR | -0.589787935 | 8.108833036 | -8.929886567 | 1.73E-16 | 5.27E-15 | 26.80870674 |
| 259 | PLTP | -0.773534197 | 8.803749676 | -8.916641675 | 1.89E-16 | 5.72E-15 | 26.72233086 |
| 260 | DIO2 | 0.663131066 | 4.354058057 | 8.909514393 | 1.98E-16 | 5.96E-15 | 26.67587527 |
| 261 | RPGR | -0.527496472 | 6.041999448 | -8.889399109 | 2.26E-16 | 6.72E-15 | 26.54485721 |
| 262 | TLR2 | -0.722032593 | 5.265285056 | -8.846384472 | 3.00E-16 | 8.77E-15 | 26.26515223 |
| 263 | ZMYND17 | 0.746052018 | 6.04785621 | 8.799015877 | 4.10E-16 | 1.17E-14 | 25.95787494 |
| 264 | GATSL1 | -0.572767549 | 7.916330787 | -8.789921532 | 4.36E-16 | 1.23E-14 | 25.89896989 |
| 265 | NQO1 | -0.632869362 | 6.281798104 | -8.786249639 | 4.46E-16 | 1.26E-14 | 25.87519485 |
| 266 | SULT1B1 | -0.622852497 | 3.90264411 | -8.781033278 | 4.62E-16 | 1.30E-14 | 25.84142769 |
| 267 | SHISA3 | -0.933455385 | 6.545032273 | -8.764787667 | 5.14E-16 | 1.44E-14 | 25.73632597 |
| 268 | ETNK2 | -0.723426187 | 5.20648143 | -8.756151844 | 5.44E-16 | 1.51E-14 | 25.68049394 |
| 269 | SERPINB8 | -0.751280269 | 5.988436305 | -8.707792104 | 7.47E-16 | 2.04E-14 | 25.36832847 |
| 270 | CXCL12 | 0.506157552 | 8.778625628 | 8.688894593 | 8.46E-16 | 2.28E-14 | 25.24657025 |
| 271 | TUBA1C | -0.755241181 | 7.112588312 | -8.679674876 | 8.99E-16 | 2.42E-14 | 25.18721334 |
| 272 | NUDT4 | -0.506630095 | 7.232806114 | -8.65533019 | 1.05E-15 | 2.82E-14 | 25.03062843 |
| 273 | SNORD115-20 | 0.512991531 | 2.509771322 | 8.64845422 | 1.10E-15 | 2.92E-14 | 24.98644101 |
| 274 | GPSM2 | -0.701500136 | 4.843881992 | -8.63795118 | 1.18E-15 | 3.10E-14 | 24.91897784 |
| 275 | CPAMD8 | -0.515371492 | 5.988375019 | -8.616299123 | 1.36E-15 | 3.55E-14 | 24.78002887 |
| 276 | SAT1 | -0.553148877 | 9.207154908 | -8.597155738 | 1.54E-15 | 3.97E-14 | 24.65732156 |
| 277 | OMD | 0.90368761 | 6.517731734 | 8.565720142 | 1.89E-15 | 4.78E-14 | 24.45611433 |
| 278 | PROM1 | 0.827204083 | 6.053464609 | 8.5646659 | 1.90E-15 | 4.81E-14 | 24.44937285 |
| 279 | HMCN1 | 0.569285771 | 5.983852376 | 8.547434109 | 2.13E-15 | 5.34E-14 | 24.33924032 |
| 280 | DPYSL3 | 0.511374683 | 6.804030498 | 8.517135133 | 2.59E-15 | 6.45E-14 | 24.14585993 |
| 281 | SLC7A8 | -0.620620873 | 7.745935533 | -8.500515959 | 2.89E-15 | 7.13E-14 | 24.03993527 |
| 282 | CPM | -0.831239566 | 7.467225534 | -8.479864717 | 3.30E-15 | 8.06E-14 | 23.90845618 |
| 283 | C6orf186 | 0.552542777 | 5.191768206 | 8.437464631 | 4.34E-15 | 1.04E-13 | 23.63901515 |
| 284 | CCR1 | -0.695623498 | 4.976360794 | -8.436128437 | 4.38E-15 | 1.05E-13 | 23.6305351 |
| 285 | CD38 | -0.585864268 | 6.347311533 | -8.426990769 | 4.65E-15 | 1.11E-13 | 23.57256185 |
| 286 | GFPT2 | -0.853321639 | 6.66016146 | -8.422225764 | 4.79E-15 | 1.14E-13 | 23.54234329 |
| 287 | HOPX | -0.62916414 | 5.381775382 | -8.404822034 | 5.36E-15 | 1.26E-13 | 23.43204669 |
| 288 | F3 | 0.535025194 | 6.85780821 | 8.400620142 | 5.51E-15 | 1.29E-13 | 23.40543449 |
| 289 | MIRLET7A2 | 0.521503629 | 3.949741194 | 8.399530105 | 5.55E-15 | 1.30E-13 | 23.39853198 |
| 290 | S100A8 | -1.12956378 | 7.119050636 | -8.2913151 | 1.11E-14 | 2.47E-13 | 22.71556747 |
| 291 | F13A1 | -0.816026228 | 9.227929299 | -8.272055803 | 1.26E-14 | 2.76E-13 | 22.59449911 |
| 292 | RARRES1 | -0.961096795 | 5.651066392 | -8.270493652 | 1.27E-14 | 2.78E-13 | 22.5846855 |
| 293 | TXNRD1 | -0.568408723 | 7.871593892 | -8.269647514 | 1.28E-14 | 2.79E-13 | 22.57937037 |
| 294 | SERPINE1 | -1.635061974 | 7.200520171 | -8.26444455 | 1.32E-14 | 2.89E-13 | 22.54669347 |
| 295 | SCN7A | 0.624763975 | 8.466179748 | 8.242478453 | 1.52E-14 | 3.30E-13 | 22.40885523 |
| 296 | CEBPD | -0.674655355 | 8.477508207 | -8.239100647 | 1.55E-14 | 3.37E-13 | 22.38767638 |
| 297 | NT5DC3 | -0.568906936 | 5.529002676 | -8.227753448 | 1.67E-14 | 3.61E-13 | 22.31656277 |
| 298 | GSTM5 | 0.665023449 | 5.608221364 | 8.200551582 | 1.99E-14 | 4.23E-13 | 22.14629686 |
| 299 | KCNMB2 | 0.529776969 | 4.813555022 | 8.19954651 | 2.00E-14 | 4.26E-13 | 22.14001146 |
| 300 | TPCN1 | 0.539550636 | 9.146487153 | 8.186434448 | 2.17E-14 | 4.59E-13 | 22.05805006 |
| 301 | MYBL1 | 0.509688973 | 6.233141536 | 8.089703188 | 4.01E-14 | 8.19E-13 | 21.45555614 |
| 302 | HLA-DPB1 | 0.536125411 | 9.291296554 | 8.081843368 | 4.22E-14 | 8.55E-13 | 21.40676939 |
| 303 | C10orf71 | 0.787107747 | 8.886868155 | 8.050643644 | 5.14E-14 | 1.03E-12 | 21.21336154 |
| 304 | GATM | 0.706146645 | 7.849538235 | 8.045511514 | 5.31E-14 | 1.05E-12 | 21.18158605 |
| 305 | AREG | -1.158627088 | 6.287471054 | -8.039353166 | 5.52E-14 | 1.09E-12 | 21.14347123 |
| 306 | SNORD115-11 | 0.778766463 | 4.389425178 | 8.01291278 | 6.52E-14 | 1.28E-12 | 20.98000822 |
| 307 | SRGN | -0.504866709 | 9.208981253 | -8.01122486 | 6.59E-14 | 1.29E-12 | 20.96958289 |
| 308 | COL15A1 | 0.506114153 | 7.546411648 | 8.010359451 | 6.62E-14 | 1.30E-12 | 20.96423821 |
| 309 | SNORD115-12 | 0.755575529 | 4.399220828 | 8.004850609 | 6.86E-14 | 1.34E-12 | 20.93022354 |
| 310 | INMT | 0.522546353 | 7.871558522 | 7.98670073 | 7.68E-14 | 1.49E-12 | 20.81824631 |
| 311 | SNORD115-26 | 0.774381787 | 4.392317536 | 7.975927907 | 8.22E-14 | 1.59E-12 | 20.75184816 |
| 312 | IGSF10 | 0.739375668 | 5.012306269 | 7.969498687 | 8.56E-14 | 1.64E-12 | 20.71224512 |
| 313 | C1QB | -0.694938795 | 8.063093732 | -7.968157944 | 8.63E-14 | 1.66E-12 | 20.70398854 |
| 314 | ARRDC4 | -0.647321608 | 7.411333607 | -7.94214834 | 1.02E-13 | 1.91E-12 | 20.54396695 |
| 315 | STEAP4 | -0.588799811 | 8.460600241 | -7.915484932 | 1.20E-13 | 2.22E-12 | 20.38022204 |
| 316 | DSC1 | 0.969451732 | 6.400761034 | 7.900952382 | 1.32E-13 | 2.42E-12 | 20.29110311 |
| 317 | C5AR1 | -0.545410193 | 5.69969797 | -7.893628174 | 1.38E-13 | 2.52E-12 | 20.24622277 |
| 318 | CRHBP | 0.530723985 | 4.902336359 | 7.888761324 | 1.42E-13 | 2.59E-12 | 20.21641307 |
| 319 | PODN | 0.634090854 | 7.912801801 | 7.872280788 | 1.57E-13 | 2.85E-12 | 20.11554485 |
| 320 | GPR133 | -0.536655834 | 8.039692248 | -7.845454562 | 1.86E-13 | 3.34E-12 | 19.95160779 |
| 321 | NAP1L2 | 0.519631736 | 4.927540177 | 7.843254612 | 1.88E-13 | 3.38E-12 | 19.93817759 |
| 322 | MIRLET7C | 0.561422016 | 3.563389262 | 7.829691122 | 2.05E-13 | 3.66E-12 | 19.85542212 |
| 323 | CCDC3 | 0.627442705 | 6.407124553 | 7.814971294 | 2.25E-13 | 3.98E-12 | 19.76570229 |
| 324 | MIR208A | -1.175411825 | 5.052548072 | -7.810550087 | 2.31E-13 | 4.08E-12 | 19.7387728 |
| 325 | STAT4 | 0.893573093 | 5.559369273 | 7.794386233 | 2.55E-13 | 4.47E-12 | 19.64039203 |
| 326 | CTSC | -0.778244913 | 7.492557913 | -7.782545626 | 2.75E-13 | 4.78E-12 | 19.56839743 |
| 327 | CD53 | -0.684021815 | 7.508974684 | -7.78222374 | 2.75E-13 | 4.79E-12 | 19.56644113 |
| 328 | MRC1 | -0.70168743 | 7.713456419 | -7.77433521 | 2.89E-13 | 5.01E-12 | 19.51851181 |
| 329 | CHL1 | -0.73233121 | 5.647678261 | -7.74105511 | 3.55E-13 | 6.05E-12 | 19.31661084 |
| 330 | CDKN1A | -0.638602813 | 6.439103534 | -7.725469796 | 3.91E-13 | 6.61E-12 | 19.22222826 |
| 331 | FGF7 | -0.518247336 | 5.615993978 | -7.722012145 | 4.00E-13 | 6.74E-12 | 19.20130387 |
| 332 | AGXT2L1 | -0.845531029 | 5.277044706 | -7.701269389 | 4.54E-13 | 7.62E-12 | 19.07588875 |
| 333 | PNP | -0.725073579 | 6.103588778 | -7.668487851 | 5.56E-13 | 9.18E-12 | 18.87807832 |
| 334 | SNORD115-32 | 0.58873285 | 3.079104925 | 7.657568519 | 5.94E-13 | 9.75E-12 | 18.81229642 |
| 335 | CD14 | -0.759384952 | 8.23409335 | -7.646528346 | 6.36E-13 | 1.03E-11 | 18.74584139 |
| 336 | GBP4 | 0.528187366 | 6.033620641 | 7.644407383 | 6.44E-13 | 1.05E-11 | 18.73308082 |
| 337 | F2R | 0.51555053 | 7.049200073 | 7.632091704 | 6.95E-13 | 1.12E-11 | 18.65902514 |
| 338 | SLA | -0.542384586 | 5.355573795 | -7.623902477 | 7.31E-13 | 1.18E-11 | 18.6098205 |
| 339 | WNT9A | 0.673427956 | 5.761186138 | 7.572201445 | 1.00E-12 | 1.58E-11 | 18.29988454 |
| 340 | ATRNL1 | 0.992293397 | 5.06990658 | 7.566838707 | 1.04E-12 | 1.62E-11 | 18.26780639 |
| 341 | SNORD115-1 | 0.670535147 | 4.587714665 | 7.563890315 | 1.05E-12 | 1.64E-11 | 18.25017572 |
| 342 | RXRG | 0.554712384 | 5.054368424 | 7.524233597 | 1.34E-12 | 2.07E-11 | 18.01342888 |
| 343 | CADPS | 0.557663659 | 6.789150507 | 7.518619872 | 1.39E-12 | 2.13E-11 | 17.97997444 |
| 344 | PDE1A | 0.747331735 | 7.338308558 | 7.515200811 | 1.42E-12 | 2.17E-11 | 17.95960607 |
| 345 | C1QC | -0.606226501 | 7.782641897 | -7.505871834 | 1.50E-12 | 2.29E-11 | 17.90405829 |
| 346 | NPPA | 1.980988928 | 7.258830726 | 7.490435852 | 1.65E-12 | 2.49E-11 | 17.81223667 |
| 347 | ZNF676 | 0.622046569 | 6.118696722 | 7.487209502 | 1.68E-12 | 2.54E-11 | 17.79305867 |
| 348 | IL1R1 | -0.571862385 | 7.63579666 | -7.46926011 | 1.88E-12 | 2.80E-11 | 17.68645345 |
| 349 | NNMT | -0.631748144 | 7.497591455 | -7.448632401 | 2.13E-12 | 3.15E-11 | 17.56412831 |
| 350 | ELL2 | -0.571779672 | 7.722970567 | -7.430927194 | 2.37E-12 | 3.46E-11 | 17.45929424 |
| 351 | SLC7A1 | -0.651467238 | 7.124861302 | -7.420057667 | 2.53E-12 | 3.69E-11 | 17.3950084 |
| 352 | ARG2 | -0.806086361 | 5.184872076 | -7.409318242 | 2.69E-12 | 3.91E-11 | 17.33154715 |
| 353 | HAS2 | -0.893112417 | 5.571235362 | -7.392968843 | 2.97E-12 | 4.29E-11 | 17.23504098 |
| 354 | CCDC141 | 0.627528662 | 8.317968844 | 7.390005741 | 3.03E-12 | 4.36E-11 | 17.21756422 |
| 355 | FAM83B | -0.73625627 | 4.261356896 | -7.389641508 | 3.03E-12 | 4.36E-11 | 17.21541622 |
| 356 | TIMP1 | -0.741520945 | 10.12543445 | -7.373712273 | 3.34E-12 | 4.77E-11 | 17.12153831 |
| 357 | IFI30 | -0.699176788 | 7.634930456 | -7.372535329 | 3.36E-12 | 4.80E-11 | 17.11460688 |
| 358 | KLF15 | -0.509551453 | 7.547666789 | -7.360031875 | 3.63E-12 | 5.16E-11 | 17.04101086 |
| 359 | CCDC80 | 0.702217425 | 8.809116903 | 7.333641415 | 4.25E-12 | 5.98E-11 | 16.88592211 |
| 360 | MT1M | -0.952373271 | 7.456510758 | -7.314463101 | 4.77E-12 | 6.64E-11 | 16.77342803 |
| 361 | SOCS3 | -0.677420054 | 5.384676306 | -7.301950127 | 5.14E-12 | 7.10E-11 | 16.70012692 |
| 362 | C4orf29 | 0.581060746 | 7.957999153 | 7.26835595 | 6.28E-12 | 8.58E-11 | 16.50370912 |
| 363 | GALNTL2 | -0.860328953 | 6.322120522 | -7.259475679 | 6.62E-12 | 9.00E-11 | 16.45188029 |
| 364 | APLNR | 0.619123721 | 5.899523971 | 7.245210153 | 7.21E-12 | 9.74E-11 | 16.36870195 |
| 365 | SAMD12 | 0.566775276 | 6.177952265 | 7.243892542 | 7.26E-12 | 9.81E-11 | 16.36102436 |
| 366 | PI15 | -1.002185889 | 3.926182114 | -7.189952517 | 1.00E-11 | 1.32E-10 | 16.04745703 |
| 367 | CD68 | -0.562892513 | 8.620354185 | -7.181726939 | 1.05E-11 | 1.38E-10 | 15.99976634 |
| 368 | UCHL1 | 0.724432902 | 5.93190651 | 7.169484988 | 1.13E-11 | 1.48E-10 | 15.92885168 |
| 369 | SNORD115-42 | 0.623734901 | 4.43204733 | 7.164117873 | 1.17E-11 | 1.52E-10 | 15.89778486 |
| 370 | CA14 | -0.597532278 | 5.366272696 | -7.153191666 | 1.24E-11 | 1.62E-10 | 15.83458447 |
| 371 | GADD45B | -0.541659004 | 6.861223642 | -7.152569611 | 1.25E-11 | 1.62E-10 | 15.83098811 |
| 372 | FAM134B | 0.523843229 | 9.025053047 | 7.138684048 | 1.36E-11 | 1.75E-10 | 15.75076052 |
| 373 | FLJ34503 | 0.887275369 | 5.575315605 | 7.136513441 | 1.37E-11 | 1.77E-10 | 15.738228 |
| 374 | SNORD115-6 | 0.598981177 | 4.414780972 | 7.104914918 | 1.65E-11 | 2.10E-10 | 15.55605472 |
| 375 | CPXM2 | 0.514038775 | 5.159382259 | 7.098385904 | 1.72E-11 | 2.17E-10 | 15.51847613 |
| 376 | MSR1 | -0.555915604 | 5.166513526 | -7.092899607 | 1.78E-11 | 2.24E-10 | 15.48691572 |
| 377 | CES1 | 0.551137892 | 7.093688298 | 7.070652844 | 2.02E-11 | 2.53E-10 | 15.35909561 |
| 378 | COL12A1 | 0.516720371 | 7.311799739 | 7.064712453 | 2.10E-11 | 2.61E-10 | 15.32500728 |
| 379 | LPHN3 | 0.644734497 | 6.838717765 | 7.05919678 | 2.16E-11 | 2.69E-10 | 15.29337223 |
| 380 | SLC27A6 | 0.73922947 | 8.083207594 | 7.047024046 | 2.32E-11 | 2.87E-10 | 15.22361064 |
| 381 | PCDHB5 | 0.520924937 | 6.687335082 | 7.041248081 | 2.40E-11 | 2.96E-10 | 15.19053521 |
| 382 | P2RX5 | -0.643482131 | 6.610943578 | -6.996828065 | 3.12E-11 | 3.74E-10 | 14.93674051 |
| 383 | PIM1 | -0.546765996 | 7.294072899 | -6.976868633 | 3.50E-11 | 4.18E-10 | 14.82303275 |
| 384 | SLC25A18 | -0.589763113 | 5.495053597 | -6.971169047 | 3.62E-11 | 4.31E-10 | 14.79060034 |
| 385 | ATP1B4 | 0.574798486 | 3.655828653 | 6.883950901 | 6.00E-11 | 6.91E-10 | 14.296413 |
| 386 | CHRDL2 | -0.822166234 | 5.126845928 | -6.879692633 | 6.15E-11 | 7.06E-10 | 14.27238727 |
| 387 | LOC121952 | 0.766682447 | 6.969483902 | 6.865758426 | 6.67E-11 | 7.61E-10 | 14.19383542 |
| 388 | C6 | 0.988130916 | 8.315089928 | 6.820574379 | 8.65E-11 | 9.62E-10 | 13.9398249 |
| 389 | XRCC4 | -0.637485329 | 6.56167441 | -6.782492165 | 1.08E-10 | 1.18E-09 | 13.72658327 |
| 390 | FAM155B | -0.735203747 | 6.966003191 | -6.764213469 | 1.19E-10 | 1.30E-09 | 13.62450768 |
| 391 | PPM1K | 0.579806542 | 9.096345791 | 6.755309391 | 1.26E-10 | 1.36E-09 | 13.57484885 |
| 392 | MYC | -0.653953544 | 6.713110418 | -6.750671309 | 1.29E-10 | 1.39E-09 | 13.54899879 |
| 393 | POGLUT1 | -0.526602736 | 5.448167344 | -6.737318542 | 1.39E-10 | 1.49E-09 | 13.47464286 |
| 394 | TNNI3K | 0.560196608 | 7.52604894 | 6.709710581 | 1.63E-10 | 1.72E-09 | 13.32121196 |
| 395 | ADAMTS1 | -0.522103829 | 7.525522792 | -6.679818594 | 1.93E-10 | 2.02E-09 | 13.15555502 |
| 396 | MIR23B | 0.505865018 | 7.359104001 | 6.647872072 | 2.31E-10 | 2.38E-09 | 12.97905224 |
| 397 | AEBP1 | 0.536270236 | 7.005651786 | 6.634480245 | 2.50E-10 | 2.54E-09 | 12.90522987 |
| 398 | DHCR24 | -0.553426249 | 7.756937431 | -6.63235768 | 2.53E-10 | 2.57E-09 | 12.89353831 |
| 399 | C5orf13 | 0.572982805 | 8.050689436 | 6.621037332 | 2.69E-10 | 2.73E-09 | 12.83122531 |
| 400 | TNNI1 | 0.625538986 | 5.883060413 | 6.619972124 | 2.71E-10 | 2.74E-09 | 12.8253655 |
| 401 | MYOC | 0.628579518 | 4.836502962 | 6.614161977 | 2.80E-10 | 2.82E-09 | 12.79341438 |
| 402 | SCGN | -0.601768243 | 3.724368575 | -6.610116159 | 2.86E-10 | 2.88E-09 | 12.77117667 |
| 403 | LRRC10 | 0.77000728 | 6.914964944 | 6.608900431 | 2.88E-10 | 2.90E-09 | 12.76449623 |
| 404 | EDA2R | 0.563038635 | 5.393057545 | 6.584214714 | 3.31E-10 | 3.31E-09 | 12.62902518 |
| 405 | C3 | -0.663568312 | 9.77486209 | -6.562316104 | 3.75E-10 | 3.72E-09 | 12.50913291 |
| 406 | C3AR1 | -0.589379992 | 6.726272171 | -6.549909511 | 4.02E-10 | 3.97E-09 | 12.44132703 |
| 407 | GABRB1 | 0.563658457 | 5.869717355 | 6.541189882 | 4.22E-10 | 4.15E-09 | 12.39372307 |
| 408 | TNNT3 | 0.526468377 | 7.459022386 | 6.536582811 | 4.33E-10 | 4.25E-09 | 12.36858842 |
| 409 | ADAMTS5 | -0.536408826 | 6.549905575 | -6.516545779 | 4.84E-10 | 4.71E-09 | 12.2594116 |
| 410 | RNF157 | -0.528704742 | 6.310945439 | -6.47385843 | 6.14E-10 | 5.87E-09 | 12.0275732 |
| 411 | KLRK1 | 0.555123274 | 3.890086245 | 6.460019289 | 6.63E-10 | 6.31E-09 | 11.95263311 |
| 412 | C10orf110 | 0.515478949 | 5.413872359 | 6.449604812 | 7.03E-10 | 6.65E-09 | 11.89630962 |
| 413 | MIR30E | 0.558080272 | 5.812649301 | 6.445714125 | 7.18E-10 | 6.79E-09 | 11.87528388 |
| 414 | ZFP36 | -0.570027997 | 7.292962306 | -6.429802634 | 7.84E-10 | 7.36E-09 | 11.78938608 |
| 415 | F2RL2 | 0.594991185 | 4.903746124 | 6.423699821 | 8.11E-10 | 7.60E-09 | 11.75647855 |
| 416 | NEB | 1.104453651 | 5.878308541 | 6.401715424 | 9.16E-10 | 8.49E-09 | 11.63811121 |
| 417 | SPHKAP | 0.520607521 | 9.513217135 | 6.395515104 | 9.48E-10 | 8.77E-09 | 11.60477786 |
| 418 | CCL2 | -0.96602324 | 8.512025676 | -6.315899766 | 1.47E-09 | 1.31E-08 | 11.17873168 |
| 419 | HSPA2 | 0.633978822 | 7.296326316 | 6.303334864 | 1.57E-09 | 1.40E-08 | 11.11182911 |
| 420 | SPP1 | -0.966479924 | 6.788798497 | -6.290845257 | 1.68E-09 | 1.49E-08 | 11.04541877 |
| 421 | PAR5 | 0.525630858 | 8.405066914 | 6.211085527 | 2.60E-09 | 2.22E-08 | 10.62347684 |
| 422 | HBB | 1.386507504 | 7.320363669 | 6.197658153 | 2.79E-09 | 2.38E-08 | 10.5528134 |
| 423 | COLQ | 0.670460364 | 6.255144174 | 6.175265051 | 3.15E-09 | 2.66E-08 | 10.43520499 |
| 424 | HFE2 | 0.563828415 | 6.928902224 | 6.152027057 | 3.57E-09 | 2.99E-08 | 10.31347544 |
| 425 | ADH1A | -0.541675118 | 6.675536088 | -6.124803053 | 4.14E-09 | 3.42E-08 | 10.17127696 |
| 426 | PTX3 | -1.008650017 | 5.018115451 | -6.093741887 | 4.88E-09 | 4.00E-08 | 10.00958041 |
| 427 | LMCD1 | -0.733123238 | 7.285463787 | -6.001154914 | 7.99E-09 | 6.31E-08 | 9.531067656 |
| 428 | THBS1 | -0.741520495 | 9.553286269 | -5.990510953 | 8.45E-09 | 6.64E-08 | 9.476392611 |
| 429 | NUDT7 | 0.581258686 | 8.482708495 | 5.969496381 | 9.45E-09 | 7.36E-08 | 9.36865121 |
| 430 | GUCA1C | 0.956497705 | 5.41388976 | 5.932321063 | 1.15E-08 | 8.83E-08 | 9.178721516 |
| 431 | CYBB | -0.554363956 | 7.695318935 | -5.914423139 | 1.26E-08 | 9.63E-08 | 9.087585987 |
| 432 | CA3 | 0.502563959 | 5.23538004 | 5.879226829 | 1.52E-08 | 1.15E-07 | 8.908949577 |
| 433 | TMEM182 | 0.521129822 | 10.00171371 | 5.82882737 | 1.97E-08 | 1.45E-07 | 8.654501158 |
| 434 | MID1 | 0.524558686 | 7.648242565 | 5.828429805 | 1.98E-08 | 1.46E-07 | 8.652500347 |
| 435 | DOK5 | 0.640530436 | 6.974988328 | 5.808274523 | 2.19E-08 | 1.61E-07 | 8.551196417 |
| 436 | C1orf118 | -0.583565879 | 5.71281929 | -5.773699634 | 2.62E-08 | 1.90E-07 | 8.37801602 |
| 437 | CFH | 0.501107857 | 7.733220302 | 5.741696802 | 3.09E-08 | 2.21E-07 | 8.218396132 |
| 438 | GZMK | 0.518608399 | 3.806258771 | 5.730498431 | 3.27E-08 | 2.34E-07 | 8.162696677 |
| 439 | C5orf4 | 0.508110356 | 8.201261485 | 5.723685022 | 3.39E-08 | 2.42E-07 | 8.128846818 |
| 440 | CNTN3 | -0.554081708 | 4.884500445 | -5.713761768 | 3.57E-08 | 2.53E-07 | 8.079600091 |
| 441 | FAP | 0.572352639 | 4.173718707 | 5.704833967 | 3.73E-08 | 2.64E-07 | 8.03534757 |
| 442 | FBXO40 | 0.663677037 | 9.294953739 | 5.679316553 | 4.25E-08 | 2.98E-07 | 7.909147866 |
| 443 | FCGR2A | -0.504727189 | 5.894365913 | -5.642613255 | 5.13E-08 | 3.54E-07 | 7.728364139 |
| 444 | EPHA7 | 0.546984278 | 5.915590217 | 5.633476786 | 5.37E-08 | 3.69E-07 | 7.683497745 |
| 445 | LOH3CR2A | -0.694025488 | 4.935364499 | -5.58868025 | 6.73E-08 | 4.56E-07 | 7.464302047 |
| 446 | S100A12 | -0.746264461 | 4.59288557 | -5.588061605 | 6.76E-08 | 4.58E-07 | 7.461284099 |
| 447 | MIR27B | 0.619318888 | 6.731070813 | 5.499800544 | 1.05E-07 | 6.88E-07 | 7.03329589 |
| 448 | CHRDL1 | 0.702673064 | 5.867030518 | 5.48588602 | 1.13E-07 | 7.33E-07 | 6.966292214 |
| 449 | LDLR | -0.523710418 | 7.151686028 | -5.470148971 | 1.22E-07 | 7.88E-07 | 6.890667383 |
| 450 | AQP4 | -0.778559012 | 5.749216321 | -5.40626402 | 1.67E-07 | 1.05E-06 | 6.58536365 |
| 451 | FBXO32 | 0.50701308 | 9.520917112 | 5.365100195 | 2.05E-07 | 1.27E-06 | 6.390094613 |
| 452 | ASB14 | 0.878104377 | 7.487482037 | 5.359545195 | 2.10E-07 | 1.30E-06 | 6.3638309 |
| 453 | VAT1L | 0.556963432 | 6.596119867 | 5.342827001 | 2.28E-07 | 1.41E-06 | 6.284914287 |
| 454 | CHI3L1 | -0.683297706 | 5.122355323 | -5.320495546 | 2.54E-07 | 1.56E-06 | 6.179796585 |
| 455 | GABRA4 | 0.523931702 | 7.023343859 | 5.307417673 | 2.71E-07 | 1.65E-06 | 6.118394415 |
| 456 | IRX6 | 0.514249117 | 6.672216256 | 5.304506609 | 2.75E-07 | 1.67E-06 | 6.10474248 |
| 457 | EDIL3 | 0.663049135 | 7.882768216 | 5.217135582 | 4.19E-07 | 2.47E-06 | 5.697701032 |
| 458 | FMO5 | -0.581786487 | 4.463960236 | -5.213400584 | 4.27E-07 | 2.51E-06 | 5.680417536 |
| 459 | LEPREL1 | 0.601651332 | 5.926163578 | 5.09044015 | 7.66E-07 | 4.29E-06 | 5.11683465 |
| 460 | HBA2 | 0.903410264 | 8.278940264 | 5.043161466 | 9.57E-07 | 5.29E-06 | 4.902952654 |
| 461 | HBA1 | 0.903410264 | 8.278940264 | 5.043161466 | 9.57E-07 | 5.29E-06 | 4.902952654 |
| 462 | SERPINE2 | 0.612721212 | 7.345031589 | 4.950777236 | 1.47E-06 | 7.84E-06 | 4.489590348 |
| 463 | POSTN | 0.813428986 | 8.0100469 | 4.948367177 | 1.49E-06 | 7.92E-06 | 4.478888273 |
| 464 | ANGPTL4 | -0.546238824 | 5.26618575 | -4.736228929 | 3.90E-06 | 1.92E-05 | 3.55331707 |
| 465 | SNORA42 | 0.520679528 | 8.259824961 | 4.729930741 | 4.01E-06 | 1.97E-05 | 3.526339649 |
| 466 | FOS | -0.631569448 | 6.950963901 | -4.706277599 | 4.46E-06 | 2.17E-05 | 3.425285827 |
| 467 | ADH1B | -0.629078309 | 7.672164445 | -4.697307483 | 4.64E-06 | 2.25E-05 | 3.387070663 |
| 468 | NR4A2 | -0.608046338 | 5.400824025 | -4.677364188 | 5.07E-06 | 2.44E-05 | 3.302320118 |
| 469 | PDE8B | 0.610534073 | 5.930088523 | 4.611492642 | 6.78E-06 | 3.19E-05 | 3.02449416 |
| 470 | HMGCS2 | -1.016169688 | 6.587302081 | -4.508959854 | 1.06E-05 | 4.82E-05 | 2.598507868 |
| 471 | FCGR1A | -0.581289699 | 4.748071866 | -4.452962998 | 1.35E-05 | 6.02E-05 | 2.369216029 |
| 472 | DHRS7C | -0.639519011 | 5.934382443 | -4.317425515 | 2.39E-05 | 0.00010145 | 1.824153742 |
| 473 | CXCL10 | 0.647765617 | 3.771345127 | 4.226295619 | 3.48E-05 | 0.000143241 | 1.465662783 |
| 474 | RNU4-2 | 0.795386349 | 6.832734327 | 4.127542215 | 5.21E-05 | 0.000206161 | 1.084523727 |
| 475 | TTTY10 | 0.78483613 | 5.125376225 | 4.127527804 | 5.21E-05 | 0.000206161 | 1.084468668 |
| 476 | TGFB2 | 0.593751972 | 7.341671445 | 3.948033584 | 0.000106075 | 0.000394498 | 0.411537943 |
| 477 | USP9Y | 0.984644211 | 6.295564454 | 3.787818311 | 0.000196155 | 0.000690407 | -0.167164482 |
| 478 | SLN | 0.556120652 | 5.695512953 | 3.707623701 | 0.000264865 | 0.00090771 | -0.448939073 |
| 479 | CCL18 | -0.551997087 | 6.42154325 | -3.651092161 | 0.000326337 | 0.001097069 | -0.644374738 |
| 480 | CPNE5 | 0.538097077 | 7.02083611 | 3.618152021 | 0.000368116 | 0.001223468 | -0.757027019 |
| 481 | TNC | -0.624504943 | 6.511137505 | -3.151643899 | 0.001849612 | 0.005224102 | -2.253832705 |
| 482 | VTRNA1-1 | 0.507315649 | 6.608798638 | 3.053869467 | 0.002537823 | 0.006910603 | -2.543766509 |
| 483 | CD24 | 0.531121444 | 4.530374697 | 3.039228201 | 0.002659163 | 0.007208069 | -2.586461385 |
| 484 | KDM5D | 0.674200713 | 6.028252343 | 2.991240249 | 0.003095267 | 0.008239131 | -2.72507429 |
| 485 | EIF1AY | 1.161797641 | 7.926874284 | 2.957765226 | 0.003437362 | 0.009054536 | -2.820563058 |
| 486 | CYorf15A | 0.554757645 | 5.221891548 | 2.856757628 | 0.004690301 | 0.011962897 | -3.102666059 |
| 487 | DDX3Y | 0.770464446 | 6.099170004 | 2.838879793 | 0.004951248 | 0.01256359 | -3.151650355 |
| 488 | UTY | 0.751467952 | 6.053040876 | 2.808891847 | 0.005418786 | 0.013606756 | -3.233174003 |
| 489 | RPS4Y1 | 0.659262069 | 6.020011054 | 2.56809283 | 0.010888494 | 0.025273385 | -3.858435069 |
