# Supplementary material for: Screening for immune-related biomarkers associated with myasthenia gravis and dilated cardiomyopathy based on bioinformatics analysis and machine learning
Source: Heliyon. 2024 Mar 20;10(7):e28446. doi: 10.1016/j.heliyon.2024.e28446 (PMC10988011; doi:10.1016/j.heliyon.2024.e28446)
Supplement: Multimedia component 3 [file mmc3.docx]

Table 3 Module genes of WGCNA in GSE85452（Salmon Module）

| NO. | Gene Symbol | NO. | Gene Symbol | NO. | Gene Symbol |
| --- | --- | --- | --- | --- | --- |
| 1 | FPR2 | 41 | RNMTL1 | 81 | MKNK1 |
| 2 | C3AR1 | 42 | NAPB | 82 | C1orf128 |
| 3 | IRS2 | 43 | ZEB2 | 83 | C6orf136 |
| 4 | MX2 | 44 | ANAPC1 | 84 | WIPF1 |
| 5 | SLC30A1 | 45 | RWDD1 | 85 | TRIAP1 |
| 6 | ZNF296 | 46 | TLR4 | 86 | SF3A3 |
| 7 | DUSP12 | 47 | NPTN | 87 | SDCBP |
| 8 | CBX6 | 48 | LOC652624 | 88 | SPAG7 |
| 9 | YOD1 | 49 | CD93 | 89 | CLCN6 |
| 10 | LOC389322 | 50 | ZNF581 | 90 | HCCS |
| 11 | LOC730820 | 51 | SNORD36C | 91 | SFRS16 |
| 12 | LMNB1 | 52 | BAZ2B | 92 | LOC388796 |
| 13 | RBM47 | 53 | C1orf54 | 93 | LAT2 |
| 14 | RNF149 | 54 | ITCH | 94 | ZRANB2 |
| 15 | RIN2 | 55 | MED10 | 95 | PEMT |
| 16 | TIGA1 | 56 | CWC15 | 96 | SP3 |
| 17 | LPP | 57 | FKBP5 | 97 | MRPS30 |
| 18 | MOBKL1B | 58 | HMG20B | 98 | PARP6 |
| 19 | CLP1 | 59 | CHIC2 | 99 | ACOX1 |
| 20 | FASTKD5 | 60 | USP39 | 100 | BCOR |
| 21 | TMED10 | 61 | PHF13 | 101 | WDFY1 |
| 22 | YWHAG | 62 | FAM39DP | 102 | CAMLG |
| 23 | C16orf87 | 63 | AGFG1 | 103 | MORF4L1 |
| 24 | ZDHHC1 | 64 | MOBK1B | 104 | ARIH2 |
| 25 | VAMP1 | 65 | RABGGTB | 105 | SEC22B |
| 26 | YIPF4 | 66 | ZNF211 | 106 | COIL |
| 27 | LHFPL2 | 67 | CCDC45 | 107 | ZNF277 |
| 28 | NECAP1 | 68 | SON | 108 | IL15 |
| 29 | ERICH1 | 69 | ILK | 109 | TAF6L |
| 30 | SEMA4D | 70 | C22orf32 | 110 | FOLR2 |
| 31 | BTG1 | 71 | SERPINB8 | 111 | LGTN |
| 32 | MAP2K1 | 72 | OSBPL11 | 112 | MAN2A2 |
| 33 | MS4A4A | 73 | ZFAND5 | 113 | CLDND2 |
| 34 | HAVCR2 | 74 | LOC730455 | 114 | KTI12 |
| 35 | MAD2L1BP | 75 | SF3B2 |  |  |
| 36 | FEM1B | 76 | BAG4 |  |  |
| 37 | VEGFB | 77 | LOC145853 |  |  |
| 38 | C12orf47 | 78 | C2orf24 |  |  |
| 39 | ZFAND2A | 79 | COX6C |  |  |
| 40 | SNORA12 | 80 | TMEM154 |  |  |
