# Supplementary material for: Screening for immune-related biomarkers associated with myasthenia gravis and dilated cardiomyopathy based on bioinformatics analysis and machine learning
Source: Heliyon. 2024 Mar 20;10(7):e28446. doi: 10.1016/j.heliyon.2024.e28446 (PMC10988011; doi:10.1016/j.heliyon.2024.e28446)
Supplement: Multimedia component 4 [file mmc4.docx]

Table 4 Module genes of WGCNA in GSE57338（Cyan Module）

| NO. | Gene Symbol | NO. | Gene Symbol | NO. | Gene Symbol |
| --- | --- | --- | --- | --- | --- |
| 1 | HBB | 851 | TET1 | 1701 | C17orf57 |
| 2 | ASPN | 852 | SLC1A2 | 1702 | EMILIN1 |
| 3 | UGT2B4 | 853 | RNPC3 | 1703 | SUN2 |
| 4 | NEB | 854 | WDR31 | 1704 | C6orf225 |
| 5 | GUCA1C | 855 | MAP1B | 1705 | LOC387790 |
| 6 | F5 | 856 | RCAN1 | 1706 | ME1 |
| 7 | POSTN | 857 | CES4A | 1707 | INPP5J |
| 8 | MYH6 | 858 | SLC16A4 | 1708 | SNX31 |
| 9 | TNC | 859 | SERPINB1 | 1709 | SPHK1 |
| 10 | FCN3 | 860 | UGDH | 1710 | SCN5A |
| 11 | ATRNL1 | 861 | CES1P1 | 1711 | LTBP3 |
| 12 | MXRA5 | 862 | DLL1 | 1712 | TMEM97 |
| 13 | MIR208A | 863 | AMICA1 | 1713 | REEP2 |
| 14 | CPNE5 | 864 | PCDHB13 | 1714 | MTERFD3 |
| 15 | SFRP4 | 865 | MAP2K1 | 1715 | FBXL22 |
| 16 | SERPINE1 | 866 | FLNA | 1716 | FZD1 |
| 17 | HLA-DQB1 | 867 | ZNF385D | 1717 | NGFRAP1 |
| 18 | SGPP2 | 868 | ENO1 | 1718 | THSD1P1 |
| 19 | HLA-DRB5 | 869 | IVNS1ABP | 1719 | LOC653113 |
| 20 | DLK1 | 870 | PTPN3 | 1720 | SRGAP1 |
| 21 | LUM | 871 | ATP1A1 | 1721 | SLC25A19 |
| 22 | PI16 | 872 | DUSP5 | 1722 | LCNL1 |
| 23 | EDIL3 | 873 | SEMA6B | 1723 | GRIN3A |
| 24 | COL14A1 | 874 | BEST1 | 1724 | HRK |
| 25 | C6 | 875 | SNORD116-28 | 1725 | KLC2 |
| 26 | MGC39372 | 876 | GUCY1A3 | 1726 | FLJ44606 |
| 27 | FLJ34503 | 877 | SGCG | 1727 | ZNF772 |
| 28 | FRZB | 878 | CPT2 | 1728 | TXNL4B |
| 29 | CP | 879 | C8orf45 | 1729 | HLA-DOA |
| 30 | LTBP2 | 880 | CA3 | 1730 | TIAM1 |
| 31 | SHISA3 | 881 | CDNF | 1731 | INHBB |
| 32 | ASB14 | 882 | C22orf25 | 1732 | KLF7 |
| 33 | DSC1 | 883 | NAV3 | 1733 | NCALD |
| 34 | HAS2 | 884 | MORN4 | 1734 | SREBF1 |
| 35 | HAPLN1 | 885 | FITM1 | 1735 | ENG |
| 36 | IFI44L | 886 | DKFZP779L1853 | 1736 | SPRED1 |
| 37 | CNN1 | 887 | CA5B | 1737 | KIAA1324L |
| 38 | OGN | 888 | FBLN2 | 1738 | ITPR1 |
| 39 | NAP1L3 | 889 | ZFP14 | 1739 | ARMC2 |
| 40 | SMOC2 | 890 | FAM47E | 1740 | TUBBP5 |
| 41 | ANKRD2 | 891 | SOHLH2 | 1741 | TCEANC |
| 42 | SFRP1 | 892 | GLIPR1L2 | 1742 | CX3CL1 |
| 43 | C5orf23 | 893 | LOC147727 | 1743 | F2RL3 |
| 44 | MYOT | 894 | CDH6 | 1744 | MBLAC2 |
| 45 | LOC284757 | 895 | RAB30 | 1745 | LRRK2 |
| 46 | FGF18 | 896 | OPN3 | 1746 | ADAM23 |
| 47 | DIRAS3 | 897 | OAS1 | 1747 | TTC39C |
| 48 | METTL7B | 898 | OR4F16 | 1748 | GLA |
| 49 | PDE5A | 899 | ENPEP | 1749 | ITPK1 |
| 50 | GGTA1 | 900 | HTRA3 | 1750 | ZNF827 |
| 51 | PDE1A | 901 | ACOT11 | 1751 | PMS2P1 |
| 52 | NOX4 | 902 | MTFP1 | 1752 | CBX7 |
| 53 | VCAN | 903 | MDH1B | 1753 | MIR23A |
| 54 | C1orf105 | 904 | XKR4 | 1754 | RHOJ |
| 55 | FAM180A | 905 | LTBR | 1755 | TANC1 |
| 56 | LOC121952 | 906 | EIF4EBP1 | 1756 | PIK3R6 |
| 57 | PHLDA1 | 907 | ACSF2 | 1757 | OMA1 |
| 58 | NRK | 908 | GABRB3 | 1758 | PELI1 |
| 59 | NPR3 | 909 | GBP3 | 1759 | ZBTB37 |
| 60 | MIR21 | 910 | DGKD | 1760 | SNORA70 |
| 61 | FMOD | 911 | ZNF724P | 1761 | SLC27A4 |
| 62 | OMD | 912 | PRKAR2B | 1762 | RCC1 |
| 63 | THBS4 | 913 | CIRBP | 1763 | TSPAN13 |
| 64 | C13orf33 | 914 | ALDH7A1 | 1764 | OSGEPL1 |
| 65 | AGXT2L1 | 915 | MYH9 | 1765 | XRCC2 |
| 66 | SNORD115-11 | 916 | SMC2 | 1766 | TARBP1 |
| 67 | LCN6 | 917 | GPRASP2 | 1767 | SH3BGRL3 |
| 68 | SNORD115-26 | 918 | TFPI2 | 1768 | ZNF749 |
| 69 | CYP1B1 | 919 | SNORD30 | 1769 | NPAS2 |
| 70 | MNS1 | 920 | PRTFDC1 | 1770 | RP2 |
| 71 | CRIP3 | 921 | TUBB6 | 1771 | SLC43A3 |
| 72 | THBS1 | 922 | PM20D1 | 1772 | ZFAND2A |
| 73 | MME | 923 | C1orf88 | 1773 | THOC5 |
| 74 | FNDC1 | 924 | CEACAM1 | 1774 | TFE3 |
| 75 | SLC27A6 | 925 | LOC445341 | 1775 | PGBD5 |
| 76 | IRX6 | 926 | CPN2 | 1776 | RPS6KA6 |
| 77 | FLJ34690 | 927 | C1QTNF2 | 1777 | SPSB1 |
| 78 | C1QTNF1 | 928 | ALDH5A1 | 1778 | KCNIP3 |
| 79 | EPHA7 | 929 | NCRNA00268 | 1779 | FZD8 |
| 80 | MOXD1 | 930 | HMGCLL1 | 1780 | NAAA |
| 81 | TMEM71 | 931 | BTBD8 | 1781 | HTATIP2 |
| 82 | AQP3 | 932 | PCDHB14 | 1782 | SOD3 |
| 83 | WNT9A | 933 | ACSS1 | 1783 | OSR1 |
| 84 | TUBA3D | 934 | BTN3A3 | 1784 | PARK2 |
| 85 | BTG2 | 935 | LDLRAD3 | 1785 | ECHDC2 |
| 86 | TUBA3E | 936 | CAB39L | 1786 | C14orf28 |
| 87 | FREM1 | 937 | PLCB4 | 1787 | KGFLP2 |
| 88 | HLA-DQA1 | 938 | D4S234E | 1788 | ZCWPW2 |
| 89 | CNTN3 | 939 | SYNPO2L | 1789 | IFT80 |
| 90 | CHL1 | 940 | PXDN | 1790 | TINAGL1 |
| 91 | GRIK2 | 941 | ZNF711 | 1791 | ZFAND5 |
| 92 | ENAM | 942 | MEIS1 | 1792 | FGGY |
| 93 | SNORD115-12 | 943 | HERC5 | 1793 | SNORD96A |
| 94 | RASL11B | 944 | FLJ14107 | 1794 | P2RX6 |
| 95 | C4orf29 | 945 | ATP1B4 | 1795 | PTGDS |
| 96 | PROS1 | 946 | SUSD1 | 1796 | CNTN4 |
| 97 | UCHL1 | 947 | SNORD4B | 1797 | CHST6 |
| 98 | VAT1L | 948 | SLC47A1 | 1798 | FAM110B |
| 99 | PLCE1 | 949 | PAIP2B | 1799 | HIST2H2AB |
| 100 | SCN2B | 950 | TAPBPL | 1800 | ELOVL5 |
| 101 | ZMYND17 | 951 | B3GALNT1 | 1801 | PRELID1 |
| 102 | IGSF10 | 952 | ATP6V1G2 | 1802 | ACER3 |
| 103 | NCRNA00189 | 953 | MPI | 1803 | DET1 |
| 104 | ZNF676 | 954 | ARID5B | 1804 | FXYD5 |
| 105 | PTN | 955 | YPEL2 | 1805 | OXCT1 |
| 106 | TIMP1 | 956 | FKBP10 | 1806 | VRK1 |
| 107 | IRF6 | 957 | SLFN12L | 1807 | DDX60L |
| 108 | LRRC10 | 958 | AFF3 | 1808 | ZNF593 |
| 109 | ANGPTL4 | 959 | NOS3 | 1809 | CTNNA3 |
| 110 | MFAP4 | 960 | CKB | 1810 | TSPAN32 |
| 111 | DPT | 961 | C12orf24 | 1811 | ZNF563 |
| 112 | CLIC6 | 962 | TEAD2 | 1812 | RIPK2 |
| 113 | LOC1720 | 963 | FHL3 | 1813 | ENTPD1 |
| 114 | G0S2 | 964 | ZMYND12 | 1814 | SEMA7A |
| 115 | ITGA2 | 965 | PTPRU | 1815 | SEZ6L |
| 116 | LPHN3 | 966 | STC2 | 1816 | QRSL1 |
| 117 | C5orf13 | 967 | TTC18 | 1817 | TMEM86A |
| 118 | C1orf51 | 968 | DPY19L2 | 1818 | CACNA2D1 |
| 119 | PLEKHH2 | 969 | THBS3 | 1819 | ENTPD6 |
| 120 | FAM69C | 970 | AIG1 | 1820 | ZNF846 |
| 121 | HSPA2 | 971 | LPIN1 | 1821 | C21orf34 |
| 122 | MATN2 | 972 | SSPN | 1822 | C1orf110 |
| 123 | MIRLET7C | 973 | PPP1R3B | 1823 | GPAT2 |
| 124 | ASB15 | 974 | C6orf163 | 1824 | SLC35F2 |
| 125 | DHFR | 975 | TDRD1 | 1825 | UBE2Q2P1 |
| 126 | GLP1R | 976 | SRR | 1826 | FOSL1 |
| 127 | CRYM | 977 | SNED1 | 1827 | INTU |
| 128 | ECM2 | 978 | RGS11 | 1828 | ACTG1 |
| 129 | ITIH5 | 979 | EHHADH | 1829 | DYNC2H1 |
| 130 | NPTX2 | 980 | C8orf49 | 1830 | SAP30 |
| 131 | PKHD1L1 | 981 | PLAU | 1831 | PCK2 |
| 132 | FAP | 982 | ZNF578 | 1832 | MIR27A |
| 133 | SLC6A1 | 983 | YPEL1 | 1833 | SORBS2 |
| 134 | SVEP1 | 984 | CCND2 | 1834 | RNF150 |
| 135 | ERAP2 | 985 | CCDC93 | 1835 | TMEM133 |
| 136 | DIO2 | 986 | SMAD7 | 1836 | PDPN |
| 137 | C3orf43 | 987 | GLRB | 1837 | LAMC2 |
| 138 | CFH | 988 | MICA | 1838 | MTMR4 |
| 139 | LOC389834 | 989 | TAS2R50 | 1839 | GPX7 |
| 140 | GABRA4 | 990 | PYGB | 1840 | PCYOX1 |
| 141 | ACE2 | 991 | H1F0 | 1841 | VASP |
| 142 | FAM46B | 992 | HDAC9 | 1842 | GNG12 |
| 143 | ALS2CR11 | 993 | SCN4B | 1843 | TMEM165 |
| 144 | HOPX | 994 | DUSP19 | 1844 | ZNF493 |
| 145 | ANGPT2 | 995 | NTM | 1845 | ALDH1A3 |
| 146 | MID1 | 996 | PCSK6 | 1846 | LRMP |
| 147 | GRIA3 | 997 | C21orf29 | 1847 | ZNF233 |
| 148 | RORB | 998 | ERI2 | 1848 | PPM1J |
| 149 | CXCL10 | 999 | C1orf183 | 1849 | MRPS12 |
| 150 | PER3 | 1000 | TPPP3 | 1850 | LAMB1 |
| 151 | F2RL2 | 1001 | C11orf71 | 1851 | PTGIR |
| 152 | SLC16A9 | 1002 | KITLG | 1852 | MAGI2 |
| 153 | LRRC14B | 1003 | MIR181B2 | 1853 | FKBP4 |
| 154 | ARG2 | 1004 | TLR3 | 1854 | LRRC2 |
| 155 | TUBA1C | 1005 | CADPS2 | 1855 | LRP10 |
| 156 | BEX1 | 1006 | C8orf37 | 1856 | TMEM99 |
| 157 | GJA5 | 1007 | RFESD | 1857 | ZNF334 |
| 158 | SERPINB8 | 1008 | SPATA17 | 1858 | CYP2U1 |
| 159 | AZGP1 | 1009 | NSDHL | 1859 | TYRP1 |
| 160 | CCDC113 | 1010 | HSPC159 | 1860 | FRY |
| 161 | SLC19A2 | 1011 | TMEM43 | 1861 | NRP1 |
| 162 | LRRC17 | 1012 | ACTN1 | 1862 | NHLRC3 |
| 163 | FLJ36840 | 1013 | SRGAP3 | 1863 | KIAA0427 |
| 164 | SNORD116-21 | 1014 | TAS2R31 | 1864 | C11orf75 |
| 165 | ATP1B3 | 1015 | C1orf175 | 1865 | GPR125 |
| 166 | ISLR | 1016 | ARHGDIG | 1866 | KIF3A |
| 167 | TFRC | 1017 | ANXA2 | 1867 | C14orf180 |
| 168 | SNORD115-42 | 1018 | ZNF404 | 1868 | LOC399744 |
| 169 | PCDH20 | 1019 | LOC283174 | 1869 | TMEM189 |
| 170 | CNTNAP3 | 1020 | HEY1 | 1870 | FMO1 |
| 171 | B3GALT2 | 1021 | PAPPA | 1871 | TUBG1 |
| 172 | MIR15A | 1022 | ATP6V0E2 | 1872 | NUDT5 |
| 173 | CSDC2 | 1023 | MYH7B | 1873 | RUNX1T1 |
| 174 | MIR30E | 1024 | MFI2 | 1874 | IL6R |
| 175 | C5orf46 | 1025 | MAL | 1875 | TNFAIP8L3 |
| 176 | EDN1 | 1026 | WARS | 1876 | SLC35F1 |
| 177 | KLHL38 | 1027 | CD97 | 1877 | CCRL1 |
| 178 | EDA2R | 1028 | EHD3 | 1878 | C1D |
| 179 | CREB5 | 1029 | HIST1H1E | 1879 | NUDT13 |
| 180 | MIR27B | 1030 | TRPC4 | 1880 | PTP4A2 |
| 181 | LOC150622 | 1031 | SLC13A4 | 1881 | CPVL |
| 182 | SNORD36B | 1032 | IPP | 1882 | GABPB2 |
| 183 | C1orf168 | 1033 | LOC400986 | 1883 | PTGR2 |
| 184 | SNCA | 1034 | ALDH1L2 | 1884 | SLC5A6 |
| 185 | GLT8D2 | 1035 | FSIP2 | 1885 | TTC30A |
| 186 | OLFML1 | 1036 | PARD6B | 1886 | PIGF |
| 187 | ZDHHC2 | 1037 | PLXNA4 | 1887 | NOTCH1 |
| 188 | CCDC144A | 1038 | MAPRE3 | 1888 | NR2F2 |
| 189 | ITGB6 | 1039 | OSBP2 | 1889 | FAM178A |
| 190 | DHCR24 | 1040 | DNER | 1890 | FAM124A |
| 191 | PRICKLE1 | 1041 | PEX12 | 1891 | CYB5R2 |
| 192 | GSTM5 | 1042 | CDR1 | 1892 | TP53INP1 |
| 193 | SNORD115-1 | 1043 | C6orf99 | 1893 | CLCN5 |
| 194 | XRRA1 | 1044 | LDHA | 1894 | PFDN4 |
| 195 | FAM81B | 1045 | TNIK | 1895 | SERINC2 |
| 196 | RXRG | 1046 | PEG10 | 1896 | C9orf23 |
| 197 | MMP16 | 1047 | C5orf54 | 1897 | FGF2 |
| 198 | SULF1 | 1048 | SLFN5 | 1898 | ELK3 |
| 199 | PAMR1 | 1049 | SNORA56 | 1899 | TTC12 |
| 200 | PI15 | 1050 | CADM1 | 1900 | TGM2 |
| 201 | KLHL13 | 1051 | RPS28 | 1901 | C14orf45 |
| 202 | RGS2 | 1052 | PCOLCE2 | 1902 | MYADM |
| 203 | TNNI1 | 1053 | PLEKHG1 | 1903 | PGAP1 |
| 204 | SLC44A5 | 1054 | PCCB | 1904 | BEX4 |
| 205 | KLKB1 | 1055 | PHACTR1 | 1905 | SLC16A13 |
| 206 | TM7SF2 | 1056 | CYP27A1 | 1906 | ALKBH7 |
| 207 | FMO3 | 1057 | MLYCD | 1907 | PSMB9 |
| 208 | C1orf118 | 1058 | HCP5 | 1908 | ZNF713 |
| 209 | TNNT1 | 1059 | CD163L1 | 1909 | SLC12A7 |
| 210 | FSD2 | 1060 | MTHFD1L | 1910 | GRAPL |
| 211 | CGNL1 | 1061 | PKDCC | 1911 | HEXA |
| 212 | MIR23B | 1062 | BDH2 | 1912 | CDKN2B |
| 213 | FADS2 | 1063 | RPS24 | 1913 | MCM8 |
| 214 | GNMT | 1064 | OSBPL3 | 1914 | BCKDHB |
| 215 | C10orf110 | 1065 | C10orf78 | 1915 | CSPG4P1Y |
| 216 | SNAP47 | 1066 | ECT2 | 1916 | ATG14 |
| 217 | GABRB1 | 1067 | NPC1 | 1917 | LOC100130581 |
| 218 | PTGIS | 1068 | CD109 | 1918 | RAD51L1 |
| 219 | MIR99A | 1069 | MIR32 | 1919 | TRIM69 |
| 220 | CCDC3 | 1070 | ORMDL2 | 1920 | UBR1 |
| 221 | COL12A1 | 1071 | ADCY5 | 1921 | HFM1 |
| 222 | ARRDC3 | 1072 | KCNJ2 | 1922 | TP53 |
| 223 | CYP2J2 | 1073 | STARD7 | 1923 | CD63 |
| 224 | TNNT3 | 1074 | LONRF3 | 1924 | NPIPL2 |
| 225 | FGF14 | 1075 | CLIC1 | 1925 | LONRF2 |
| 226 | SCUBE3 | 1076 | C21orf82 | 1926 | DUS1L |
| 227 | AEBP1 | 1077 | CHST11 | 1927 | HSD17B8 |
| 228 | KCNMB2 | 1078 | KGFLP1 | 1928 | C8orf40 |
| 229 | P2RX3 | 1079 | FABP2 | 1929 | HOXA2 |
| 230 | CTSK | 1080 | HSD17B3 | 1930 | KIAA1328 |
| 231 | MAPT | 1081 | C21orf119 | 1931 | MPV17L2 |
| 232 | CES1 | 1082 | MPDU1 | 1932 | CHRNB1 |
| 233 | SNORD115-6 | 1083 | COPZ2 | 1933 | PDGFB |
| 234 | CDKN1A | 1084 | PLIN3 | 1934 | TSFM |
| 235 | CADPS | 1085 | PARP14 | 1935 | CSRP1 |
| 236 | C6orf138 | 1086 | PDE3B | 1936 | SYT7 |
| 237 | MLLT11 | 1087 | THNSL1 | 1937 | GPR111 |
| 238 | HMCN1 | 1088 | DPF3 | 1938 | RNF207 |
| 239 | CMKLR1 | 1089 | FYCO1 | 1939 | LST-3TM12 |
| 240 | HPGDS | 1090 | ADAMTSL3 | 1940 | FASTKD1 |
| 241 | VGLL3 | 1091 | ALDOC | 1941 | DNM1 |
| 242 | GNA14 | 1092 | UST | 1942 | PECAM1 |
| 243 | DPYSL4 | 1093 | SLC31A2 | 1943 | PIGH |
| 244 | PIK3IP1 | 1094 | CCDC122 | 1944 | FGF5 |
| 245 | HK2 | 1095 | TES | 1945 | CFL1 |
| 246 | JAK2 | 1096 | GRIP2 | 1946 | UBTD1 |
| 247 | MAPK4 | 1097 | NFASC | 1947 | PARD6A |
| 248 | SH3GL2 | 1098 | AMACR | 1948 | BMP8B |
| 249 | ABCG2 | 1099 | PCP4 | 1949 | PCDHB9 |
| 250 | CDH19 | 1100 | KLHL30 | 1950 | RPS5 |
| 251 | SLC7A1 | 1101 | CHMP4C | 1951 | GSG1L |
| 252 | PLP2 | 1102 | PREX2 | 1952 | EDEM2 |
| 253 | GPR4 | 1103 | PRSS23 | 1953 | ABCA11P |
| 254 | C1QTNF9 | 1104 | PLEKHA6 | 1954 | TMEM178 |
| 255 | C20orf26 | 1105 | PPDPF | 1955 | ACTB |
| 256 | SDSL | 1106 | ASPA | 1956 | ZNF138 |
| 257 | PODN | 1107 | ZYX | 1957 | HERC4 |
| 258 | ITGBL1 | 1108 | ALS2CR8 | 1958 | KIAA0922 |
| 259 | C15orf59 | 1109 | CXorf36 | 1959 | SEC16B |
| 260 | FRMD4B | 1110 | ADAM12 | 1960 | NPR1 |
| 261 | LOC221442 | 1111 | ITGB1BP2 | 1961 | KLF6 |
| 262 | CRISPLD1 | 1112 | ALG3 | 1962 | MTCH1 |
| 263 | NPNT | 1113 | HMBS | 1963 | PTPN1 |
| 264 | GATSL1 | 1114 | AMOT | 1964 | C5orf35 |
| 265 | HLA-DMB | 1115 | PLA2G4C | 1965 | ARIH2 |
| 266 | ABCD2 | 1116 | RGN | 1966 | PRUNE |
| 267 | LOH3CR2A | 1117 | RGAG1 | 1967 | VWA5A |
| 268 | IL1R1 | 1118 | SLC30A3 | 1968 | SLC2A9 |
| 269 | TAGLN | 1119 | ACAD10 | 1969 | PRAF2 |
| 270 | SAMD12 | 1120 | SNORD36C | 1970 | ETS1 |
| 271 | CTNND2 | 1121 | TMEM143 | 1971 | RPS12 |
| 272 | HTR4 | 1122 | KIF13A | 1972 | HEG1 |
| 273 | P2RX5 | 1123 | ABHD1 | 1973 | AK5 |
| 274 | MUSTN1 | 1124 | TRANK1 | 1974 | EIF5AL1 |
| 275 | HFE2 | 1125 | P2RX7 | 1975 | NCRNA00247 |
| 276 | C20orf200 | 1126 | P2RY13 | 1976 | CLK1 |
| 277 | NT5E | 1127 | HRH2 | 1977 | C11orf1 |
| 278 | FIBIN | 1128 | CTSO | 1978 | FAM46A |
| 279 | ARNTL | 1129 | C13orf27 | 1979 | NXN |
| 280 | C1QTNF9B | 1130 | PITPNM1 | 1980 | MLKL |
| 281 | FMO4 | 1131 | ANO1 | 1981 | IFT74 |
| 282 | ADAMTS15 | 1132 | RCN1 | 1982 | AGPHD1 |
| 283 | FGFBP2 | 1133 | PRKCD | 1983 | TCEA3 |
| 284 | THNSL2 | 1134 | TBPL1 | 1984 | ACRC |
| 285 | GPSM2 | 1135 | LOC100128288 | 1985 | PPP1R3A |
| 286 | POF1B | 1136 | KBTBD12 | 1986 | C1orf31 |
| 287 | SLC36A4 | 1137 | PXMP4 | 1987 | DENND1B |
| 288 | LCN10 | 1138 | EEF1A1 | 1988 | PKIA |
| 289 | CYP4Z1 | 1139 | C9orf93 | 1989 | TRIM47 |
| 290 | PNP | 1140 | MLLT3 | 1990 | CHN1 |
| 291 | ABI3BP | 1141 | MTX2 | 1991 | SLC9A7 |
| 292 | RSAD2 | 1142 | CMBL | 1992 | TTC35 |
| 293 | IDO1 | 1143 | PPFIA4 | 1993 | TXNDC9 |
| 294 | HBEGF | 1144 | PTPN13 | 1994 | C1orf170 |
| 295 | PFKFB2 | 1145 | RASD2 | 1995 | ZNF83 |
| 296 | NAP1L2 | 1146 | PARVB | 1996 | GFOD1 |
| 297 | C10orf71 | 1147 | ADRA1A | 1997 | PRMT2 |
| 298 | MGC70870 | 1148 | NRIP2 | 1998 | HVCN1 |
| 299 | TPM3 | 1149 | JAM2 | 1999 | MASP1 |
| 300 | SCARNA9L | 1150 | TUBA8 | 2000 | ANKRA2 |
| 301 | HLA-DPA1 | 1151 | KCNMB1 | 2001 | PCYT2 |
| 302 | TCF21 | 1152 | C19orf18 | 2002 | ZNF630 |
| 303 | SIAE | 1153 | FAM167B | 2003 | KIAA1370 |
| 304 | ANKRD23 | 1154 | NRXN3 | 2004 | ALDH1B1 |
| 305 | SCUBE2 | 1155 | CNNM4 | 2005 | KDM1B |
| 306 | ALDH1A1 | 1156 | ARSK | 2006 | PIK3R3 |
| 307 | SNORD49B | 1157 | ISOC1 | 2007 | PRDX5 |
| 308 | CDHR3 | 1158 | FAHD1 | 2008 | MORN2 |
| 309 | SLC2A1 | 1159 | FRMD7 | 2009 | GSTA4 |
| 310 | TMEM176B | 1160 | CAND2 | 2010 | FGD4 |
| 311 | KBTBD10 | 1161 | DLEU1 | 2011 | ZNF799 |
| 312 | SGIP1 | 1162 | CXCL9 | 2012 | ANK1 |
| 313 | ENPP2 | 1163 | SLC39A14 | 2013 | CCDC136 |
| 314 | IFIT2 | 1164 | ZNF622 | 2014 | PRRT2 |
| 315 | SPON1 | 1165 | BMPR1B | 2015 | ACOX2 |
| 316 | NANOS1 | 1166 | GAS2 | 2016 | CALHM2 |
| 317 | BCL6 | 1167 | KBTBD3 | 2017 | ZNF429 |
| 318 | FLJ30064 | 1168 | FAM101B | 2018 | C2orf34 |
| 319 | CYSLTR2 | 1169 | C6orf145 | 2019 | HIST2H2AA3 |
| 320 | F2R | 1170 | CC2D2A | 2020 | JAG1 |
| 321 | ZMAT1 | 1171 | BCO2 | 2021 | CLTCL1 |
| 322 | MIR29C | 1172 | IGSF5 | 2022 | GLRX2 |
| 323 | PLVAP | 1173 | GPR56 | 2023 | KIAA1467 |
| 324 | KAL1 | 1174 | MPP6 | 2024 | FLJ38109 |
| 325 | SPHKAP | 1175 | PCDH9 | 2025 | TTC3 |
| 326 | EFCAB2 | 1176 | ILVBL | 2026 | SEC61A2 |
| 327 | PCDHB5 | 1177 | WEE1 | 2027 | HRC |
| 328 | F3 | 1178 | DYRK1B | 2028 | CCDC28A |
| 329 | ANKRD29 | 1179 | TTC39A | 2029 | SNHG12 |
| 330 | C16orf89 | 1180 | NBLA00301 | 2030 | MIR196A1 |
| 331 | TM4SF18 | 1181 | NAALADL2 | 2031 | BLID |
| 332 | SCN7A | 1182 | XG | 2032 | HSF4 |
| 333 | GARNL3 | 1183 | EGF | 2033 | TTC1 |
| 334 | ASB4 | 1184 | CRHR2 | 2034 | BRP44L |
| 335 | ITGA5 | 1185 | ST8SIA6 | 2035 | OR52N4 |
| 336 | STAT3 | 1186 | C12orf39 | 2036 | SPATA18 |
| 337 | GPRASP1 | 1187 | TMEM231 | 2037 | LYRM7 |
| 338 | TMEM140 | 1188 | RERGL | 2038 | SCRN3 |
| 339 | ZNF208 | 1189 | ZDHHC15 | 2039 | B3GNT7 |
| 340 | COL15A1 | 1190 | PVRL1 | 2040 | KIAA0226 |
| 341 | MIRLET7G | 1191 | DYNLT1 | 2041 | HMGN2 |
| 342 | DUSP13 | 1192 | ZDHHC16 | 2042 | NUBPL |
| 343 | PLAT | 1193 | C10orf54 | 2043 | URB1 |
| 344 | MID1IP1 | 1194 | NACC1 | 2044 | SFXN3 |
| 345 | TRDN | 1195 | TMEM195 | 2045 | ESAM |
| 346 | ABAT | 1196 | WNK2 | 2046 | SLC14A1 |
| 347 | SAT1 | 1197 | FBXO40 | 2047 | BBS4 |
| 348 | ENPP5 | 1198 | UBXN10 | 2048 | PPARGC1A |
| 349 | ETNK2 | 1199 | NKAPP1 | 2049 | EGLN1 |
| 350 | MAFIP | 1200 | ID4 | 2050 | RAP1GAP |
| 351 | KCNN3 | 1201 | ADHFE1 | 2051 | ZNRD1-AS |
| 352 | ELL2 | 1202 | SEMA5B | 2052 | KLF16 |
| 353 | KCNA4 | 1203 | ADPRHL1 | 2053 | ESRRA |
| 354 | ATAD2 | 1204 | NDUFB3 | 2054 | PET112L |
| 355 | MAP1A | 1205 | NLGN1 | 2055 | ANKRD42 |
| 356 | SNORD115-32 | 1206 | FNDC4 | 2056 | DOPEY1 |
| 357 | IFIT1 | 1207 | ARHGAP1 | 2057 | MRPS33 |
| 358 | IL32 | 1208 | HIST1H1C | 2058 | C14orf19 |
| 359 | ZNF204P | 1209 | THSD4 | 2059 | ORMDL3 |
| 360 | MEG3 | 1210 | ADAMTSL5 | 2060 | OTUD7B |
| 361 | HTR2B | 1211 | GFRA3 | 2061 | CLCN4 |
| 362 | CPXM2 | 1212 | RPP14 | 2062 | C14orf179 |
| 363 | FZD7 | 1213 | TTLL3 | 2063 | LOC151760 |
| 364 | MSC | 1214 | LRRCC1 | 2064 | LOC554249 |
| 365 | BBS12 | 1215 | RAMP2 | 2065 | GALM |
| 366 | SVOP | 1216 | ZBTB42 | 2066 | SOX9 |
| 367 | SNORD34 | 1217 | PNPLA7 | 2067 | TSPYL1 |
| 368 | FGF7 | 1218 | BID | 2068 | USP11 |
| 369 | ADM | 1219 | KCNJ11 | 2069 | PPP1R14C |
| 370 | PLXDC1 | 1220 | RAG1AP1 | 2070 | PLEKHG2 |
| 371 | HLA-DPB1 | 1221 | CRYZ | 2071 | NCDN |
| 372 | VSTM2L | 1222 | ABHD2 | 2072 | VEGFA |
| 373 | S1PR3 | 1223 | C11orf21 | 2073 | UACA |
| 374 | RGS7BP | 1224 | NQO2 | 2074 | UNC45B |
| 375 | NT5C1A | 1225 | BACH2 | 2075 | TEC |
| 376 | GLDN | 1226 | SOX6 | 2076 | NDUFS7 |
| 377 | MYOF | 1227 | VIPR2 | 2077 | OSTC |
| 378 | EPHX2 | 1228 | MCAM | 2078 | FAM129A |
| 379 | SNORD115-44 | 1229 | ANGPTL7 | 2079 | PTGR1 |
| 380 | EFEMP1 | 1230 | ARHGEF9 | 2080 | LOC149837 |
| 381 | SLCO5A1 | 1231 | PPIL6 | 2081 | UBE2H |
| 382 | FLJ43315 | 1232 | HHAT | 2082 | TESK1 |
| 383 | TSPYL2 | 1233 | LIN9 | 2083 | SLC26A9 |
| 384 | KCNT2 | 1234 | SLC44A3 | 2084 | MAML3 |
| 385 | XRCC4 | 1235 | PPIP5K2 | 2085 | SYT17 |
| 386 | TCN2 | 1236 | PPPDE2 | 2086 | SH3RF3 |
| 387 | SESN3 | 1237 | LOC100133299 | 2087 | SEMA6A |
| 388 | SLC2A12 | 1238 | DEGS1 | 2088 | C6orf162 |
| 389 | LAD1 | 1239 | KRT8 | 2089 | C5orf41 |
| 390 | FGF1 | 1240 | HIVEP3 | 2090 | DGCR6L |
| 391 | ERRFI1 | 1241 | ROBO1 | 2091 | C7orf63 |
| 392 | LRRC6 | 1242 | PION | 2092 | ZNF501 |
| 393 | GRB14 | 1243 | C2orf40 | 2093 | FKBP1A |
| 394 | NDUFAF1 | 1244 | SPON2 | 2094 | NAP1L5 |
| 395 | VSNL1 | 1245 | KCNK3 | 2095 | SNORA71C |
| 396 | CCL11 | 1246 | 11-Mar | 2096 | ACTR6 |
| 397 | CD248 | 1247 | PDE1B | 2097 | C6orf226 |
| 398 | C17orf108 | 1248 | SLC29A2 | 2098 | DACT3 |
| 399 | LPAR4 | 1249 | WIPF3 | 2099 | RPL3 |
| 400 | LIPH | 1250 | TSHZ2 | 2100 | C4orf33 |
| 401 | TNNI3K | 1251 | UQCRFS1 | 2101 | GPR64 |
| 402 | AGPAT9 | 1252 | PLEKHA2 | 2102 | MRS2 |
| 403 | SLC25A30 | 1253 | FHIT | 2103 | HSPC157 |
| 404 | FBXO32 | 1254 | FAM58A | 2104 | LOC100132005 |
| 405 | FABP6 | 1255 | LEFTY2 | 2105 | SUGT1P1 |
| 406 | SLC41A2 | 1256 | TIMP2 | 2106 | PDIA5 |
| 407 | MIRLET7A2 | 1257 | NR0B2 | 2107 | C16orf57 |
| 408 | ELOVL6 | 1258 | NUDT12 | 2108 | TLN2 |
| 409 | PRRX1 | 1259 | SEL1L3 | 2109 | CDK5 |
| 410 | MMP15 | 1260 | SPTBN5 | 2110 | ESRRB |
| 411 | NR1D2 | 1261 | PARP8 | 2111 | TRIM8 |
| 412 | CECR1 | 1262 | PHKA1 | 2112 | SLC5A3 |
| 413 | IFI6 | 1263 | ACOT2 | 2113 | ZNF323 |
| 414 | IGFBP5 | 1264 | FBXL2 | 2114 | ZNF667 |
| 415 | SH3TC2 | 1265 | LRRC48 | 2115 | MALL |
| 416 | PANX1 | 1266 | BCL9L | 2116 | ADH5 |
| 417 | ARRDC4 | 1267 | CACNA2D2 | 2117 | ASRGL1 |
| 418 | NUDT7 | 1268 | H19 | 2118 | C1orf220 |
| 419 | SLC7A2 | 1269 | AXL | 2119 | CLUAP1 |
| 420 | DSG1 | 1270 | TAS2R10 | 2120 | ZNF17 |
| 421 | NCRNA00260 | 1271 | PGAP2 | 2121 | ZNF33B |
| 422 | HLA-DRA | 1272 | SEMA6D | 2122 | RPN1 |
| 423 | MIR133A1 | 1273 | LOC100130713 | 2123 | DOT1L |
| 424 | RRP12 | 1274 | SULT1C4 | 2124 | DTX4 |
| 425 | MAPK10 | 1275 | ARMCX4 | 2125 | DLG2 |
| 426 | FNIP2 | 1276 | TAS2R19 | 2126 | FAM70B |
| 427 | LPCAT3 | 1277 | FHOD3 | 2127 | ERP27 |
| 428 | PACRG | 1278 | PDZD2 | 2128 | PHTF2 |
| 429 | PTPN20B | 1279 | EPB49 | 2129 | NPPC |
| 430 | DOK6 | 1280 | ZNF594 | 2130 | TEK |
| 431 | GRAMD1B | 1281 | NANS | 2131 | DTWD1 |
| 432 | LOC100049716 | 1282 | ARMCX2 | 2132 | RNF144A |
| 433 | ZNF844 | 1283 | SLC25A27 | 2133 | OLFML2A |
| 434 | PPM1K | 1284 | AMZ2P1 | 2134 | CDK18 |
| 435 | NES | 1285 | FGF11 | 2135 | MBOAT1 |
| 436 | SNORD5 | 1286 | APOL6 | 2136 | PDE3A |
| 437 | ALS2CR12 | 1287 | AEN | 2137 | RPL23AP53 |
| 438 | HMOX2 | 1288 | RICTOR | 2138 | CAP1 |
| 439 | PSD3 | 1289 | EPSTI1 | 2139 | RAB38 |
| 440 | GPNMB | 1290 | S100A10 | 2140 | SGSM1 |
| 441 | S100A11 | 1291 | OCIAD2 | 2141 | ATP1A3 |
| 442 | C6orf186 | 1292 | TRIM16 | 2142 | KLRAP1 |
| 443 | ITGB3BP | 1293 | GPR180 | 2143 | TP53I11 |
| 444 | MLF1 | 1294 | BRCA1 | 2144 | CTPS |
| 445 | ANGPTL1 | 1295 | GNPNAT1 | 2145 | SEMA3F |
| 446 | PTPN5 | 1296 | APOBEC3B | 2146 | P4HA2 |
| 447 | DPP4 | 1297 | ENO2 | 2147 | SRXN1 |
| 448 | MAEL | 1298 | ODZ3 | 2148 | TM4SF1 |
| 449 | COL8A1 | 1299 | GPCPD1 | 2149 | HNMT |
| 450 | HSPA4L | 1300 | C2orf63 | 2150 | ZNF14 |
| 451 | PDGFRB | 1301 | MRPL21 | 2151 | LANCL3 |
| 452 | AMY2A | 1302 | DDX58 | 2152 | ADSSL1 |
| 453 | HERC6 | 1303 | CAPS2 | 2153 | MIR30C2 |
| 454 | PTPN20A | 1304 | MTHFR | 2154 | TTC9 |
| 455 | HLA-DRB3 | 1305 | CNIH4 | 2155 | PDCD4 |
| 456 | GBP4 | 1306 | PCDHB16 | 2156 | C20orf96 |
| 457 | CFHR1 | 1307 | MLXIP | 2157 | SLC25A13 |
| 458 | BTN3A1 | 1308 | NCRNA00287 | 2158 | TUBE1 |
| 459 | PHKG1 | 1309 | MTFR1 | 2159 | GABARAPL3 |
| 460 | RPGR | 1310 | LOC100133315 | 2160 | ELFN1 |
| 461 | FAM13C | 1311 | OAS2 | 2161 | NR3C2 |
| 462 | RTN4 | 1312 | CNN2 | 2162 | RBPMS2 |
| 463 | ST8SIA5 | 1313 | SLC10A6 | 2163 | FAM162A |
| 464 | SUSD4 | 1314 | PARM1 | 2164 | ARHGAP23 |
| 465 | MIR181A2 | 1315 | HMGB2 | 2165 | PLOD2 |
| 466 | GRM1 | 1316 | HCN2 | 2166 | C15orf58 |
| 467 | C9orf4 | 1317 | CACNB1 | 2167 | FAM13B |
| 468 | LTBP4 | 1318 | PBLD | 2168 | FAM171B |
| 469 | DUSP27 | 1319 | ZMAT4 | 2169 | AP1S2 |
| 470 | FRMPD4 | 1320 | GRIN2A | 2170 | PLA2G15 |
| 471 | SNORD76 | 1321 | DOC2B | 2171 | S100A16 |
| 472 | AMY2B | 1322 | EFNB2 | 2172 | PLXNA3 |
| 473 | GAB3 | 1323 | ANKRD36B | 2173 | GALK1 |
| 474 | TSPAN9 | 1324 | SGCE | 2174 | AKAP8 |
| 475 | TXNRD1 | 1325 | CRY2 | 2175 | IPMK |
| 476 | SLIT2 | 1326 | NUDT4P1 | 2176 | ZC3H6 |
| 477 | GPR52 | 1327 | ZNF677 | 2177 | GPATCH4 |
| 478 | ZNF483 | 1328 | DDO | 2178 | PITPNM2 |
| 479 | STARD4 | 1329 | TMEM90B | 2179 | EEF1B2 |
| 480 | HLF | 1330 | SNRPB | 2180 | NIPSNAP3A |
| 481 | FADS3 | 1331 | SLFN13 | 2181 | PTBP1 |
| 482 | COLEC10 | 1332 | EFHC1 | 2182 | CCBP2 |
| 483 | LRRC32 | 1333 | C6orf115 | 2183 | SNTB1 |
| 484 | SRPX2 | 1334 | TMEM135 | 2184 | HMOX1 |
| 485 | ZNF536 | 1335 | EBF3 | 2185 | SSR3 |
| 486 | LOC51152 | 1336 | SELM | 2186 | FAM164C |
| 487 | ELOVL2 | 1337 | OAS3 | 2187 | RIMKLA |
| 488 | CRHBP | 1338 | CYFIP2 | 2188 | SLC4A7 |
| 489 | ASB2 | 1339 | SNORD115-20 | 2189 | SOX18 |
| 490 | CCND1 | 1340 | CDON | 2190 | SDC2 |
| 491 | CATSPER2P1 | 1341 | ADAMTS12 | 2191 | CHCHD7 |
| 492 | SERPINI1 | 1342 | PANK1 | 2192 | CLEC2D |
| 493 | NAMPT | 1343 | ZNF441 | 2193 | EPS8 |
| 494 | DCLK1 | 1344 | STX2 | 2194 | SNORD62A |
| 495 | HIST1H2AK | 1345 | B3GALTL | 2195 | ICAM2 |
| 496 | HOOK1 | 1346 | ICK | 2196 | SLC16A2 |
| 497 | LMCD1 | 1347 | NECAB1 | 2197 | N6AMT1 |
| 498 | KIAA1107 | 1348 | C13orf30 | 2198 | MFSD10 |
| 499 | ATP1B2 | 1349 | SIGLEC10 | 2199 | MAP3K3 |
| 500 | P2RY2 | 1350 | TMEM40 | 2200 | C7orf54 |
| 501 | SLC30A2 | 1351 | SH3RF1 | 2201 | COL6A1 |
| 502 | ALDH1L1 | 1352 | LIN7A | 2202 | DGKZ |
| 503 | RANBP17 | 1353 | GTF2IRD1 | 2203 | PPP2R3B |
| 504 | ADRBK2 | 1354 | MYOZ1 | 2204 | TMEM185A |
| 505 | SPTLC3 | 1355 | ABHD10 | 2205 | PPM1E |
| 506 | PTGFRN | 1356 | IVD | 2206 | UBASH3B |
| 507 | FOXP2 | 1357 | STK33 | 2207 | GAB2 |
| 508 | PLXDC2 | 1358 | MIR24-2 | 2208 | SESN1 |
| 509 | PDE11A | 1359 | GNG5 | 2209 | LOC100130691 |
| 510 | MUM1L1 | 1360 | KIAA0141 | 2210 | HSD11B1 |
| 511 | INMT | 1361 | PGCP | 2211 | DTNB |
| 512 | CENPV | 1362 | REXO4 | 2212 | NT5DC1 |
| 513 | BMP5 | 1363 | WFS1 | 2213 | C1QTNF3 |
| 514 | ALPL | 1364 | C9orf150 | 2214 | GP1BB |
| 515 | CXCL12 | 1365 | LHX6 | 2215 | ADCY6 |
| 516 | PRSS42 | 1366 | ATF7IP2 | 2216 | ESYT3 |
| 517 | MIR186 | 1367 | PTPRZ1 | 2217 | TMEM54 |
| 518 | MEOX2 | 1368 | MED12L | 2218 | TMEM164 |
| 519 | C13orf31 | 1369 | C21orf91 | 2219 | PTGER4 |
| 520 | CDK2AP2 | 1370 | FAM161A | 2220 | FLII |
| 521 | SNORD59B | 1371 | C14orf132 | 2221 | FAM78B |
| 522 | COMMD3 | 1372 | SPINK1 | 2222 | XYLT1 |
| 523 | ZDHHC9 | 1373 | HIPK2 | 2223 | FAM149B1 |
| 524 | CPE | 1374 | ATG10 | 2224 | ZNF257 |
| 525 | CDCA7L | 1375 | SMPDL3A | 2225 | MAOB |
| 526 | ADAMTSL1 | 1376 | GPRC5A | 2226 | HMGCL |
| 527 | FAM134B | 1377 | SPA17 | 2227 | HSPG2 |
| 528 | NALCN | 1378 | ST3GAL1 | 2228 | GNAI1 |
| 529 | OGDHL | 1379 | WWTR1 | 2229 | INPP1 |
| 530 | CCDC141 | 1380 | C11orf24 | 2230 | ZNF816 |
| 531 | APOA1 | 1381 | C1orf97 | 2231 | YEATS4 |
| 532 | LOC285456 | 1382 | EXOC6B | 2232 | ZDBF2 |
| 533 | LRRC16A | 1383 | MANEA | 2233 | TMEFF1 |
| 534 | USP31 | 1384 | CD93 | 2234 | HCCS |
| 535 | BMP4 | 1385 | COL4A2 | 2235 | FAM63A |
| 536 | C7orf58 | 1386 | CAV3 | 2236 | ZDHHC13 |
| 537 | CYP4V2 | 1387 | IL34 | 2237 | CHRNE |
| 538 | COMP | 1388 | TRPC1 | 2238 | BLOC1S3 |
| 539 | STK38L | 1389 | ODZ2 | 2239 | QPRT |
| 540 | LOC375010 | 1390 | LMOD3 | 2240 | FXYD6 |
| 541 | HSPB6 | 1391 | POT1 | 2241 | SOAT1 |
| 542 | SPINK5 | 1392 | HSDL2 | 2242 | SLC2A4 |
| 543 | XAF1 | 1393 | ENO3 | 2243 | LRRC49 |
| 544 | PCLO | 1394 | ACOT13 | 2244 | NFKBIB |
| 545 | SHC4 | 1395 | TUBB2C | 2245 | RORA |
| 546 | SCRN1 | 1396 | GNG7 | 2246 | ZNF514 |
| 547 | FURIN | 1397 | ZNF177 | 2247 | FOXK2 |
| 548 | SLC26A2 | 1398 | PPL | 2248 | ACAD11 |
| 549 | HLA-DRB4 | 1399 | DYNLL1 | 2249 | CDC14B |
| 550 | SLC40A1 | 1400 | RPL32 | 2250 | AGTPBP1 |
| 551 | RANBP3L | 1401 | TYMP | 2251 | DPAGT1 |
| 552 | CCDC147 | 1402 | RAB9B | 2252 | PAQR5 |
| 553 | CHAC1 | 1403 | KCNE1 | 2253 | TDP1 |
| 554 | FBLN1 | 1404 | TMEM104 | 2254 | TDRD6 |
| 555 | SLC1A3 | 1405 | RBM3 | 2255 | FAM23A |
| 556 | LOC349196 | 1406 | NIPSNAP3B | 2256 | HHATL |
| 557 | C11orf80 | 1407 | CXADR | 2257 | PPEF1 |
| 558 | ANKRD36 | 1408 | GBE1 | 2258 | POMC |
| 559 | DPY19L2P2 | 1409 | ID1 | 2259 | C14orf135 |
| 560 | LYPLAL1 | 1410 | TNFAIP8L1 | 2260 | HBP1 |
| 561 | PDIA6 | 1411 | OR7E13P | 2261 | N4BP2 |
| 562 | SLC22A5 | 1412 | HIST1H2BC | 2262 | CHD1L |
| 563 | RALYL | 1413 | BBS2 | 2263 | TNFAIP1 |
| 564 | PHLDB2 | 1414 | G6PD | 2264 | TTC30B |
| 565 | PLA2R1 | 1415 | PACSIN3 | 2265 | TP63 |
| 566 | REPS2 | 1416 | PIK3R1 | 2266 | AVEN |
| 567 | NME5 | 1417 | SCARA3 | 2267 | FEM1C |
| 568 | ENOX1 | 1418 | LOC401397 | 2268 | NBEA |
| 569 | EMP3 | 1419 | TERC | 2269 | GALNT11 |
| 570 | SNAI2 | 1420 | TNXA | 2270 | ABHD5 |
| 571 | SLC38A2 | 1421 | MSH2 | 2271 | PKNOX2 |
| 572 | BICC1 | 1422 | SCXA | 2272 | LIMA1 |
| 573 | P2RY14 | 1423 | WDR52 | 2273 | MYL1 |
| 574 | SLC7A5 | 1424 | KCNN2 | 2274 | CCDC66 |
| 575 | HIST1H3E | 1425 | SEPN1 | 2275 | OAF |
| 576 | HEYL | 1426 | C9orf171 | 2276 | CLEC5A |
| 577 | DNAJA4 | 1427 | PTPRD | 2277 | PYGO1 |
| 578 | CHRM2 | 1428 | TP53INP2 | 2278 | PIGK |
| 579 | LOC440297 | 1429 | GAS8 | 2279 | CDKN1B |
| 580 | ANTXR1 | 1430 | CLYBL | 2280 | DPY19L2P4 |
| 581 | NT5DC2 | 1431 | ACADSB | 2281 | GPR3 |
| 582 | KLF10 | 1432 | DMKN | 2282 | IMPACT |
| 583 | GLIPR2 | 1433 | UPB1 | 2283 | SDPR |
| 584 | SEMA3D | 1434 | C6orf192 | 2284 | HLA-F |
| 585 | MAP2K3 | 1435 | SEC24D | 2285 | NAPEPLD |
| 586 | EXT1 | 1436 | RPLP2 | 2286 | C20orf194 |
| 587 | SNORD109A | 1437 | PODNL1 | 2287 | B4GALNT3 |
| 588 | BCKDHA | 1438 | TXLNB | 2288 | NEK3 |
| 589 | DGCR6 | 1439 | KIAA1598 | 2289 | SLC16A1 |
| 590 | APLP1 | 1440 | DPP3 | 2290 | SDK2 |
| 591 | BMP8A | 1441 | GSDMB | 2291 | PPP2R1B |
| 592 | HLTF | 1442 | MMP11 | 2292 | TMEM39A |
| 593 | TNFSF10 | 1443 | CTSF | 2293 | RANGAP1 |
| 594 | ZCCHC5 | 1444 | KIAA1377 | 2294 | ZNF132 |
| 595 | PLEKHB1 | 1445 | ACR | 2295 | C18orf54 |
| 596 | PHYHD1 | 1446 | GSTM2 | 2296 | PDCL3 |
| 597 | SAMD9L | 1447 | CDK2 | 2297 | C3orf26 |
| 598 | TEAD4 | 1448 | ITM2A | 2298 | SNORA6 |
| 599 | TCP11L2 | 1449 | ICA1L | 2299 | SEC61B |
| 600 | IFI44 | 1450 | TMED5 | 2300 | COX10 |
| 601 | IFIT3 | 1451 | DIXDC1 | 2301 | MAFF |
| 602 | STEAP3 | 1452 | COL4A1 | 2302 | DNM1P41 |
| 603 | DPY19L2P1 | 1453 | CHST3 | 2303 | FEZ2 |
| 604 | MTHFD2 | 1454 | FAM185A | 2304 | COQ3 |
| 605 | TLL1 | 1455 | SPR | 2305 | PRR16 |
| 606 | C20orf166 | 1456 | TUBA3C | 2306 | C21orf33 |
| 607 | ANKRD20B | 1457 | SNORD59A | 2307 | GEMIN8 |
| 608 | ST8SIA2 | 1458 | SNORD115-25 | 2308 | C12orf26 |
| 609 | EPHA3 | 1459 | ZNF730 | 2309 | HIST1H4E |
| 610 | CYP39A1 | 1460 | UQCRH | 2310 | PLXND1 |
| 611 | FHL2 | 1461 | NIPSNAP1 | 2311 | ACY1 |
| 612 | NR5A2 | 1462 | C14orf159 | 2312 | GATC |
| 613 | RPS6KA5 | 1463 | MIR30D | 2313 | KLHL24 |
| 614 | NUDT4 | 1464 | LGALS3BP | 2314 | TPM4 |
| 615 | STEAP2 | 1465 | TPD52L1 | 2315 | PORCN |
| 616 | SNORA61 | 1466 | RAB31 | 2316 | CYP11A1 |
| 617 | CACNB2 | 1467 | EHD2 | 2317 | C9orf174 |
| 618 | ITGA10 | 1468 | LACTB2 | 2318 | SLC7A11 |
| 619 | ABCA1 | 1469 | KLF5 | 2319 | DDR2 |
| 620 | FAM27D1 | 1470 | POP5 | 2320 | UQCRB |
| 621 | FBXO16 | 1471 | C14orf37 | 2321 | FGD6 |
| 622 | TMEM232 | 1472 | IFRD2 | 2322 | SDHC |
| 623 | TRUB1 | 1473 | RAD54B | 2323 | KIAA1161 |
| 624 | ASAM | 1474 | PLCL2 | 2324 | RPS27A |
| 625 | MX2 | 1475 | CEACAM19 | 2325 | ITGA9 |
| 626 | IRS2 | 1476 | LRRC59 | 2326 | RPSA |
| 627 | DCLK2 | 1477 | C9orf30 | 2327 | ZNF285 |
| 628 | C13orf40 | 1478 | PRIMA1 | 2328 | LYSMD1 |
| 629 | SGK1 | 1479 | APOBEC2 | 2329 | LENG8 |
| 630 | F10 | 1480 | IL3RA | 2330 | COBL |
| 631 | PLEKHO1 | 1481 | ACP6 | 2331 | SPRY1 |
| 632 | LGI2 | 1482 | FLJ13197 | 2332 | MMACHC |
| 633 | TUBA4A | 1483 | C10orf57 | 2333 | ARNT2 |
| 634 | PCNT | 1484 | C15orf41 | 2334 | TAS2R8 |
| 635 | C1QTNF7 | 1485 | SLC29A1 | 2335 | MCOLN3 |
| 636 | DDX60 | 1486 | SLFN11 | 2336 | CMIP |
| 637 | PCDH7 | 1487 | LOC100130331 | 2337 | R3HDM2 |
| 638 | NAV1 | 1488 | PEX7 | 2338 | ANXA4 |
| 639 | EPHA4 | 1489 | RWDD2B | 2339 | FUNDC1 |
| 640 | MCTP2 | 1490 | PDSS1 | 2340 | DPY19L4 |
| 641 | ENOSF1 | 1491 | PTCD2 | 2341 | CFHR2 |
| 642 | ITGB3 | 1492 | SLC4A4 | 2342 | C5orf36 |
| 643 | DLL4 | 1493 | PDK1 | 2343 | RPL10A |
| 644 | RAB3A | 1494 | NUDT17 | 2344 | OSGIN1 |
| 645 | GLT25D2 | 1495 | ABLIM2 | 2345 | GNRHR2 |
| 646 | RHOBTB1 | 1496 | GPC4 | 2346 | HIST2H2AC |
| 647 | DCUN1D3 | 1497 | THRB | 2347 | COG4 |
| 648 | PDE7B | 1498 | LYRM2 | 2348 | TBXA2R |
| 649 | DNALI1 | 1499 | FADS1 | 2349 | RABL5 |
| 650 | ZNF763 | 1500 | KDELC1 | 2350 | ABI3 |
| 651 | QSOX1 | 1501 | ZNF283 | 2351 | HMGN3 |
| 652 | THSD7A | 1502 | RPL23P8 | 2352 | MRPL36 |
| 653 | HLA-DQA2 | 1503 | CYB5A | 2353 | ASNS |
| 654 | GREB1L | 1504 | ARHGDIA | 2354 | FN3K |
| 655 | COL6A3 | 1505 | ZNF415 | 2355 | ADAM22 |
| 656 | SEMA4B | 1506 | ZNF259 | 2356 | TULP3 |
| 657 | SNORA9 | 1507 | CCNG1 | 2357 | TAF15 |
| 658 | NCKAP5 | 1508 | SNORD15A | 2358 | DGKE |
| 659 | GBP1 | 1509 | LDB2 | 2359 | ELK1 |
| 660 | MPPED2 | 1510 | LMLN | 2360 | LOC100506946 |
| 661 | FAM102B | 1511 | C5orf56 | 2361 | ANO5 |
| 662 | CC2D2B | 1512 | ZNF658 | 2362 | ATF4 |
| 663 | ARHGAP24 | 1513 | GPR124 | 2363 | SMCR7 |
| 664 | NCRNA00174 | 1514 | HAX1 | 2364 | SDCCAG3 |
| 665 | LRP2BP | 1515 | SERTAD3 | 2365 | ACOT1 |
| 666 | MIRLET7F1 | 1516 | PPP1R14B | 2366 | GYPE |
| 667 | HEPH | 1517 | REP15 | 2367 | NOP2 |
| 668 | AZIN1 | 1518 | TMEM159 | 2368 | ICMT |
| 669 | MGAT4C | 1519 | JUB | 2369 | PCBP3 |
| 670 | GCNT2 | 1520 | GIMAP2 | 2370 | RAD9B |
| 671 | TRIM22 | 1521 | C17orf75 | 2371 | NDUFAB1 |
| 672 | STXBP6 | 1522 | TMSB10 | 2372 | SLC25A26 |
| 673 | PGPEP1L | 1523 | WDR49 | 2373 | SGTB |
| 674 | KCND3 | 1524 | PROK1 | 2374 | CDC42EP1 |
| 675 | DLEU2 | 1525 | TAS2R14 | 2375 | SHMT2 |
| 676 | MX1 | 1526 | SDK1 | 2376 | ZBTB25 |
| 677 | MIR218-1 | 1527 | GPR75 | 2377 | RSU1 |
| 678 | PLXNA2 | 1528 | SLAIN1 | 2378 | TM7SF3 |
| 679 | HIST1H2BF | 1529 | TNXB | 2379 | TEAD3 |
| 680 | MYH10 | 1530 | TMEM55A | 2380 | ZSCAN16 |
| 681 | ALDH1A2 | 1531 | GDAP1 | 2381 | SCAMP2 |
| 682 | DBP | 1532 | TMEM182 | 2382 | NDUFA5 |
| 683 | PDE4DIP | 1533 | TAF4B | 2383 | RIMS1 |
| 684 | ZNF610 | 1534 | RASSF9 | 2384 | PPIP5K1 |
| 685 | CSPG4P5 | 1535 | CREB3L2 | 2385 | VPS37B |
| 686 | PVR | 1536 | GKAP1 | 2386 | TMED9 |
| 687 | TBX5 | 1537 | PIGN | 2387 | NDUFB6 |
| 688 | MYO10 | 1538 | LDHD | 2388 | MAGED1 |
| 689 | MIR29B2 | 1539 | MYLK3 | 2389 | DNAJC28 |
| 690 | ALCAM | 1540 | SEC11C | 2390 | MGC87042 |
| 691 | RGS6 | 1541 | KCNA2 | 2391 | C8orf44 |
| 692 | TMEM38A | 1542 | PRDX6 | 2392 | ORAI2 |
| 693 | HLA-DMA | 1543 | CNKSR3 | 2393 | SNX7 |
| 694 | ABCA8 | 1544 | LOC389607 | 2394 | ARL15 |
| 695 | TRIM45 | 1545 | NRN1 | 2395 | NACC2 |
| 696 | RDH5 | 1546 | GRK5 | 2396 | VCL |
| 697 | SPATA20 | 1547 | ID3 | 2397 | GRB2 |
| 698 | BTN3A2 | 1548 | TMEM14A | 2398 | PTP4A3 |
| 699 | LAMA4 | 1549 | PDE1C | 2399 | NOL3 |
| 700 | DCUN1D2 | 1550 | RBM43 | 2400 | TMEM53 |
| 701 | BCL2 | 1551 | CXXC4 | 2401 | RRBP1 |
| 702 | C4orf31 | 1552 | HRSP12 | 2402 | RAPH1 |
| 703 | NME1 | 1553 | EIF4A1 | 2403 | KLHDC1 |
| 704 | SYNC | 1554 | PAFAH2 | 2404 | CITED2 |
| 705 | PTGS1 | 1555 | IGF2BP2 | 2405 | AIFM2 |
| 706 | FGF12 | 1556 | DHRS11 | 2406 | SYDE1 |
| 707 | MBP | 1557 | SLC9A3R1 | 2407 | LARGE |
| 708 | ASB11 | 1558 | C11orf65 | 2408 | SRSF5 |
| 709 | KLHL7 | 1559 | EVC | 2409 | ANXA2P1 |
| 710 | SLC25A20 | 1560 | SORD | 2410 | CECR5 |
| 711 | HIST1H4H | 1561 | NELF | 2411 | TRIM41 |
| 712 | APBB3 | 1562 | ADAM15 | 2412 | MIF |
| 713 | SLC39A8 | 1563 | SLC38A6 | 2413 | FAM35A |
| 714 | SULT1C2 | 1564 | USH2A | 2414 | ARMC8 |
| 715 | COX7B | 1565 | C22orf46 | 2415 | PCSK5 |
| 716 | TRIM16L | 1566 | ZNF280B | 2416 | MIR1-1 |
| 717 | KLRD1 | 1567 | ANKS1B | 2417 | C3orf79 |
| 718 | SOCS2 | 1568 | ZNF546 | 2418 | DTWD2 |
| 719 | PDGFD | 1569 | MCF2 | 2419 | SEC23B |
| 720 | GOLGA6A | 1570 | CLCF1 | 2420 | DUSP16 |
| 721 | BMP6 | 1571 | SIK2 | 2421 | DDX55 |
| 722 | LRRC39 | 1572 | MRPS36 | 2422 | RCBTB2 |
| 723 | GJA1 | 1573 | UFSP2 | 2423 | TCEAL8 |
| 724 | PYROXD2 | 1574 | HN1 | 2424 | KCTD7 |
| 725 | SERPINH1 | 1575 | CATSPER2 | 2425 | CHST1 |
| 726 | ADAM28 | 1576 | ALPK2 | 2426 | LASP1 |
| 727 | LMOD2 | 1577 | SYTL2 | 2427 | TRAF3IP2 |
| 728 | DOCK5 | 1578 | IFI27L2 | 2428 | CD300E |
| 729 | NT5DC3 | 1579 | BPHL | 2429 | RIMKLB |
| 730 | ITGB8 | 1580 | IKBIP | 2430 | MAP2K5 |
| 731 | KRTAP5-2 | 1581 | CRTAC1 | 2431 | SRSF3 |
| 732 | TPCN1 | 1582 | PPP1R9A | 2432 | CDK14 |
| 733 | ANKMY2 | 1583 | C3orf33 | 2433 | DNM3 |
| 734 | CLU | 1584 | COLEC12 | 2434 | DHTKD1 |
| 735 | SEMA5A | 1585 | CSGALNACT2 | 2435 | ZNF277 |
| 736 | MIR221 | 1586 | SCAMP5 | 2436 | KCNMA1 |
| 737 | HSPA7 | 1587 | PROX1 | 2437 | PGF |
| 738 | STAMBPL1 | 1588 | EYA1 | 2438 | ARMCX1 |
| 739 | CD1C | 1589 | GPA33 | 2439 | FUCA1 |
| 740 | CCDC109B | 1590 | MTHFD2L | 2440 | SLC46A3 |
| 741 | TTLL7 | 1591 | ITPR3 | 2441 | SCN9A |
| 742 | LRRC8A | 1592 | C1orf152 | 2442 | TUB |
| 743 | PPP1R1C | 1593 | LRIG3 | 2443 | LARP6 |
| 744 | PHGDH | 1594 | ZNF737 | 2444 | PIH1D2 |
| 745 | SRPX | 1595 | GHR | 2445 | FAM174A |
| 746 | ADC | 1596 | DAND5 | 2446 | IFIH1 |
| 747 | SLC45A4 | 1597 | AGA | 2447 | TMEM161A |
| 748 | HTRA1 | 1598 | TJP2 | 2448 | SLC45A1 |
| 749 | C7orf41 | 1599 | ARHGAP15 | 2449 | IRAK4 |
| 750 | WASF1 | 1600 | ATP5L2 | 2450 | ATP6V1F |
| 751 | PITPNC1 | 1601 | PLXNB1 | 2451 | RHOQ |
| 752 | VPS8 | 1602 | ZNF230 | 2452 | NCRNA00258 |
| 753 | CRY1 | 1603 | ELTD1 | 2453 | EPHB4 |
| 754 | RAMP1 | 1604 | ZNF717 | 2454 | ELF4 |
| 755 | EXTL1 | 1605 | ZNF780B | 2455 | WDR35 |
| 756 | CALCRL | 1606 | ST3GAL4 | 2456 | ROPN1L |
| 757 | TAS2R20 | 1607 | C12orf32 | 2457 | UNG |
| 758 | CBLN1 | 1608 | TYRO3 | 2458 | FAM111A |
| 759 | NAV2 | 1609 | MYOM2 | 2459 | KIF27 |
| 760 | EMR2 | 1610 | TXN | 2460 | C10orf118 |
| 761 | PRDM5 | 1611 | LIPG | 2461 | BHLHB9 |
| 762 | PLSCR4 | 1612 | C4orf49 | 2462 | C15orf33 |
| 763 | PDGFRL | 1613 | CYP51A1 | 2463 | MOSC2 |
| 764 | DNAJC5G | 1614 | HYOU1 | 2464 | LIPT1 |
| 765 | LOC100128816 | 1615 | MYEF2 | 2465 | HN1L |
| 766 | ZNF434 | 1616 | GBP2 | 2466 | PIP4K2A |
| 767 | FAM60A | 1617 | CECR2 | 2467 | PRIM1 |
| 768 | PCDHB10 | 1618 | TMEM106C | 2468 | TTC8 |
| 769 | MTTP | 1619 | TCTN2 | 2469 | ADRA1B |
| 770 | S100A1 | 1620 | MYL6 | 2470 | HSD17B14 |
| 771 | TFPI | 1621 | CDC42EP2 | 2471 | MECOM |
| 772 | GXYLT2 | 1622 | RASAL2 | 2472 | ZNF187 |
| 773 | CD74 | 1623 | A4GALT | 2473 | LOC388796 |
| 774 | BDH1 | 1624 | GJC1 | 2474 | HNRNPA1L2 |
| 775 | E2F5 | 1625 | BCL2L11 | 2475 | HOOK2 |
| 776 | CKAP2 | 1626 | FAM173B | 2476 | STAC2 |
| 777 | ZNF300P1 | 1627 | TLE3 | 2477 | LRRC26 |
| 778 | POR | 1628 | SLC35E3 | 2478 | TSPAN6 |
| 779 | SKA2 | 1629 | XCL1 | 2479 | TMC4 |
| 780 | ATP8A1 | 1630 | EFCAB7 | 2480 | TGFBR2 |
| 781 | OLFM2 | 1631 | USP53 | 2481 | PACS1 |
| 782 | ANKRD34C | 1632 | CDKN3 | 2482 | FBXL4 |
| 783 | AMY1A | 1633 | FOXRED2 | 2483 | TRAK1 |
| 784 | RABGAP1L | 1634 | NFKB2 | 2484 | SGCD |
| 785 | EMP1 | 1635 | KRT18 | 2485 | MDFIC |
| 786 | MIR24-1 | 1636 | ZNF91 | 2486 | SHKBP1 |
| 787 | KLHL3 | 1637 | ATPIF1 | 2487 | ATP5SL |
| 788 | MAOA | 1638 | DKK2 | 2488 | EVL |
| 789 | S100A3 | 1639 | TAS2R13 | 2489 | PPP1R12C |
| 790 | ABCB4 | 1640 | FMNL2 | 2490 | FAM35B2 |
| 791 | ITGA3 | 1641 | EPDR1 | 2491 | ALS2CR4 |
| 792 | UBA7 | 1642 | C18orf19 | 2492 | TXNDC16 |
| 793 | CCDC21 | 1643 | ITPRIP | 2493 | ZNF627 |
| 794 | HS6ST1 | 1644 | TMUB1 | 2494 | ADAL |
| 795 | CCDC6 | 1645 | QDPR | 2495 | ZNF512 |
| 796 | PLAGL1 | 1646 | CD276 | 2496 | NDUFS4 |
| 797 | PXDNL | 1647 | MRTO4 | 2497 | GLYATL2 |
| 798 | LHFPL2 | 1648 | RUNX2 | 2498 | ITM2C |
| 799 | AP3M2 | 1649 | MAGED2 | 2499 | PXN |
| 800 | MIR107 | 1650 | ORC3 | 2500 | GALNT4 |
| 801 | H2AFZ | 1651 | DUSP7 | 2501 | B3GNT9 |
| 802 | SLC16A7 | 1652 | TMC7 | 2502 | RAB17 |
| 803 | PLEKHF1 | 1653 | CBFB | 2503 | SGCA |
| 804 | PTGDR | 1654 | LOC283588 | 2504 | VN1R1 |
| 805 | ZNF577 | 1655 | FXYD1 | 2505 | PMP2 |
| 806 | C11orf52 | 1656 | ARHGAP28 | 2506 | FZD5 |
| 807 | CSRNP3 | 1657 | PIK3C2B | 2507 | HDAC11 |
| 808 | CETN3 | 1658 | PLCB1 | 2508 | TEF |
| 809 | KCNJ5 | 1659 | FCER1A | 2509 | WDR1 |
| 810 | PDIA4 | 1660 | UCK2 | 2510 | SLC25A11 |
| 811 | DZIP3 | 1661 | CABLES2 | 2511 | XPO1 |
| 812 | FLJ35776 | 1662 | C12orf34 | 2512 | TNIP2 |
| 813 | SUSD2 | 1663 | NTN4 | 2513 | SHCBP1 |
| 814 | PLXNC1 | 1664 | SDCBP | 2514 | PQLC3 |
| 815 | JPH1 | 1665 | LTBP1 | 2515 | RPL22L1 |
| 816 | FRK | 1666 | IRAK1BP1 | 2516 | CA2 |
| 817 | KCNA5 | 1667 | PNMAL1 | 2517 | ZC3H11A |
| 818 | PKD1L2 | 1668 | HSDL1 | 2518 | MYOM1 |
| 819 | ASIP | 1669 | C11orf74 | 2519 | NRM |
| 820 | AMT | 1670 | FANCC | 2520 | FOXRED1 |
| 821 | KCNAB2 | 1671 | UBE2T | 2521 | PMM2 |
| 822 | ANXA2P2 | 1672 | TTPAL | 2522 | TUBD1 |
| 823 | CSPG4 | 1673 | USP12 | 2523 | DNAJC19 |
| 824 | COQ5 | 1674 | LIAS | 2524 | C8orf46 |
| 825 | C9orf106 | 1675 | GVINP1 | 2525 | CA5BP |
| 826 | SPATS2L | 1676 | DFNA5 | 2526 | ANTXR2 |
| 827 | FAM102A | 1677 | CCDC125 | 2527 | BRPF3 |
| 828 | PDLIM7 | 1678 | PFKM | 2528 | JMJD6 |
| 829 | CASP1 | 1679 | ABCC5 | 2529 | TTLL4 |
| 830 | RCN3 | 1680 | LOC728024 | 2530 | SDF2L1 |
| 831 | OR4F21 | 1681 | FEZ1 | 2531 | PTPLA |
| 832 | SQLE | 1682 | ABCB6 | 2532 | LDB3 |
| 833 | CDH23 | 1683 | ALDH6A1 | 2533 | PAAF1 |
| 834 | RTN4IP1 | 1684 | SLC35C1 | 2534 | ZNF527 |
| 835 | PCDHB18 | 1685 | ATPBD4 | 2535 | DRAM2 |
| 836 | AMD1 | 1686 | DUSP26 | 2536 | PDSS2 |
| 837 | EREG | 1687 | ZBED4 | 2537 | PTMS |
| 838 | MXRA8 | 1688 | HSPA1L | 2538 | TMX1 |
| 839 | BOC | 1689 | BAZ1A | 2539 | ELMOD2 |
| 840 | AS3MT | 1690 | ZC3H12B | 2540 | EID3 |
| 841 | DAPK2 | 1691 | C19orf10 | 2541 | MFN2 |
| 842 | LOC100127980 | 1692 | SLC25A33 |  |  |
| 843 | NHSL1 | 1693 | PTPRE |  |  |
| 844 | KCNJ4 | 1694 | FLJ44790 |  |  |
| 845 | SLITRK4 | 1695 | KLHL36 |  |  |
| 846 | NETO2 | 1696 | FAM82A1 |  |  |
| 847 | LRRC1 | 1697 | TUBB2A |  |  |
| 848 | CCT2 | 1698 | FAM190A |  |  |
| 849 | COL21A1 | 1699 | MCFD2 |  |  |
| 850 | LRRC4 | 1700 | KRBA2 |  |  |
