# Supplementary material for: Screening for immune-related biomarkers associated with myasthenia gravis and dilated cardiomyopathy based on bioinformatics analysis and machine learning
Source: Heliyon. 2024 Mar 20;10(7):e28446. doi: 10.1016/j.heliyon.2024.e28446 (PMC10988011; doi:10.1016/j.heliyon.2024.e28446)
Supplement: Multimedia component 5 [file mmc5.docx]

Table 5 GO analysis

| ONTOLOGY | ID | Description | pvalue |
| --- | --- | --- | --- |
| BP | GO:0002690 | positive regulation of leukocyte chemotaxis | 1.55E-05 |
| BP | GO:0071675 | regulation of mononuclear cell migration | 3.19E-05 |
| BP | GO:0002688 | regulation of leukocyte chemotaxis | 3.51E-05 |
| BP | GO:0050921 | positive regulation of chemotaxis | 5.12E-05 |
| BP | GO:0090026 | positive regulation of monocyte chemotaxis | 5.43E-05 |
| BP | GO:0002687 | positive regulation of leukocyte migration | 5.56E-05 |
| BP | GO:1905523 | positive regulation of macrophage migration | 8.38E-05 |
| BP | GO:0090025 | regulation of monocyte chemotaxis | 0.000104653 |
| BP | GO:0045766 | positive regulation of angiogenesis | 0.000110109 |
| BP | GO:1904018 | positive regulation of vasculature development | 0.000110109 |
| BP | GO:0071674 | mononuclear cell migration | 0.000142846 |
| BP | GO:0002685 | regulation of leukocyte migration | 0.000201618 |
| BP | GO:0050920 | regulation of chemotaxis | 0.000209576 |
| BP | GO:0030595 | leukocyte chemotaxis | 0.000240499 |
| BP | GO:0097529 | myeloid leukocyte migration | 0.000240499 |
| BP | GO:1905521 | regulation of macrophage migration | 0.0002428 |
| BP | GO:1905517 | macrophage migration | 0.000467405 |
| BP | GO:0060326 | cell chemotaxis | 0.00051287 |
| BP | GO:0002548 | monocyte chemotaxis | 0.000632883 |
| BP | GO:0071677 | positive regulation of mononuclear cell migration | 0.000650779 |
| BP | GO:0045765 | regulation of angiogenesis | 0.000711043 |
| BP | GO:1901342 | regulation of vasculature development | 0.000747183 |
| BP | GO:0050900 | leukocyte migration | 0.001003642 |
| BP | GO:0070098 | chemokine-mediated signaling pathway | 0.001036957 |
| BP | GO:1990868 | response to chemokine | 0.001225581 |
| BP | GO:1990869 | cellular response to chemokine | 0.001225581 |
| BP | GO:0007200 | phospholipase C-activating G protein-coupled receptor signaling pathway | 0.001592333 |
| BP | GO:0043524 | negative regulation of neuron apoptotic process | 0.002967286 |
| BP | GO:0007204 | positive regulation of cytosolic calcium ion concentration | 0.003933606 |
| BP | GO:0032640 | tumor necrosis factor production | 0.004195271 |
| BP | GO:0032680 | regulation of tumor necrosis factor production | 0.004195271 |
| BP | GO:0071706 | tumor necrosis factor superfamily cytokine production | 0.004419398 |
| BP | GO:1903555 | regulation of tumor necrosis factor superfamily cytokine production | 0.004419398 |
| BP | GO:0051351 | positive regulation of ligase activity | 0.005360624 |
| BP | GO:0043523 | regulation of neuron apoptotic process | 0.005826879 |
| BP | GO:1901215 | negative regulation of neuron death | 0.005826879 |
| BP | GO:0002887 | negative regulation of myeloid leukocyte mediated immunity | 0.00642964 |
| BP | GO:0042416 | dopamine biosynthetic process | 0.00642964 |
| BP | GO:0051340 | regulation of ligase activity | 0.00642964 |
| BP | GO:1900272 | negative regulation of long-term synaptic potentiation | 0.00642964 |
| BP | GO:1903977 | positive regulation of glial cell migration | 0.00642964 |
| BP | GO:2001234 | negative regulation of apoptotic signaling pathway | 0.006571057 |
| BP | GO:0002430 | complement receptor mediated signaling pathway | 0.006963761 |
| BP | GO:0071695 | anatomical structure maturation | 0.007356636 |
| BP | GO:0071361 | cellular response to ethanol | 0.007497622 |
| BP | GO:1904044 | response to aldosterone | 0.007497622 |
| BP | GO:0098883 | synapse pruning | 0.008031226 |
| BP | GO:0051402 | neuron apoptotic process | 0.008062537 |
| BP | GO:0014067 | negative regulation of phosphatidylinositol 3-kinase signaling | 0.009097658 |
| BP | GO:1904948 | midbrain dopaminergic neuron differentiation | 0.009097658 |
| BP | GO:0050665 | hydrogen peroxide biosynthetic process | 0.009630487 |
| BP | GO:0002281 | macrophage activation involved in immune response | 0.010163058 |
| BP | GO:0010759 | positive regulation of macrophage chemotaxis | 0.010163058 |
| BP | GO:0001666 | response to hypoxia | 0.010222722 |
| BP | GO:0042053 | regulation of dopamine metabolic process | 0.010695372 |
| BP | GO:0042069 | regulation of catecholamine metabolic process | 0.010695372 |
| BP | GO:1903975 | regulation of glial cell migration | 0.010695372 |
| BP | GO:0051607 | defense response to virus | 0.010902339 |
| BP | GO:0140546 | defense response to symbiont | 0.010971391 |
| BP | GO:0036293 | response to decreased oxygen levels | 0.011110089 |
| BP | GO:0007252 | I-kappaB phosphorylation | 0.011227428 |
| BP | GO:0030502 | negative regulation of bone mineralization | 0.011227428 |
| BP | GO:0044403 | biological process involved in symbiotic interaction | 0.011601736 |
| BP | GO:0009713 | catechol-containing compound biosynthetic process | 0.011759226 |
| BP | GO:0042423 | catecholamine biosynthetic process | 0.011759226 |
| BP | GO:0045723 | positive regulation of fatty acid biosynthetic process | 0.011759226 |
| BP | GO:0021700 | developmental maturation | 0.012030792 |
| BP | GO:0043171 | peptide catabolic process | 0.012290767 |
| BP | GO:0061760 | antifungal innate immune response | 0.012290767 |
| BP | GO:1901214 | regulation of neuron death | 0.01261377 |
| BP | GO:0043576 | regulation of respiratory gaseous exchange | 0.01282205 |
| BP | GO:0070482 | response to oxygen levels | 0.013059129 |
| BP | GO:0090023 | positive regulation of neutrophil chemotaxis | 0.013353077 |
| BP | GO:0001911 | negative regulation of leukocyte mediated cytotoxicity | 0.013883846 |
| BP | GO:0002407 | dendritic cell chemotaxis | 0.013883846 |
| BP | GO:0007026 | negative regulation of microtubule depolymerization | 0.013883846 |
| BP | GO:0045672 | positive regulation of osteoclast differentiation | 0.013883846 |
| BP | GO:0019216 | regulation of lipid metabolic process | 0.013893565 |
| BP | GO:0010758 | regulation of macrophage chemotaxis | 0.014944614 |
| BP | GO:0071624 | positive regulation of granulocyte chemotaxis | 0.014944614 |
| BP | GO:0150146 | cell junction disassembly | 0.014944614 |
| BP | GO:0002052 | positive regulation of neuroblast proliferation | 0.015474612 |
| BP | GO:0010575 | positive regulation of vascular endothelial growth factor production | 0.015474612 |
| BP | GO:0021952 | central nervous system projection neuron axonogenesis | 0.015474612 |
| BP | GO:0031342 | negative regulation of cell killing | 0.015474612 |
| BP | GO:0031114 | regulation of microtubule depolymerization | 0.016004354 |
| BP | GO:0070168 | negative regulation of biomineral tissue development | 0.016004354 |
| BP | GO:0070997 | neuron death | 0.016203131 |
| BP | GO:0071276 | cellular response to cadmium ion | 0.01653384 |
| BP | GO:2001233 | regulation of apoptotic signaling pathway | 0.016867812 |
| BP | GO:0001975 | response to amphetamine | 0.017063068 |
| BP | GO:0051482 | positive regulation of cytosolic calcium ion concentration involved in phospholipase C-activating G protein-coupled signaling pathway | 0.017063068 |
| BP | GO:0036336 | dendritic cell migration | 0.017592041 |
| BP | GO:0050901 | leukocyte tethering or rolling | 0.017592041 |
| BP | GO:1990776 | response to angiotensin | 0.017592041 |
| BP | GO:0033238 | regulation of amine metabolic process | 0.018120758 |
| BP | GO:0060074 | synapse maturation | 0.018120758 |
| BP | GO:0090022 | regulation of neutrophil chemotaxis | 0.018120758 |
| BP | GO:1902624 | positive regulation of neutrophil migration | 0.018120758 |
| BP | GO:0009615 | response to virus | 0.019197282 |
| BP | GO:0021955 | central nervous system neuron axonogenesis | 0.020233063 |
| BP | GO:0045923 | positive regulation of fatty acid metabolic process | 0.020233063 |
| BP | GO:0051385 | response to mineralocorticoid | 0.020233063 |
| BP | GO:0030279 | negative regulation of ossification | 0.0207605 |
| BP | GO:0031111 | negative regulation of microtubule polymerization or depolymerization | 0.0207605 |
| BP | GO:0042401 | biogenic amine biosynthetic process | 0.0207605 |
| BP | GO:0045730 | respiratory burst | 0.0207605 |
| BP | GO:0043434 | response to peptide hormone | 0.02091484 |
| BP | GO:0009309 | amine biosynthetic process | 0.021287682 |
| BP | GO:0010720 | positive regulation of cell development | 0.021377401 |
| BP | GO:0042417 | dopamine metabolic process | 0.021814608 |
| BP | GO:0048246 | macrophage chemotaxis | 0.021814608 |
| BP | GO:0043551 | regulation of phosphatidylinositol 3-kinase activity | 0.022341278 |
| BP | GO:0071542 | dopaminergic neuron differentiation | 0.022341278 |
| BP | GO:0042554 | superoxide anion generation | 0.022867694 |
| BP | GO:0032691 | negative regulation of interleukin-1 beta production | 0.023393854 |
| BP | GO:0007019 | microtubule depolymerization | 0.023919759 |
| BP | GO:1902622 | regulation of neutrophil migration | 0.023919759 |
| BP | GO:1902692 | regulation of neuroblast proliferation | 0.023919759 |
| BP | GO:0042551 | neuron maturation | 0.02444541 |
| BP | GO:0001774 | microglial cell activation | 0.024970805 |
| BP | GO:1900271 | regulation of long-term synaptic potentiation | 0.024970805 |
| BP | GO:0014075 | response to amine | 0.025495946 |
| BP | GO:0046189 | phenol-containing compound biosynthetic process | 0.025495946 |
| BP | GO:0002269 | leukocyte activation involved in inflammatory response | 0.026545465 |
| BP | GO:0042304 | regulation of fatty acid biosynthetic process | 0.026545465 |
| BP | GO:0022411 | cellular component disassembly | 0.026851124 |
| BP | GO:0001819 | positive regulation of cytokine production | 0.026953658 |
| BP | GO:0019221 | cytokine-mediated signaling pathway | 0.027262249 |
| BP | GO:0006968 | cellular defense response | 0.027593965 |
| BP | GO:0032692 | negative regulation of interleukin-1 production | 0.027593965 |
| BP | GO:0035176 | social behavior | 0.028117835 |
| BP | GO:0071622 | regulation of granulocyte chemotaxis | 0.028117835 |
| BP | GO:0043550 | regulation of lipid kinase activity | 0.02864145 |
| BP | GO:0046686 | response to cadmium ion | 0.02864145 |
| BP | GO:2000179 | positive regulation of neural precursor cell proliferation | 0.02864145 |
| BP | GO:0006584 | catecholamine metabolic process | 0.029164811 |
| BP | GO:0009712 | catechol-containing compound metabolic process | 0.029164811 |
| BP | GO:0042743 | hydrogen peroxide metabolic process | 0.029164811 |
| BP | GO:0051703 | biological process involved in intraspecies interaction between organisms | 0.029164811 |
| BP | GO:0061900 | glial cell activation | 0.029164811 |
| BP | GO:0007187 | G protein-coupled receptor signaling pathway, coupled to cyclic nucleotide second messenger | 0.029687919 |
| BP | GO:0050832 | defense response to fungus | 0.029687919 |
| BP | GO:0002931 | response to ischemia | 0.030210772 |
| BP | GO:0061756 | leukocyte adhesion to vascular endothelial cell | 0.030210772 |
| BP | GO:0002763 | positive regulation of myeloid leukocyte differentiation | 0.031255719 |
| BP | GO:0008347 | glial cell migration | 0.031255719 |
| BP | GO:0010574 | regulation of vascular endothelial growth factor production | 0.031255719 |
| BP | GO:0002886 | regulation of myeloid leukocyte mediated immunity | 0.031777813 |
| BP | GO:1903409 | reactive oxygen species biosynthetic process | 0.032299653 |
| BP | GO:0045428 | regulation of nitric oxide biosynthetic process | 0.03282124 |
| BP | GO:0010573 | vascular endothelial growth factor production | 0.033342574 |
| BP | GO:0080164 | regulation of nitric oxide metabolic process | 0.034384483 |
| BP | GO:0009620 | response to fungus | 0.035425382 |
| BP | GO:0002704 | negative regulation of leukocyte mediated immunity | 0.035945452 |
| BP | GO:0045670 | regulation of osteoclast differentiation | 0.035945452 |
| BP | GO:1901880 | negative regulation of protein depolymerization | 0.036984836 |
| BP | GO:0007405 | neuroblast proliferation | 0.038542021 |
| BP | GO:0006801 | superoxide metabolic process | 0.039060578 |
| BP | GO:0007585 | respiratory gaseous exchange by respiratory system | 0.039060578 |
| BP | GO:0045123 | cellular extravasation | 0.039060578 |
| BP | GO:0150076 | neuroinflammatory response | 0.040096939 |
| BP | GO:0006809 | nitric oxide biosynthetic process | 0.040614742 |
| BP | GO:0021536 | diencephalon development | 0.040614742 |
| BP | GO:0030500 | regulation of bone mineralization | 0.042166643 |
| BP | GO:0043242 | negative regulation of protein-containing complex disassembly | 0.042166643 |
| BP | GO:0071230 | cellular response to amino acid stimulus | 0.042166643 |
| BP | GO:0008344 | adult locomotory behavior | 0.042683441 |
| BP | GO:0046209 | nitric oxide metabolic process | 0.043716284 |
| BP | GO:0051851 | modulation by host of symbiont process | 0.043716284 |
| BP | GO:2001057 | reactive nitrogen species metabolic process | 0.044232329 |
| BP | GO:0021954 | central nervous system neuron development | 0.045263668 |
| BP | GO:1901879 | regulation of protein depolymerization | 0.045263668 |
| BP | GO:0046889 | positive regulation of lipid biosynthetic process | 0.045778962 |
| BP | GO:0031110 | regulation of microtubule polymerization or depolymerization | 0.046294005 |
| BP | GO:0071229 | cellular response to acid chemical | 0.046808799 |
| BP | GO:2000177 | regulation of neural precursor cell proliferation | 0.046808799 |
| BP | GO:0033555 | multicellular organismal response to stress | 0.047323342 |
| BP | GO:0030901 | midbrain development | 0.047837635 |
| BP | GO:0009791 | post-embryonic development | 0.048351678 |
| BP | GO:0001910 | regulation of leukocyte mediated cytotoxicity | 0.04989231 |
| BP | GO:0019217 | regulation of fatty acid metabolic process | 0.04989231 |
| CC | GO:0030667 | secretory granule membrane | 0.00064742 |
| CC | GO:0035579 | specific granule membrane | 0.001150571 |
| CC | GO:0042581 | specific granule | 0.003498909 |
| CC | GO:0043020 | NADPH oxidase complex | 0.010100409 |
| CC | GO:0097038 | perinuclear endoplasmic reticulum | 0.010658814 |
| CC | GO:0032809 | neuronal cell body membrane | 0.016227134 |
| CC | GO:0044298 | cell body membrane | 0.017892066 |
| CC | GO:0005765 | lysosomal membrane | 0.021848464 |
| CC | GO:0098852 | lytic vacuole membrane | 0.021848464 |
| CC | GO:0009897 | external side of plasma membrane | 0.022945784 |
| CC | GO:0005774 | vacuolar membrane | 0.026481185 |
| CC | GO:0043025 | neuronal cell body | 0.03091006 |
| CC | GO:0035577 | azurophil granule membrane | 0.032214533 |
| CC | GO:0070821 | tertiary granule membrane | 0.04039086 |
| CC | GO:0030670 | phagocytic vesicle membrane | 0.043102315 |
| CC | GO:0005791 | rough endoplasmic reticulum | 0.045266462 |
| MF | GO:0140375 | immune receptor activity | 6.98E-05 |
| MF | GO:0019956 | chemokine binding | 7.48E-05 |
| MF | GO:0001637 | G protein-coupled chemoattractant receptor activity | 9.71E-05 |
| MF | GO:0004950 | chemokine receptor activity | 9.71E-05 |
| MF | GO:0004896 | cytokine receptor activity | 0.001325178 |
| MF | GO:0019955 | cytokine binding | 0.002621719 |
| MF | GO:0008528 | G protein-coupled peptide receptor activity | 0.003117869 |
| MF | GO:0001653 | peptide receptor activity | 0.003381257 |
| MF | GO:0016175 | superoxide-generating NAD(P)H oxidase activity | 0.005973698 |
| MF | GO:0004875 | complement receptor activity | 0.007164538 |
| MF | GO:0035259 | nuclear glucocorticoid receptor binding | 0.007759471 |
| MF | GO:0046965 | nuclear retinoid X receptor binding | 0.008948365 |
| MF | GO:0050664 | oxidoreductase activity, acting on NAD(P)H, oxygen as acceptor | 0.009542327 |
| MF | GO:0004435 | phosphatidylinositol phospholipase C activity | 0.014282381 |
| MF | GO:0042974 | nuclear retinoic acid receptor binding | 0.014282381 |
| MF | GO:0070006 | metalloaminopeptidase activity | 0.015464167 |
| MF | GO:0004629 | phospholipase C activity | 0.016054578 |
| MF | GO:0043548 | phosphatidylinositol 3-kinase binding | 0.016644666 |
| MF | GO:0046982 | protein heterodimerization activity | 0.0171063 |
| MF | GO:0004177 | aminopeptidase activity | 0.023700676 |
| MF | GO:0004879 | nuclear receptor activity | 0.027211392 |
| MF | GO:0098531 | ligand-activated transcription factor activity | 0.027211392 |
| MF | GO:0008235 | metalloexopeptidase activity | 0.039986054 |
| MF | GO:0042379 | chemokine receptor binding | 0.043443484 |
| MF | GO:0001618 | virus receptor activity | 0.04516795 |
| MF | GO:0140272 | exogenous protein binding | 0.045742144 |
| MF | GO:0050660 | flavin adenine dinucleotide binding | 0.049752719 |
