# Supplementary material for: Screening for immune-related biomarkers associated with myasthenia gravis and dilated cardiomyopathy based on bioinformatics analysis and machine learning
Source: Heliyon. 2024 Mar 20;10(7):e28446. doi: 10.1016/j.heliyon.2024.e28446 (PMC10988011; doi:10.1016/j.heliyon.2024.e28446)
Supplement: Multimedia component 6 [file mmc6.docx]

Table 6 KEGG analysis

| ID | Description | pvalue |
| --- | --- | --- |
| hsa04061 | Viral protein interaction with cytokine and cytokine receptor | 0.001938477 |
| hsa04062 | Chemokine signaling pathway | 0.006977676 |
| hsa05171 | Coronavirus disease - COVID-19 | 0.010070395 |
| hsa04614 | Renin-angiotensin system | 0.015905619 |
| hsa04060 | Cytokine-cytokine receptor interaction | 0.016186376 |
