# Supplementary material for: Screening for immune-related biomarkers associated with myasthenia gravis and dilated cardiomyopathy based on bioinformatics analysis and machine learning
Source: Heliyon. 2024 Mar 20;10(7):e28446. doi: 10.1016/j.heliyon.2024.e28446 (PMC10988011; doi:10.1016/j.heliyon.2024.e28446)
Supplement: Multimedia component 7 [file mmc7.docx]

**Figure 1**

**WGCNA working sheets:**

setwd("WGCNA")

library(limma)

library(pheatmap)

library(ggplot2)

library(ggrepel)

library(tidyverse)

inputFile="exp_average.csv"

conFile="s1.txt"

treatFile="s2.txt"

logFCfilter=0.5

adj.P.Val.Filter=0.05

dimnames=list(rownames(exp),colnames(exp))

data=matrix(as.numeric(as.matrix(exp)),nrow=nrow(exp),dimnames=dimnames)

rt=avereps(data)

qx=as.numeric(quantile(rt, c(0, 0.25, 0.5, 0.75, 0.99, 1.0), na.rm=T))

LogC=( (qx[5]>100) || ( (qx[6]-qx[1])>50 && qx[2]>0) )

if(LogC){

rt[rt<0]=0

rt=log2(rt+1)}

data=normalizeBetweenArrays(rt)

sample1=read.table(conFile, header=F, sep="\t", check.names=F)

sample2=read.table(treatFile, header=F, sep="\t", check.names=F)

sampleName1=gsub("^ | $", "", as.vector(sample1[,1]))

sampleName2=gsub("^ | $", "", as.vector(sample2[,1]))

conData=data[,sampleName1]

treatData=data[,sampleName2]

data=cbind(conData,treatData)

conNum=ncol(conData)

treatNum=ncol(treatData)

dataend <- as.data.frame(data)

group_list <- c(rep("Control",conNum),rep("DCM",treatNum))

save(dataend,group_list,file = "step1-outputdata.Rdata")

library(WGCNA)

rm(list = ls())

getwd()

dir.create("./2.data quality")

setwd("2.data quality/")

load("../step1-outputdata.Rdata")

exp <- dataend

exp[1:4,1:4]

data <- exp

keep_data <- data[order(apply(data,1,mad), decreasing = T)[1:5000],]

datTraits <- data.frame(row.names = colnames(data),group=group_list)

datTraits$groupNO= ifelse(datTraits$group=="Control",'1','2')

datTraits<-as.data.frame(datTraits)

datExpr0 <- as.data.frame(t(keep_data))

datExpr0[1:4,1:4]

datTraits[1:4,1:2]

gsg <- goodSamplesGenes(datExpr0,verbose = 3)

gsg$allOK

if (!gsg$allOK){

# Optionally, print the gene and sample names that were removed:

if (sum(!gsg$goodGenes)>0)

printFlush(paste("Removing genes:", paste(names(datExpr0)[!gsg$goodGenes],

collapse = ", ")));

if (sum(!gsg$goodSamples)>0)

printFlush(paste("Removing samples:",

paste(rownames(datExpr0)[!gsg$goodSamples], collapse = ", ")));

# Remove the offending genes and samples from the data:

datExpr0 = datExpr0[gsg$goodSamples, gsg$goodGenes]

}

gsg <- goodSamplesGenes(datExpr0,verbose = 3)

gsg$allOK

sampleTree = hclust(dist(datExpr0), method = "average")

par(cex = 0.6)

par(mar = c(0,4,2,0))

plot(sampleTree)

plot(sampleTree, main = "Sample clustering to detect outliers", sub="", xlab="", cex.lab = 1.5, cex.axis = 1.5, cex.main = 2)

abline(h = 60, col = "red")

clust = cutreeStatic(sampleTree, cutHeight = 60, minSize = 10)

table(clust)

keepSamples = (clust==1)

datExpr0 = datExpr0[keepSamples, ]

dev.off()

sampleTree2 = hclust(dist(datExpr0), method = "average")

plot(sampleTree2)

nGenes = ncol(datExpr0)

nSamples = nrow(datExpr0)

save(datExpr0, nGenes, nSamples,file = "Step01-WGCNA_input.Rda")

enableWGCNAThreads()

powers = c(1:20)

sft = pickSoftThreshold(datExpr0, powerVector = powers, verbose = 5)

par(mfrow = c(1,2))

cex1 = 0.9

plot(sft$fitIndices[,1], -sign(sft$fitIndices[,3])*sft$fitIndices[,2],

xlab="Soft Threshold (power)",ylab="Scale Free Topology Model Fit,signed R^2",type="n",

main = paste("Scale independence"));

text(sft$fitIndices[,1], -sign(sft$fitIndices[,3])*sft$fitIndices[,2],

labels=powers,cex=cex1,col="red");

abline(h=0.90,col="red")

plot(sft$fitIndices[,1], sft$fitIndices[,5],

xlab="Soft Threshold (power)",ylab="Mean Connectivity", type="n",

main = paste("Mean connectivity"))

text(sft$fitIndices[,1], sft$fitIndices[,5], labels=powers, cex=cex1,col="red")

sft

softPower =sft$powerEstimate

#softPower = 2

adjacency = adjacency(datExpr0, power = softPower)

TOM = TOMsimilarity(adjacency)

dissTOM = 1-TOM

save(TOM,file = "TOM.Rda")

geneTree = hclust(as.dist(dissTOM), method = "average");

plot(geneTree, xlab="", sub="", main = "Gene clustering on TOM-based dissimilarity",

labels = FALSE, hang = 0.04)

minModuleSize = 30

dynamicMods = cutreeDynamic(dendro = geneTree, distM = dissTOM,

deepSplit = 2, pamRespectsDendro = FALSE,

minClusterSize = minModuleSize);

table(dynamicMods)

dynamicColors = labels2colors(dynamicMods)

table(dynamicColors)

plotDendroAndColors(geneTree, dynamicColors, "Dynamic Tree Cut",

dendroLabels = FALSE, hang = 0.03,

addGuide = TRUE, guideHang = 0.05,

main = "Gene dendrogram and module colors")

MEList = moduleEigengenes(datExpr0, colors = dynamicColors)

MEs = MEList$eigengenes

MEDiss = 1-cor(MEs);

METree = hclust(as.dist(MEDiss), method = "average")

plot(METree, main = "Clustering of module eigengenes",

xlab = "", sub = "")

MEDissThres = 0.78

abline(h=MEDissThres, col = "red")

merge = mergeCloseModules(datExpr0, dynamicColors, cutHeight = MEDissThres, verbose = 3)

mergedColors = merge$colors

mergedMEs = merge$newMEs

plotDendroAndColors(geneTree, mergedColors,"Dynamic Tree Cut",

dendroLabels = FALSE, hang = 0.03,

addGuide = TRUE, guideHang = 0.05,

main = "Gene dendrogram and module colors")

moduleColors = mergedColors

table(moduleColors)

colorOrder = c("grey", standardColors(50))

moduleLabels = match(moduleColors, colorOrder)-1

MEs = mergedMEs

dev.off()

clinical <- read.table("clinical.txt",sep = "\t",row.names = 1,check.names = F,stringsAsFactors = F,header = T)

clinical <- clinical[rownames(datExpr0),]

identical(rownames(clinical),rownames(datExpr0))

head(clinical)

datTraits = as.data.frame(do.call(cbind,lapply(clinical, as.numeric)))

rownames(datTraits) = rownames(clinical)

sampleTree2 = hclust(dist(datExpr0), method = "average")

traitColors = numbers2colors(datTraits, signed = FALSE)

plotDendroAndColors(sampleTree2,

traitColors,

groupLabels = names(datTraits),

main = "Sample dendrogram and trait heatmap")

dev.off()

MEs=orderMEs(MEs)

moduleTraitCor=cor(MEs, datTraits, use="p")

write.table(file="Step04-modPhysiological.cor.xls",moduleTraitCor,sep="\t",quote=F)

moduleTraitPvalue=corPvalueStudent(moduleTraitCor, nSamples)

write.table(file="Step04-modPhysiological.p.xls",moduleTraitPvalue,sep="\t",quote=F)

textMatrix=paste(signif(moduleTraitCor,2),"\n(",signif(moduleTraitPvalue,1),")",sep="")

dim(textMatrix)=dim(moduleTraitCor)

labeledHeatmap(Matrix=moduleTraitCor,

xLabels=colnames(datTraits),

yLabels=names(MEs),

ySymbols=names(MEs),

colorLabels=FALSE,

colors=blueWhiteRed(50),

textMatrix=textMatrix,

setStdMargins=FALSE,

cex.text=0.7,

cex.lab=0.7,

zlim=c(-1,1),

main=paste("Module-trait relationships"))

dev.off()

modNames = substring(names(MEs), 3)

geneModuleMembership = as.data.frame(cor(datExpr0, MEs, use = "p"))

a <- geneModuleMembership

a <- a %>% rownames_to_column()

MMPvalue = as.data.frame(corPvalueStudent(as.matrix(geneModuleMembership), nSamples))

names(geneModuleMembership) = paste("MM", modNames, sep="")

names(MMPvalue) = paste("p.MM", modNames, sep="")

traitNames=names(datTraits)

geneTraitSignificance = as.data.frame(cor(datExpr0, datTraits, use = "p"))

GSPvalue = as.data.frame(corPvalueStudent(as.matrix(geneTraitSignificance), nSamples))

names(geneTraitSignificance) = paste("GS.", traitNames, sep="")

names(GSPvalue) = paste("p.GS.", traitNames, sep="")

for (trait in traitNames){

traitColumn=match(trait,traitNames)

for (module in modNames){

column = match(module, modNames)

moduleGenes = moduleColors==module

if (nrow(geneModuleMembership[moduleGenes,]) > 1){

outPdf=paste(trait, "_", module,".pdf",sep="")

pdf(file=outPdf,width=7,height=7)

par(mfrow = c(1,1))

verboseScatterplot(abs(geneModuleMembership[moduleGenes, column]),

abs(geneTraitSignificance[moduleGenes, traitColumn]),

xlab = paste("Module Membership in", module, "module"),

ylab = paste("Gene significance for ",trait),

main = paste("Module membership vs. gene significance\n"),

cex.main = 1.2, cex.lab = 1.2, cex.axis = 1.2, col = module)

abline(v=0.8,h=0.5,col="red")

dev.off()

}

}

}

for (mod in 1:nrow(table(moduleColors)))

{

modules = names(table(moduleColors))[mod]

probes = colnames(datExpr0)

inModule = (moduleColors == modules)

modGenes = probes[inModule]

write.table(modGenes, file =paste0(modules,".txt"),sep="\t",row.names=F,col.names=F,quote=F)

}

**differential expression analysis:**

setwd("GSE112696")

library(limma)

library(pheatmap)

library(ggplot2)

library(ggrepel)

inputFile="exp_average.csv"

conFile="s1.txt"

treatFile="s2.txt"

logFCfilter=0.5

adj.P.Val.Filter=0.05

dimnames=list(rownames(exp),colnames(exp))

data=matrix(as.numeric(as.matrix(exp)),nrow=nrow(exp),dimnames=dimnames)

rt=avereps(data)

qx=as.numeric(quantile(rt, c(0, 0.25, 0.5, 0.75, 0.99, 1.0), na.rm=T))

LogC=( (qx[5]>100) || ( (qx[6]-qx[1])>50 && qx[2]>0) )

if(LogC){

rt[rt<0]=0

rt=log2(rt+1)}

data=normalizeBetweenArrays(rt)

sample1=read.table(conFile, header=F, sep="\t", check.names=F)

sample2=read.table(treatFile, header=F, sep="\t", check.names=F)

sampleName1=gsub("^ | $", "", as.vector(sample1[,1]))

sampleName2=gsub("^ | $", "", as.vector(sample2[,1]))

conData=data[,sampleName1]

treatData=data[,sampleName2]

data=cbind(conData,treatData)

conNum=ncol(conData)

treatNum=ncol(treatData)

Type=c(rep("Control",conNum), rep("MG",treatNum))

design <- model.matrix(~0+factor(Type))

colnames(design) <- c("Control","MG")

fit <- lmFit(data,design)

cont.matrix<-makeContrasts(MG-Control,levels=design)

fit2 <- contrasts.fit(fit, cont.matrix)

fit2 <- eBayes(fit2)

allDiff=topTable(fit2, adjust='fdr', number=200000)

allDiffOut <- allDiff

write.table(allDiffOut, file="all.txt", sep="\t", quote=F, col.names=T)

Type=c(rep("Control",conNum),rep("MG",treatNum))

outData=rbind(id=paste0(colnames(data),"_",Type),data)

write.table(outData, file="normalize.txt", sep="\t", quote=F, col.names=F)

diffSig=allDiff[with(allDiff, (abs(logFC)>logFCfilter & adj.P.Val < adj.P.Val.Filter )), ]

diffSigOut=rbind(id=colnames(diffSig),diffSig)

write.table(diffSigOut,file="diff.txt",sep="\t",quote=F,col.names=F)

**Figure 2**

**heatmap**

geneNum=50

diffSig=diffSig[order(as.numeric(as.vector(diffSig$logFC))),]

diffGeneName=as.vector(rownames(diffSig))

diffLength=length(diffGeneName)

hmGene=c()

if(diffLength>(2*geneNum)){

hmGene=diffGeneName[c(1:geneNum,(diffLength-geneNum+1):diffLength)]

}else{

hmGene=diffGeneName

}

hmExp=data[hmGene,]

Type=c(rep("Control",conNum),rep("MG",treatNum))

names(Type)=colnames(data)

Type=as.data.frame(Type)

pdf(file="Top-50heatmap.pdf", width=9, height=6.5)

pheatmap(hmExp,

annotation=Type,

color = colorRampPalette(c("blue", "white", "red"))(50),

cluster_cols =F,

show_colnames = F,

scale="row",

fontsize = 7,

fontsize_row=5,

fontsize_col=7)

dev.off()

**Figure 2**

**volcano plot**

allDiff$logFC[allDiff$logFC>20]=20

allDiff$logFC[allDiff$logFC< -20]=-20

Significant=ifelse((allDiff$adj.P.Val<adj.P.Val.Filter & abs(allDiff$logFC)>logFCfilter), ifelse(allDiff$logFC>logFCfilter,"Up","Down"), "Not")

geneNum=10

diffSig=diffSig[order(as.numeric(as.vector(diffSig$logFC))),]

diffGeneName=as.vector(rownames(diffSig))

diffLength=length(diffGeneName)

hmGene=c()

if(diffLength>(2*geneNum)){

hmGene=diffGeneName[c(1:geneNum,(diffLength-geneNum+1):diffLength)]

}else{

hmGene=diffGeneName

}

hmExp=data[hmGene,]

hmExp <- as.data.frame(hmExp)

Top10.genes <- c(as.character(rownames(hmExp)))

allDiff2 <- allDiff

allDiff2$Label = ""

allDiff2$Gene <- rownames(allDiff2)

allDiff2$Label[match(top10.genes, allDiff2$Gene)] <- top10.genes

p = ggplot(allDiff2, aes(logFC, -log10(adj.P.Val)))+

geom_point(aes(col=Significant))+

scale_color_manual(values=c("green", "black", "red"))+

labs(title = " ")+

theme(plot.title = element_text(size = 16, hjust = 0.5, face = "bold"))+

geom_hline(yintercept = -log10(0.05), linetype = "dashed") +

geom_vline(xintercept = c(-0.5, 0.5), linetype = "dashed")+

geom_label_repel(data = allDiff2,aes(logFC, -log10(adj.P.Val), label = Label),size = 3)

p=p+theme_bw()

pdf(file="Top10-vol.pdf", width=10.5, height=6)

print(p)

dev.off()

**Figure4 GO and KEGG analysis**

library(clusterProfiler)

library(org.Hs.eg.db)

library(enrichplot)

library(ggplot2)

library(circlize)

library(RColorBrewer)

library(dplyr)

library(ComplexHeatmap)

pvalueFilter=0.05

adjPvalFilter=1

colorSel="p.adjust"

if(adjPvalFilter>0.05){

colorSel="pvalue"

}

setwd("GO")

rt=read.table("interGene.txt", header=F, sep="\t", check.names=F)

genes=unique(as.vector(rt[,1]))

entrezIDs=mget(genes, org.Hs.egSYMBOL2EG, ifnotfound=NA)

entrezIDs=as.character(entrezIDs)

rt=cbind(rt, entrezIDs)

rt=rt[rt[,"entrezIDs"]!="NA",]

gene=rt$entrezID

#gene=gsub("c\\(\"(\\d+)\".*", "\\1", gene)

GO

kk=enrichGO(gene=gene, OrgDb=org.Hs.eg.db, pvalueCutoff=1, qvalueCutoff=1, ont="all", readable=T)

GO=as.data.frame(kk)

GO=GO[(GO$pvalue<pvalueFilter & GO$p.adjust<adjPvalFilter),]

write.table(GO, file="GO.txt", sep="\t", quote=F, row.names = F)

ontology.col=c("#00AFBB", "#E7B800", "#90EE90")

data=GO[order(GO$pvalue),]

datasig=data[data$pvalue<0.05,,drop=F]

BP = datasig[datasig$ONTOLOGY=="BP",,drop=F]

CC = datasig[datasig$ONTOLOGY=="CC",,drop=F]

MF = datasig[datasig$ONTOLOGY=="MF",,drop=F]

BP = head(BP,6)

CC = head(CC,6)

MF = head(MF,6)

data = rbind(BP,CC,MF)

main.col = ontology.col[as.numeric(as.factor(data$ONTOLOGY))]

BgGene = as.numeric(sapply(strsplit(data$BgRatio,"/"),'[',1))

Gene = as.numeric(sapply(strsplit(data$GeneRatio,'/'),'[',1))

ratio = Gene/BgGene

logpvalue = -log(data$pvalue,10)

logpvalue.col = brewer.pal(n = 8, name = "Reds")

f = colorRamp2(breaks = c(0,2,4,6,8,10,15,20), colors = logpvalue.col)

BgGene.col = f(logpvalue)

df = data.frame(GO=data$ID,start=1,end=max(BgGene))

rownames(df) = df$GO

bed2 = data.frame(GO=data$ID,start=1,end=BgGene,BgGene=BgGene,BgGene.col=BgGene.col)

bed3 = data.frame(GO=data$ID,start=1,end=Gene,BgGene=Gene)

bed4 = data.frame(GO=data$ID,start=1,end=max(BgGene),ratio=ratio,col=main.col)

bed4$ratio = bed4$ratio/max(bed4$ratio)*9.5

pdf("GO.circlize.pdf",width=10,height=10)

par(omi=c(0.1,0.1,0.1,1.5))

circos.par(track.margin=c(0.01,0.01))

circos.genomicInitialize(df,plotType="none")

circos.trackPlotRegion(ylim = c(0, 1), panel.fun = function(x, y) {

sector.index = get.cell.meta.data("sector.index")

xlim = get.cell.meta.data("xlim")

ylim = get.cell.meta.data("ylim")

circos.text(mean(xlim), mean(ylim), sector.index, cex = 0.8, facing = "bending.inside", niceFacing = TRUE)

}, track.height = 0.08, bg.border = NA,bg.col = main.col)

for(si in get.all.sector.index()) {

circos.axis(h = "top", labels.cex = 0.6, sector.index = si,track.index = 1,

major.at=seq(0,max(BgGene),by=100),labels.facing = "clockwise")

}

f = colorRamp2(breaks = c(-1, 0, 1), colors = c("green", "black", "red"))

circos.genomicTrack(bed2, ylim = c(0, 1),track.height = 0.1,bg.border="white",

panel.fun = function(region, value, ...) {

i = getI(...)

circos.genomicRect(region, value, ytop = 0, ybottom = 1, col = value[,2],

border = NA, ...)

circos.genomicText(region, value, y = 0.4, labels = value[,1], adj=0,cex=0.8,...)

})

circos.genomicTrack(bed3, ylim = c(0, 1),track.height = 0.1,bg.border="white",

panel.fun = function(region, value, ...) {

i = getI(...)

circos.genomicRect(region, value, ytop = 0, ybottom = 1, col = '#BA55D3',

border = NA, ...)

circos.genomicText(region, value, y = 0.4, labels = value[,1], cex=0.9,adj=0,...)

})

circos.genomicTrack(bed4, ylim = c(0, 10),track.height = 0.35,bg.border="white",bg.col="grey90",

panel.fun = function(region, value, ...) {

cell.xlim = get.cell.meta.data("cell.xlim")

cell.ylim = get.cell.meta.data("cell.ylim")

for(j in 1:9) {

y = cell.ylim[1] + (cell.ylim[2]-cell.ylim[1])/10*j

circos.lines(cell.xlim, c(y, y), col = "#FFFFFF", lwd = 0.3)

}

circos.genomicRect(region, value, ytop = 0, ybottom = value[,1], col = value[,2],

border = NA, ...)

#circos.genomicText(region, value, y = 0.3, labels = value[,1], ...)

})

circos.clear()

middle.legend = Legend(

labels = c('Number of Genes','Number of Select','Rich Factor(0-1)'),

type="points",pch=c(15,15,17),legend_gp = gpar(col=c('pink','#BA55D3',ontology.col[1])),

title="",nrow=3,size= unit(3, "mm")

)

circle_size = unit(1, "snpc")

draw(middle.legend,x=circle_size*0.42)

main.legend = Legend(

labels = c("Biological Process", "Cellular Component", "Molecular Function"), type="points",pch=15,

legend_gp = gpar(col=ontology.col), title_position = "topcenter",

title = "ONTOLOGY", nrow = 3,size = unit(3, "mm"),grid_height = unit(5, "mm"),

grid_width = unit(5, "mm")

)

logp.legend = Legend(

labels=c('(0,2]','(2,4]','(4,6]','(6,8]','(8,10]','(10,15]','(15,20]','>=20'),

type="points",pch=16,legend_gp=gpar(col=logpvalue.col),title="-log10(Pvalue)",

title_position = "topcenter",grid_height = unit(5, "mm"),grid_width = unit(5, "mm"),

size = unit(3, "mm")

)

lgd = packLegend(main.legend,logp.legend)

circle_size = unit(1, "snpc")

print(circle_size)

draw(lgd, x = circle_size*0.85, y=circle_size*0.55,just = "left")

dev.off()

setwd("KEGG")

library(GOplot)

library(clusterProfiler)

library(org.Hs.eg.db)

library(enrichplot)

library(ggplot2)

library(circlize)

library(RColorBrewer)

library(dplyr)

library(ComplexHeatmap)

rt=read.table("input.txt",sep="\t",check.names=F,header=T)

genes=as.vector(rt[,1])

entrezIDs=mget(genes, org.Hs.egSYMBOL2EG, ifnotfound=NA)

entrezIDs <- as.character(entrezIDs)

out=cbind(rt,entrezID=entrezIDs)

write.table(out,file="KEGG-id.txt",sep="\t",quote=F,row.names=F)

rt=read.table("KEGG-id.txt",sep="\t",header=T,check.names=F)

rt=rt[is.na(rt[,"entrezID"])==F,]

gene=rt$entrezID

kk <- enrichKEGG(gene = gene,keyType = "kegg",organism = "hsa", pvalueCutoff =1, qvalueCutoff =1, pAdjustMethod = "fdr")

write.table(kk,file="KEGG.txt",sep="\t",quote=F,row.names = F)

pdf(file="KEGG-bubble.pdf",width = 10,height = 13)

dotplot(kk, showCategory = 15,label_format=100)

dev.off()

**Figure5 Sankey**

library(ggalluvial)

library(ggplot2)

library(dplyr)

inputFile="input.txt"

outFile="ggalluvial.pdf"

setwd("Sankey")

rt=read.table(inputFile, header = T, sep="\t", check.names=F)

corLodes=to_lodes_form(rt, axes = 1:ncol(rt), id = "Cohort")

pdf(file="Sankey.pdf",width=7,height=6)

mycol <- rep(c("#029149","#6E568C","#E0367A","#D8D155","#223D6C","#D20A13","#431A3D","#91612D","#FFD121","#088247","#11AA4D","#58CDD9","#7A142C","#5D90BA","#64495D","#7CC767"),15)

ggplot(corLodes, aes(x = x, stratum = stratum, alluvium = Cohort,fill = stratum, label = stratum)) +

scale_x_discrete(expand = c(0, 0)) +

geom_flow(width = 2/10,aes.flow = "forward") +

geom_stratum(alpha = .9,width = 2/10) +

scale_fill_manual(values = mycol) +

geom_text(stat = "stratum", size = 2,color="black") +

xlab("") + ylab("") + theme_bw() +

theme(axis.line = element_blank(),axis.ticks = element_blank(),axis.text.y = element_blank()) +

theme(panel.grid =element_blank()) +

theme(panel.border = element_blank()) +

ggtitle("") + guides(fill = FALSE)

dev.off()

**Figure6 Lasso**

set.seed(1)

library(glmnet)

inputFile="normalize.txt"

geneFile="WGCNA-CGs-gene.txt"

setwd("lasso")

data=read.table(inputFile, header=T, sep="\t", check.names=F, row.names=1)

geneRT=read.table(geneFile, header=F, sep="\t", check.names=F)

data=data[as.vector(geneRT[,1]),]

data=t(data)

group=gsub("(.*)\\_(.*)", "\\2", row.names(data))

rt=as.data.frame(data)

rt$Type=ifelse(group=="Control", 0, 1)

x=as.matrix(rt[,1:(ncol(rt)-1)])

y=rt[,"Type"]

fit=glmnet(x, y, family = "binomial", alpha=1)

pdf(file="lasso.pdf",width=6,height=5.5)

plot(fit)

dev.off()

cvfit=cv.glmnet(x, y, family="binomial", alpha=1,type.measure='deviance',nfolds = 10)

pdf(file="cvfit.pdf",width=6,height=5.5)

plot(cvfit)

dev.off()

coef=coef(fit, s = cvfit$lambda.min)

index=which(coef != 0)

lassoGene=row.names(coef)[index]

lassoGene=lassoGene[-1]

write.table(lassoGene, file="lasso.gene.txt", sep="\t", quote=F, row.names=F, col.names=F)

outTab=rt[,c(lassoGene, "Type")]

outTab=cbind(id=row.names(outTab), outTab)

write.table(outTab, file="lasso.geneExp.txt", sep="\t", quote=F, row.names=F)

**Figure6 RF**

library(randomForest)

set.seed(123456)

setwd("random forest")

inputFile="lasso.geneExp.txt"

data=read.table(inputFile, header=T, sep="\t", check.names=F, row.names=1)

#data=t(data)

data <- data[,1:6]

group=gsub("(.*)\\_", "\\7", row.names(data))

rf=randomForest(as.factor(group)~., data=data, ntree=2000)

pdf(file="Forest.pdf", width=6, height=6)

plot(rf, main="Random forest", lwd=2)

dev.off()

optionTrees=which.min(rf$err.rate[,1])

optionTrees

rf2=randomForest(as.factor(group)~., data=data, ntree=optionTrees)

importance=importance(x=rf2)

pdf(file="GeneIm.pdf", width=6.2, height=5.8)

varImpPlot(rf2, main="")

dev.off()

rfGenes=importance[order(importance[,"MeanDecreaseGini"], decreasing = TRUE),]

rfGenes=names(rfGenes[rfGenes>6])

#rfGenes=names(rfGenes[1:30])

write.table(rfGenes, file="RF Genes.txt", sep="\t", quote=F, col.names=F, row.names=F)

sigExp=t(data[,rfGenes])

sigExpOut=rbind(ID=colnames(sigExp),sigExp)

write.table(sigExpOut, file="imGeneExp.txt", sep="\t", quote=F, col.names=F)

**Figure6 Gene expression**

setwd("modelgeneexp")

library(ggplot2) # Create Elegant Data Visualisations Using the Grammar of Graphics

library(rstatix)

library(ggsignif)

library(ggpubr)

df1 <- read.table("data1.txt", header = 1, check.names = F, sep = "\t")

df2 <- read.table("data2.txt", header = 1, check.names = F, sep = "\t")

df3 <- read.table("data3.txt", header = 1, check.names = F, sep = "\t")

df4 <- read.table("data4.txt", header = 1, check.names = F, sep = "\t")

p2 <- ggplot(df2, aes(Gene, value, fill = group))+

geom_boxplot(linewidth = 0.6)+

annotate("rect", xmin = 0.4, xmax = 1.5, ymin = -Inf, ymax = Inf, alpha = 0.2,fill="#c1f1fc") +

annotate("rect", xmin = 1.5, xmax = 2.5, ymin = -Inf, ymax = Inf, alpha = 0.2,fill="#ebffac") +

annotate("rect", xmin = 2.5, xmax = 3.5, ymin = -Inf, ymax = Inf, alpha = 0.2,fill="#53d769")+

annotate("rect", xmin = 3.5, xmax = 4.5, ymin = -Inf, ymax = Inf, alpha = 0.2,fill="#ffaaaa") +

annotate("rect", xmin = 4.5, xmax = 5.5, ymin = -Inf, ymax = Inf, alpha = 0.2,fill="#7B68EE")+ #("#c1f1fc","#ebffac","#53d769","#ffaaaa","#7B68EE","#66CDAA")

annotate("rect", xmin = 5.5, xmax = 6.6, ymin = -Inf, ymax = Inf, alpha = 0.2,fill="#66CDAA")+

#geom_dotplot(dotsize = 0.8,binaxis = "y", stackdir = "center",position = position_dodge(0.8))+

geom_vline(xintercept = 1.5, lty="dashed", color = "grey50", linewidth = 0.8)+

geom_vline(xintercept = 2.5, lty="dashed", color = "grey50", linewidth = 0.8)+

geom_vline(xintercept = 3.5, lty="dashed", color = "grey50", linewidth = 0.8)+

geom_vline(xintercept = 4.5, lty="dashed", color = "grey50", linewidth = 0.8)+

geom_vline(xintercept = 5.5, lty="dashed", color = "grey50", linewidth = 0.8)+

theme_bw()+

theme(axis.text.y = element_text(size=10, color = "#204056"),

axis.text.x = element_text(size=10, angle = 45, hjust = 1, vjust = 1, color = "#204056"),

axis.title = element_blank(),

panel.grid = element_blank())+

scale_fill_manual(values = c("#0ebeff", "#ae63e4" ))

stat.test <- df1 %>%

group_by(Gene) %>%

t_test(value ~ group) %>%

adjust_pvalue(method = "bonferroni") %>%

add_significance("p.adj")

stat.test

stat.test <- stat.test %>%

add_xy_position(x='Gene',dodge = 1)

#add_y_position()

p1 + stat_compare_means(method = "t.test",

label = "p.signif",

label.x = 'Gene',

label.y = c(10, 10, 10, 10, 10, 10))

dev.off()

**Figure7 Nomogram**

library(glmnet)

inputFile="lasso.geneExp.txt"

setwd("nomo")

rt=read.table(inputFile, header=T, sep="\t", check.names=F, row.names=1)

fit=glm(Type ~ ., family="binomial", data=rt)

summ=summary(fit)

newGene=row.names(summ$coefficients)[summ$coefficients[,"Pr(>|z|)"]<0.05]

rt2=rt[,c("Type", newGene)]

#rt2 <- as.data.frame(rt2)

fit2=glm(Type ~ ., family="binomial", data=rt2)

fit2=step(fit2)

conf=confint(fit2, level=0.95)

summ2=summary(fit2)

gene=row.names(summ2$coefficients)

coef=summ2$coefficients[,"Estimate"]

OR=exp(summ2$coefficients[,"Estimate"])

OR.95L=exp(conf[,1])

OR.95H=exp(conf[,2])

pvalue=summ2$coefficients[,"Pr(>|z|)"]

geneCoef=cbind(gene,coef, OR, OR.95L, OR.95H, pvalue)

write.table(geneCoef[-1,], file="modelGene.txt", sep="\t", quote=F, row.names=F)

pred=predict(fit2, type="response")

outTab=cbind(rt[,c("Type", gene[-1])], riskScore=pred)

outTab=cbind(id=row.names(outTab), outTab)

write.table(outTab, file="risk.txt", sep="\t", quote=F, row.names=F)

rm(list = ls())

library(rms)

library(rmda)

library(Hmisc)

riskFile="risk.txt"

cliFile="clinical.txt"

setwd("nomo")

data=read.table(riskFile, header=T, sep="\t", check.names=F, row.names=1)

cli=read.table(cliFile, header=T, sep="\t", check.names=F, row.names=1)

group=gsub("(.*?)\\_(.*)", "\\2", row.names(data))

row.names(data)=gsub("(.*?)\\_(.*)", "\\1", row.names(data))

sameSample=intersect(row.names(data), row.names(cli))

outTab=cbind(data[sameSample,1:(ncol(data)-1),drop=F], data[sameSample,"riskScore",drop=F])#cli[sameSample,,drop=F],

data=data[,2:(ncol(data)-1)]

data=cbind(data[sameSample,,drop=F])#cli[sameSample,,drop=F],

rt=cbind(as.data.frame(data), Type=group)

ddist=datadist(rt)

options(datadist="ddist")

lrmModel=lrm(Type~ ., data=rt, x=T, y=T)

nomo=nomogram(lrmModel, fun=plogis,

fun.at=c(0.0001,0.1,0.3,0.6,0.9,0.99),

lp=F, funlabel="Risk of Disease")

pdf("Nomo.pdf", width=9, height=5)

plot(nomo, cex.axis=0.8)

dev.off()

nomoRisk=predict(lrmModel, type="fitted")

outTab=cbind(outTab, Nomogram=nomoRisk)

outTab=rbind(id=colnames(outTab), outTab)

write.table(outTab, file="nomoRisk.txt", sep="\t", quote=F, col.names=F)

cali=calibrate(lrmModel, method="boot", B=1000)

pdf("Calibration.pdf", width=5.5, height=5.5)

plot(cali,

xlab="Predicted probability",

ylab="Actual probability", sub=F)

group=ifelse(group=="Control", 0, 1)

cindex=rcorrcens(group~nomoRisk)

se=cindex[,"SD"]/2

c_index=sprintf("%.03f", cindex[,"C"])

c_index.ci_low=sprintf("%.03f", cindex[,"C"]-(se*1.96))

c_index.ci_high=sprintf("%.03f", cindex[,"C"]+(se*1.96))

cindexLabel=paste0(c_index, " (95% CI: ", c_index.ci_low, "-", c_index.ci_high, ")")

text(0.1, 0.85, "C-index:")

text(0.28, 0.78, cindexLabel)

dev.off()

rm(list = ls())

library(glmnet)

library(pROC)

library(ggsci)

riskFile="nomoRisk.txt"

setwd("17.ROC-DCA")

rt=read.table(riskFile, header=T, sep="\t", check.names=F, row.names=1)

#rt$gender=ifelse(rt$gender=="female", 0, 1)

y=rt[,"Type"]

bioCol=pal_simpsons(palette=c("springfield"), alpha=1)(length(2:ncol(rt)))

aucText=c()

k=0

for(x in colnames(rt)[2:ncol(rt)]){

k=k+1

roc1=roc(y, as.numeric(rt[,x]))

if(k==1){

pdf(file="ROC.pdf", width=5.5, height=4.8)

plot(roc1, print.auc=F, col=bioCol[k], legacy.axes=T, main="")

aucText=c(aucText, paste0(x,", AUC=",sprintf("%.3f",roc1$auc[1])))

}else{

plot(roc1, print.auc=F, col=bioCol[k], legacy.axes=T, main="", add=TRUE)

aucText=c(aucText, paste0(x,", AUC=",sprintf("%.3f",roc1$auc[1])))

}

}

legend("bottomright", aucText, lwd=2, bty="n", col=bioCol, cex=0.7)

dev.off()

rm(list = ls())

library(rms)

library(rmda)

inputFile="nomoRisk.txt"

setwd("17.ROC-DCA")

rt=read.table(inputFile, header=T, sep="\t", check.names=F, row.names=1)

riskScore=decision_curve(Type ~ riskScore, data=rt,

family = binomial(link ='logit'),

thresholds= seq(0,1,by = 0.01),

confidence.intervals = 0.95)

Nomogram=decision_curve(Type ~ Nomogram, data=rt,

family = binomial(link ='logit'),

thresholds= seq(0,1,by = 0.01),

confidence.intervals = 0.95)

pdf(file="DCA.pdf", width=7.5, height=7)

plot_decision_curve(list(riskScore, Nomogram),#, Clinical

curve.names=c("riskScore", "Nomogram"), xlab="Threshold probability",

cost.benefit.axis=T,

confidence.intervals=FALSE,

standardize=FALSE)

dev.off()

**Figure8 Single gene GSEA**

library(ggplot2)

library(limma)

library(pheatmap)

library(ggsci)

lapply(c('clusterProfiler','enrichplot','patchwork'), function(x) {library(x, character.only = T)})

library(org.Hs.eg.db)

library(patchwork)

setwd("modelgeneGSEA")

expFile="exp.txt"

sgene="PIK3IP1"

dimnames=list(rownames(exp),colnames(exp))

data=matrix(as.numeric(as.matrix(exp)),nrow=nrow(exp),dimnames=dimnames)

data=avereps(data)

group <- ifelse(data[c(sgene),]>median(data[c(sgene),]), "High", "Low")

group <- factor(group,levels = c("High","Low"))

design <- model.matrix(~0+group)

colnames(design) <- levels(group)

fit <- lmFit(data,design)

cont.matrix<-makeContrasts(High-Low,levels=design)

fit2 <- contrasts.fit(fit, cont.matrix)

fit2 <- eBayes(fit2)

deg=topTable(fit2,adjust='fdr',number=nrow(data))

Diff=deg

DIFFOUT=rbind(id=colnames(Diff),Diff)

write.table(DIFFOUT,file=paste0("1.","DIFF_all.xls"),sep="\t",quote=F,col.names=F)

logFC_t=1

deg$g=ifelse(deg$P.Value>0.05,'stable',

ifelse( deg$logFC > logFC_t,'UP',

ifelse( deg$logFC < -logFC_t,'DOWN','stable') )

)

table(deg$g)

deg$symbol=rownames(deg)

df <- bitr(unique(deg$symbol), fromType = "SYMBOL",

toType = c( "ENTREZID"),

OrgDb = org.Hs.eg.db)

DEG=deg

DEG=merge(DEG,df,by.y='SYMBOL',by.x='symbol')

data_all_sort <- DEG %>%

arrange(desc(logFC))

geneList = data_all_sort$logFC

names(geneList) <- data_all_sort$ENTREZID

head(geneList)

kk2 <- gseKEGG(geneList = geneList,

organism = 'hsa',

nPerm = 10000,

minGSSize = 10,

maxGSSize = 200,

pvalueCutoff = 0.05,

pAdjustMethod = "none" )

class(kk2)

colnames(kk2@result)

kegg_result <- as.data.frame(kk2)

rownames(kk2@result)[head(order(kk2@result$enrichmentScore))]

af=as.data.frame(kk2@result)

write.table(af,file=paste0("2.","all_GSEA.xls"),sep="\t",quote=F,col.names=T)

gseaplot2(kk2,

title = "Th1 and Th2 cell differentiation",

"hsa04658",

color="red",

base_size = 20,

subplots = 1:3,

pvalue_table = T)

**Figure9 Cibersort**

library(e1071)

inputFile="normalize.txt"

setwd("MGCibersort")

source("geoARG24.CIBERSORT.R")

outTab=CIBERSORT("ref.txt", inputFile, perm=1000, QN=T)

outTab=outTab[outTab[,"P-value"]<0.05,]

outTab=as.matrix(outTab[,1:(ncol(outTab)-3)])

outTab=rbind(id=colnames(outTab),outTab)

write.table(outTab, file="CIBERSORT-Results.txt", sep="\t", quote=F, col.names=F)

rm(list = ls())

library(reshape2)

library(ggpubr)

inputFile="CIBERSORT-Results.txt"

setwd("CIBERSORT")

rt=read.table(inputFile, header=T, sep="\t", check.names=F, row.names=1)

con=grepl("_Control", rownames(rt), ignore.case=T)

treat=grepl("_MG", rownames(rt), ignore.case=T)

conData=rt[con,]

treatData=rt[treat,]

conNum=nrow(conData)

treatNum=nrow(treatData)

data=t(rbind(conData,treatData))

Type=gsub("(.*)\\_(.*)", "\\2", rownames(rt))

data=cbind(as.data.frame(t(data)), Type)

data=melt(data, id.vars=c("Type"))

colnames(data)=c("Type", "Immune", "Expression")

group=levels(factor(data$Type))

bioCol=c("#008B45FF","#EE0000FF","#0066FF","#FF0000","#6E568C","#7CC767","#223D6C","#D20A13","#FFD121","#088247","#11AA4D")

bioCol=bioCol[1:length(group)]

boxplot=ggboxplot(data, x="Immune", y="Expression", fill="Type",

xlab="",

ylab="Fraction",

legend.title="Type",

notch=T,

#add="point",

width=0.8,

palette=bioCol)+

rotate_x_text(50)+

stat_compare_means(aes(group=Type),symnum.args=list(cutpoints=c(0, 0.001, 0.01, 0.05, 1), symbols=c("***", "**", "*", "")), label="p.signif")

pdf(file="immune.diff.pdf", width=8, height=6)

print(boxplot)

dev.off()

rm(list = ls())

library(limma)

library(reshape2)

library(tidyverse)

library(ggplot2)

expFile="normalize.txt"

geneFile="Hubgene.txt"

immFile="CIBERSORT-Results.txt"

setwd("CIBERSORT")

rt=read.table(expFile, header=T, sep="\t", check.names=F)

rt=as.matrix(rt)

rownames(rt)=rt[,1]

exp=rt[,2:ncol(rt)]

dimnames=list(rownames(exp),colnames(exp))

data=matrix(as.numeric(as.matrix(exp)),nrow=nrow(exp),dimnames=dimnames)

data=avereps(data)

geneRT=read.table(geneFile, header=T, sep="\t", check.names=F)

data=data[as.vector(geneRT[,1]),]

group=gsub("(.*)\\_(.*)", "\\2", colnames(data))

data=data[,group=="MG",drop=F]

data=t(data)

#data <- as.data.frame(data)

immune=read.table(immFile, header=T, sep="\t", check.names=F, row.names=1)

sameSample=intersect(row.names(data), row.names(immune))

data=data[sameSample,,drop=F]

immune=immune[sameSample,,drop=F]

outTab=data.frame()

for(cell in colnames(immune)){

if(sd(immune[,cell])==0){next}

for(gene in colnames(data)){

x=as.numeric(immune[,cell])

y=as.numeric(data[,gene])

corT=cor.test(x,y,method="spearman")

cor=corT$estimate

pvalue=corT$p.value

text=ifelse(pvalue<0.001,"***",ifelse(pvalue<0.01,"**",ifelse(pvalue<0.05,"*","")))

outTab=rbind(outTab,cbind(Gene=gene, Immune=cell, cor, text, pvalue))

}

}

outTab$cor=as.numeric(outTab$cor)

pdf(file="cor.pdf", width=7, height=4.8)

ggplot(outTab, aes(Immune, Gene)) +

geom_tile(aes(fill = cor), colour = "grey", size = 1)+

scale_fill_gradient2(low = "#5C5DAF", mid = "white", high = "#EA2E2D") +

geom_text(aes(label=text),col ="black",size = 3) +

theme_minimal() +

theme(axis.title.x=element_blank(), axis.ticks.x=element_blank(), axis.title.y=element_blank(),

axis.text.x = element_text(angle = 45, hjust = 1, size = 8, face = "bold"),

axis.text.y = element_text(size = 8, face = "bold")) +

labs(fill =paste0("*** p<0.001","\n", "** p<0.01","\n", " * p<0.05","\n", "\n","Correlation")) +

scale_x_discrete(position = "bottom")

dev.off()

rm(list = ls())
